# Supplementary material for: Novel Sophoridine Derivatives as Potential Larvicidal Agents against Aedes albopictus: Synthesis, Biological Evaluation, Acetylcholinesterase Inhibition, and Morphological Study
Source: Insects. 2023 Apr 20;14(4):399. doi: 10.3390/insects14040399 (PMC10140878; doi:10.3390/insects14040399)
Supplement: Supplementary file 1 [file insects-14-00399-s001.zip › insects-2337971-supplementary.pdf]

S1. Structural characterization data (1H NMR, 13C NMR, and HRESIMS) of SOP-2c-SOP-2r and SOP-3c-SOP-3f.

S1.1 (*E*)-10-chloro-2,3,4^1,6,7,7a,12,13,13a,13b-decahydro-1*H*,5*H*,8*H*-dipyrido [2,1-*f*:3',2',1'-*ij*][1,6]naphthyridine-11-carbaldehyde *O*-benzoyl oxime (**SOP-2a**, $C_{23}H_{28}ClN_3O_2$)

The compound was obtained in 80% yield as a white solid. ^{1}H NMR (500 MHz, $CDCl_3$) δ 8.60 (s, 1H), 8.09 (dd, *J* = 8.3, 1.2 Hz, 2H), 7.61 – 7.54 (m, 1H), 7.49 – 7.43 (m, 2H), 3.61 (dd, *J* = 12.2, 4.5 Hz, 1H), 3.06 (d, *J* = 11.6 Hz, 1H), 3.01 – 2.85 (m, 3H), 2.71 (dd, *J* = 11.2, 5.4 Hz, 1H), 2.65 – 2.59 (m, 1H), 2.52 – 2.43 (m, 2H), 2.37 (t, *J* = 11.9 Hz, 1H), 2.22 (dtt, *J* = 15.7, 7.4, 4.2 Hz, 1H), 1.98 – 1.92 (m, 1H), 1.91 – 1.82 (m, 2H), 1.79 (ddd, *J* = 13.7, 6.7, 3.8 Hz, 2H), 1.72 (dd, *J* = 12.8, 3.7 Hz, 1H), 1.66 (ddd, *J* = 16.8, 7.9, 3.5 Hz, 2H), 1.50 (dd, *J* = 12.6, 3.2 Hz, 1H), 1.34 (d, *J* = 12.6 Hz, 1H), 1.10 (qd, *J* = 12.7, 4.1 Hz, 1H). ^{13}C NMR (125 MHz, $CDCl_3$) δ 164.44, 156.81, 149.81, 146.92, 133.22, 129.71, 129.15, 128.56, 109.60, 106.67, 63.44, 59.63, 54.82, 51.71, 45.94, 40.17, 29.77, 26.19, 25.61, 25.51, 23.15, 19.28. HRMS (ESI): $C_{23}H_{29}ClN_3O_2$ (414.1943) $[M+H]^+$ = 414.1947.

S1.2 (*E*)-10-chloro-2,3,4^1,6,7,7a,12,13,13a,13b-decahydro-1*H*,5*H*,8*H*-dipyrido [2,1-*f*:3',2',1'-*ij*][1,6]naphthyridine-11-carbaldehyde *O*-(2-methylbenzoyl) oxime (**SOP-2b**, $C_{24}H_{30}ClN_3O_2$)

The compound was obtained in 72% yield as a white solid. ^{1}H NMR (500 MHz, $CDCl_3$) δ 7.89 (d, *J* = 8.0 Hz, 1H), 7.40 (td, *J* = 7.7, 1.3 Hz, 1H), 7.25 (t, *J* = 7.8 Hz, 2H), 3.59 (dd, *J* = 12.2, 4.5 Hz, 1H), 3.05 (d, *J* = 11.6 Hz, 1H), 2.99 – 2.94 (m, 1H), 2.95 – 2.81 (m, 2H), 2.68 (dd, *J* = 11.2, 5.4 Hz, 1H), 2.61 (s, 4H), 2.52 – 2.43 (m, 2H), 2.36 (t, *J* = 11.8 Hz, 1H), 2.20 (dq, *J* = 8.1, 5.7, 4.1 Hz, 1H), 1.95 – 1.81 (m, 3H), 1.78 (ddt, *J* = 14.6, 7.6, 3.7 Hz, 3H), 1.73 – 1.56 (m, 3H), 1.52 – 1.45 (m, 1H), 1.31 (d, *J* = 14.0 Hz, 1H), 1.09 (qd, *J* = 12.8, 4.2 Hz, 1H). ^{13}C NMR (125 MHz, $CDCl_3$) δ 165.47, 156.65, 146.86, 140.35, 132.15, 131.77, 130.25, 128.66, 125.78, 109.78, 63.46, 59.60, 54.77, 51.66, 45.90, 40.11, 29.71, 26.17, 25.59, 25.47, 24.75, 23.10, 21.57, 19.23. HRMS (ESI): $C_{24}H_{31}ClN_3O_2$ (428.2099) $[M+H]^+$ = 428.2105.

S1.3 (*E*)-10-chloro-2,3,4^1,6,7,7a,12,13,13a,13b-decahydro-1*H*,5*H*,8*H*-dipyrido

[2,1-*f*:3',2',1'-*ij*][1,6]naphthyridine-11-carbaldehyde *O*-(3-methylbenzoyl) oxime (**SOP-2c**, $C_{24}H_{30}ClN_3O_2$)

The compound was obtained in 68% yield as a white solid. ^{1}H NMR (500 MHz, $CDCl_3$) δ 8.60 (s, 1H), 7.93 – 7.84 (m, 2H), 7.41 – 7.29 (m, 2H), 3.61 (dd, *J* = 12.2, 4.5 Hz, 1H), 3.06 (d, *J* = 11.5 Hz, 1H), 3.01 – 2.83 (m, 3H), 2.69 (dd, *J* = 11.2, 5.4 Hz, 1H), 2.66 – 2.59 (m, 1H), 2.48 (ddd, *J* = 17.5, 11.1, 7.6 Hz, 2H), 2.42 (s, 3H), 2.38 (t, *J* = 11.9 Hz, 1H), 2.21 (tdd, *J* = 15.3, 11.6, 4.2 Hz, 1H), 1.93 (dd, *J* = 11.0, 4.4 Hz, 1H), 1.86 (ddd, *J* = 17.5, 7.0, 4.6 Hz, 2H), 1.79 (td, *J* = 10.4, 9.7, 5.9 Hz, 2H), 1.72 (dd, *J* = 12.7, 3.5 Hz, 1H), 1.69 – 1.57 (m, 2H), 1.53 – 1.47 (m, 1H), 1.32 (d, *J* = 10.3 Hz, 1H), 1.10 (qd, *J* = 12.7, 4.1 Hz, 1H). ^{13}C NMR (125 MHz, $CDCl_3$) δ 164.44, 156.80, 146.92, 133.22, 129.71, 129.15, 128.55, 109.60, 106.67, 63.44, 59.63, 54.81, 51.71, 45.94, 40.17, 29.77, 26.19, 25.61, 25.51, 24.74, 23.15, 19.28. HRMS (ESI): $C_{24}H_{31}ClN_3O_2$ (428.2099) $[M+H]^+$ = 428.2102.

S1.4 (*E*)-10-chloro-2,3,4^1,6,7,7a,12,13,13a,13b-decahydro-1*H*,5*H*,8*H*-dipyrido [2,1-*f*:3',2',1'-*ij*][1,6]naphthyridine-11-carbaldehyde *O*-(4-methylbenzoyl) oxime (**SOP-2d**, $C_{24}H_{30}ClN_3O_2$)

The compound was obtained in 80% yield as a yellow solid. ^{1}H NMR (500 MHz, $CDCl_3$) δ 8.59 (s, 1H), 7.98 (d, *J* = 8.2 Hz, 2H), 7.25 (d, *J* = 7.8 Hz, 2H), 3.60 (dd, *J* = 12.2, 4.5 Hz, 1H), 3.05 (d, *J* = 11.6 Hz, 1H), 3.00 – 2.83 (m, 3H), 2.68 (dd, *J* = 11.2, 5.4 Hz, 1H), 2.65 – 2.59 (m, 1H), 2.52 – 2.43 (m, 2H), 2.41 (s, 3H), 2.36 (t, *J* = 11.9 Hz, 1H), 2.21 (dtt, *J* = 15.4, 7.3, 4.1 Hz, 1H), 1.95 – 1.82 (m, 3H), 1.78 (ddt, *J* = 13.1, 6.1, 3.3 Hz, 2H), 1.71 (dd, *J* = 12.9, 3.9 Hz, 1H), 1.70 – 1.55 (m, 2H), 1.52 – 1.46 (m, 1H), 1.31 (d, *J* = 15.8 Hz, 1H), 1.09 (qd, *J* = 12.8, 4.2 Hz, 1H). ^{13}C NMR (125 MHz, $CDCl_3$) δ 164.53, 156.64, 146.83, 143.98, 129.76, 129.29, 126.32, 109.88, 63.49, 59.60, 54.79, 51.69, 45.90, 40.15, 29.75, 26.16, 25.64, 25.52, 24.81, 23.10, 21.83, 19.23. HRMS (ESI): $C_{24}H_{31}ClN_3O_2$ (428.2089) $[M+H]^+$ = 428.2104.

S1.5 (*E*)-10-chloro-2,3,4^1,6,7,7a,12,13,13a,13b-decahydro-1*H*,5*H*,8*H*-dipyrido [2,1-*f*:3',2',1'-*ij*][1,6]naphthyridine-11-carbaldehyde *O*-cinnamoyl oxime (**SOP-2e**, $C_{25}H_{30}ClN_3O_2$)

The compound was obtained in 80% yield as a white solid. ^{1}H NMR (500 MHz, $CDCl_3$) δ 8.52 (s, 1H), 7.80 (d, J = 16.0 Hz, 1H), 7.58 – 7.52 (m, 2H), 7.43 – 7.36 (m, 3H), 6.51 (d, J = 16.0 Hz, 1H), 3.59 (dd, J = 12.2, 4.4 Hz, 1H), 3.04 (d, J = 11.6 Hz, 1H), 3.00 – 2.82 (m, 3H), 2.68 (dd, J = 11.2, 5.4 Hz, 1H), 2.59 (dd, J = 16.5, 5.6 Hz, 1H), 2.51 – 2.39 (m, 2H), 2.35 (t, J = 11.9 Hz, 1H), 2.21 (ddt, J = 15.4, 8.3, 3.9 Hz, 1H), 1.96 – 1.89 (m, 1H), 1.89 – 1.81 (m, 2H), 1.78 (ddd, J = 14.3, 6.9, 4.3 Hz, 2H), 1.74 – 1.68 (m, 1H), 1.68 – 1.57 (m, 2H), 1.52 – 1.44 (m, 1H), 1.31 (d, J = 13.3 Hz, 1H), 1.09 (qd, J = 12.8, 4.2 Hz, 1H). ^{13}C NMR (125 MHz, $CDCl_3$) δ 165.06, 156.35, 146.85, 145.78, 134.49, 130.60, 129.04, 128.30, 116.01, 109.82, 63.47, 59.64, 54.81, 51.69, 45.93, 40.15, 29.74, 26.19, 25.62, 25.50, 24.78, 23.09, 19.25. HRMS (ESI): $C_{25}H_{31}ClN_3O_2$ (440.2099) $[M+H]^+$ = 440.2097.

S1.6 (*E*)-10-chloro-2,3,41,6,7,7a,12,13,13a,13b-decahydro-1*H*,5*H*,8*H*-dipyri- do[2,1-*f*:3',2',1'-*ij*][1,6]naphthyridine-11-carbaldehyde *O*-(3-phenylpropanoyl) oxime (**SOP-2f**, $C_{25}H_{32}ClN_3O_2$)

The compound was obtained in 78% yield as a yellow solid. ^{1}H NMR (500 MHz, $CDCl_3$) δ 8.38 (s, 1H), 7.29 (t, J = 7.4 Hz, 2H), 7.21 (dd, J = 14.2, 7.2 Hz, 3H), 3.58 (dd, J = 12.2, 4.3 Hz, 1H), 3.05 – 2.98 (m, 3H), 2.98 – 2.85 (m, 3H), 2.74 – 2.69 (m, 3H), 2.52 (dd, J = 16.5, 6.6 Hz, 2H), 2.43 – 2.36 (m, 1H), 2.36 – 2.30 (m, 1H), 2.25 – 2.16 (m, 1H), 1.98 – 1.91 (m, 1H), 1.90 – 1.75 (m, 4H), 1.71 (dd, J = 12.5, 3.2 Hz, 1H), 1.69 – 1.61 (m, 2H), 1.52 – 1.45 (m, 1H), 1.34 (d, J = 13.8 Hz, 1H), 1.09 (qd, J = 12.8, 4.1 Hz, 1H). ^{13}C NMR (125 MHz, $CDCl_3$) δ 171.74, 155.96, 146.61, 110.35, 63.45, 59.54, 54.51, 51.45, 45.76, 39.73, 33.05, 31.90, 29.36, 29.30, 29.24, 29.21, 26.23, 25.42, 25.13, 25.03, 24.82, 22.84, 22.74, 19.02, 14.21. HRMS (ESI): $C_{25}H_{33}ClN_3O_2$ (442.2256) $[M +H]^+$ = 442.2259.

S1.7 (*E*)-10-chloro-2,3,4^1,6,7,7a,12,13,13a,13b-decahydro-1*H*,5*H*,8*H*-dipyrido [2,1-*f*:3',2',1'-*ij*][1,6]naphthyridine-11-carbaldehyde *O*-(1-naphthoyl) oxime (**SOP-2g**, $C_{27}H_{30}ClN_3O_2$)

The compound was obtained in 80% yield as a pale-yellow solid. ^{1}H NMR (500 MHz, $CDCl_3$) δ 8.34 (s, 1H), 8.04 (d, J = 7.9 Hz, 1H), 7.90 – 7.84 (m, 1H), 7.83 – 7.78 (m, 1H), 7.57 – 7.48 (m, 2H), 7.46 – 7.39 (m, 2H), 4.18 (s, 1H), 3.64 – 3.58 (m, 1H),

3.12 – 2.96 (m, 4H), 2.94 (d, J = 11.9 Hz, 1H), 2.74 (d, J = 11.2 Hz, 1H), 2.47 (dd, J = 17.4, 5.8 Hz, 1H), 2.37 – 2.18 (m, 3H), 2.07 (dd, J = 20.0, 11.2 Hz, 1H), 1.89 – 1.69 (m, 5H), 1.55 – 1.44 (m, 3H), 1.13 (td, J = 12.8, 4.1 Hz, 1H). ^{13}C NMR (125 MHz, $CDCl_3$) δ 169.35, 156.42, 146.88, 133.91, 132.19, 130.07, 128.80, 128.28, 128.12, 126.55, 125.92, 125.59, 123.98, 109.60, 59.58, 54.73, 51.61, 45.88, 40.03, 38.02, 29.65, 26.16, 25.55, 25.41, 24.68, 23.01, 19.19.

S1.8 (*E*)-10-chloro-2,3,4^1,6,7,7a,12,13,13a,13b-decahydro-1*H*,5*H*,8*H*-dipyrido [2,1-*f*:3',2',1'-*ij*][1,6]naphthyridine-11-carbaldehyde *O*-(3-nitrobenzoyl) oxime (**SOP-2h**, $C_{23}H_{27}ClN_4O_4$)

The compound was obtained in 78% yield as a white solid. ^{1}H NMR (500 MHz, $CDCl_3$) δ 8.92 – 8.87 (m, 1H), 8.63 (s, 1H), 8.42 (ddt, J = 7.5, 5.1, 1.2 Hz, 2H), 7.68 (t, J = 8.0 Hz, 1H), 3.62 (dd, J = 12.3, 4.6 Hz, 1H), 3.09 (d, J = 11.2 Hz, 1H), 3.00 – 2.88 (m, 2H), 2.83 (td, J = 13.3, 2.7 Hz, 1H), 2.66 (dd, J = 11.2, 5.6 Hz, 1H), 2.60 (ddd, J = 16.7, 5.8, 1.7 Hz, 1H), 2.51 – 2.42 (m, 3H), 2.21 (dtd, J = 15.3, 11.3, 5.6 Hz, 1H), 1.98 – 1.91 (m, 1H), 1.91 – 1.76 (m, 4H), 1.75 – 1.58 (m, 3H), 1.53 – 1.47 (m, 1H), 1.34 (d, J = 11.6 Hz, 1H), 1.09 (qd, J = 12.9, 4.1 Hz, 1H). ^{13}C NMR (125 MHz, $CDCl_3$) δ 162.33, 157.45, 148.35, 147.31, 135.47, 131.00, 129.87, 127.65, 124.56, 108.36, 63.14, 59.77, 54.75, 51.77, 46.14, 39.99, 29.67, 26.52, 25.30, 25.12, 24.40, 23.31, 19.46. HRMS (ESI): $C_{23}H_{28}ClN_4O_4$ (459.1794) $[M+H]^+$ = 459.1799.

S1.9 (*E*)-10-chloro-2,3,4^1,6,7,7a,12,13,13a,13b-decahydro-1*H*,5*H*,8*H*-dipyrido [2,1-*f*:3',2',1'-*ij*][1,6]naphthyridine-11-carbaldehyde *O*-(4-nitrobenzoyl) oxime (**SOP-2i**, $C_{23}H_{27}ClN_4O_4$)

The compound was obtained in 92% yield as a yellow solid. ^{1}H NMR (500 MHz, $CDCl_3$) δ 8.60 (s, 1H), 8.33 – 8.22 (m, 4H), 3.62 (dd, J = 12.3, 4.6 Hz, 1H), 3.08 (d, J = 11.3 Hz, 1H), 3.00 – 2.89 (m, 2H), 2.88 – 2.81 (m, 1H), 2.69 (dd, J = 10.5, 5.0 Hz, 1H), 2.62 – 2.54 (m, 1H), 2.52 – 2.39 (m, 3H), 2.21 (dtd, J = 15.4, 11.4, 9.4, 5.9 Hz, 1H), 1.99 – 1.92 (m, 1H), 1.90 – 1.75 (m, 4H), 1.75 – 1.58 (m, 3H), 1.50 (dt, J = 11.9, 3.1 Hz, 1H), 1.36 (d, J = 13.1 Hz, 1H), 1.09 (qd, J = 13.0, 4.2 Hz, 1H). ^{13}C NMR (125 MHz, $CDCl_3$) δ 162.57, 157.41, 150.68, 147.23, 134.68, 130.85, 123.76, 108.99, 63.19, 59.68, 54.46, 51.61, 46.01, 39.61, 29.32, 26.62, 25.13, 24.77, 24.52, 23.14, 19.27.

HRMS (ESI): $C_{23}H_{28}ClN_4O_4$ (459.1794) $[M+H]^+$ = 459.1796.

S1.10 (*E*)-10-chloro-2,3,4^1,6,7,7a,12,13,13a,13b-decahydro-1*H*,5*H*,8*H*-dipyrido[2,1-*f*:3',2',1'-*ij*][1,6]naphthyridine-11-carbaldehyde *O*-(4-fluorobenzoyl) oxime (**SOP-2j**, $C_{23}H_{27}ClFN_3O_2$)

The compound was obtained in 90% yield as a white solid. ^{1}H NMR (500 MHz, $CDCl_3$) δ 8.58 (s, 1H), 8.17 – 8.05 (m, 2H), 7.18 – 7.07 (m, 2H), 3.60 (dd, J = 12.3, 4.5 Hz, 1H), 3.06 (d, J = 11.5 Hz, 1H), 3.00 – 2.94 (m, 1H), 2.94 – 2.80 (m, 2H), 2.66 (dd, J = 11.2, 5.5 Hz, 1H), 2.64 – 2.57 (m, 1H), 2.51 – 2.42 (m, 2H), 2.39 (t, J = 11.9 Hz, 1H), 2.20 (dtt, J = 15.4, 7.3, 4.2 Hz, 1H), 1.95 – 1.81 (m, 3H), 1.81 – 1.74 (m, 2H), 1.74 – 1.55 (m, 3H), 1.52 – 1.45 (m, 1H), 1.31 (d, J = 13.9 Hz, 1H), 1.09 (qd, J = 12.7, 4.1 Hz, 1H). ^{13}C NMR (125 MHz, $CDCl_3$) δ 163.50, 156.88, 146.99, 132.32, 132.25, 115.86, 115.68, 109.37, 63.41, 59.67, 54.83, 51.74, 46.00, 40.17, 29.78, 26.25, 25.58, 25.47, 24.69, 23.19, 19.32. HRMS (ESI): $C_{23}H_{28}ClFN_3O_2$ (432.1849) $[M+H]^+$ = 432.1852.

S1.11 (*E*)-10-chloro-2,3,4^1,6,7,7a,12,13,13a,13b-decahydro-1*H*,5*H*,8*H*-dipyrido[2,1-*f*:3',2',1'-*ij*][1,6]naphthyridine-11-carbaldehyde *O*-(2-chlorobenzoyl) oxime (**SOP-2k**, $C_{23}H_{27}Cl_2N_3O_2$)

The compound was obtained in 94% yield as a white solid. ^{1}H NMR (500 MHz, $CDCl_3$) δ 8.56 (s, 1H), 7.79 (dd, J = 7.7, 1.5 Hz, 1H), 7.49 – 7.39 (m, 2H), 7.33 (td, J = 7.5, 1.5 Hz, 1H), 3.60 (dd, J = 12.3, 4.5 Hz, 1H), 3.06 (d, J = 11.5 Hz, 1H), 3.01 – 2.84 (m, 3H), 2.70 (dd, J = 10.2, 4.7 Hz, 1H), 2.64 – 2.56 (m, 1H), 2.52 – 2.43 (m, 2H), 2.40 (t, J = 11.9 Hz, 1H), 2.21 (dtd, J = 15.5, 7.4, 4.4 Hz, 1H), 1.99 – 1.92 (m, 1H), 1.90 – 1.74 (m, 4H), 1.74 – 1.59 (m, 3H), 1.53 – 1.46 (m, 1H), 1.34 (d, J = 19.1 Hz, 1H), 1.10 (qd, J = 12.6, 4.0 Hz, 1H). ^{13}C NMR (125 MHz, $CDCl_3$) δ 163.60, 156.95, 147.00, 139.70, 131.11, 128.95, 127.60, 109.36, 63.37, 59.70, 54.77, 51.70, 46.01, 40.04, 29.68, 26.33, 25.49, 25.32, 24.67, 23.17, 19.31. HRMS (ESI): $C_{23}H_{28}Cl_2N_3O_2$ (448.1553) $[M+H]^+$ = 448.1558.

S1.12 (*E*)-10-chloro-2,3,4^1,6,7,7a,12,13,13a,13b-decahydro-1*H*,5*H*,8*H*-dipyrido[2,1-*f*:3',2',1'-*ij*][1,6]naphthyridine-11-carbaldehyde *O*-(3-chlorobenzoyl) oxime (**SOP-2l**, $C_{23}H_{27}Cl_2N_3O_2$)

The compound was obtained in 80% yield as a white solid. ^{1}H NMR (500 MHz, $CDCl_3$) δ 8.56 (s, 1H), 7.79 (dd, J = 7.7, 1.5 Hz, 1H), 7.49 – 7.39 (m, 2H), 7.33 (td, J = 7.5, 1.5 Hz, 1H), 3.61 (dd, J = 12.3, 4.5 Hz, 1H), 3.06 (d, J = 11.5 Hz, 1H), 3.00 – 2.84 (m, 3H), 2.71 (dd, J = 10.2, 4.7 Hz, 1H), 2.64 – 2.56 (m, 1H), 2.52 – 2.42 (m, 2H), 2.40 (t, J = 11.9 Hz, 1H), 2.21 (dtt, J = 15.5, 7.4, 4.3 Hz, 1H), 1.99 – 1.92 (m, 1H), 1.91 – 1.74 (m, 4H), 1.74 – 1.60 (m, 3H), 1.53 – 1.46 (m, 1H), 1.34 (d, J = 19.1 Hz, 1H), 1.10 (qd, J = 12.6, 4.0 Hz, 1H). ^{13}C NMR (125 MHz, $CDCl_3$) δ 163.22, 157.01, 146.99, 134.73, 133.30, 130.89, 129.94, 129.72, 127.89, 109.67, 63.32, 59.63, 54.47, 51.55, 45.90, 39.67, 29.34, 26.47, 25.26, 24.93, 24.69, 23.03, 19.17. HRMS (ESI): $C_{23}H_{28}Cl_2N_3O_2$ (448.1553) $[M+H]^+$ = 448.1556.

S1.13 (*E*)-10-chloro-2,3,4^1,6,7,7a,12,13,13a,13b-decahydro-1*H*,5*H*,8*H*-dipyrido[2,1-*f*:3',2',1'-*ij*][1,6]naphthyridine-11-carbaldehyde *O*-(4-chlorobenzoyl) oxime (**SOP-2m**, $C_{23}H_{27}Cl_2N_3O_2$)

The compound was obtained in 80% yield as a white solid. ^{1}H NMR (500 MHz, $CDCl_3$) δ 8.58 (s, 1H), 8.02 (d, J = 8.6 Hz, 2H), 7.43 (d, J = 8.6 Hz, 2H), 3.61 (dd, J = 12.3, 4.5 Hz, 1H), 3.06 (d, J = 11.5 Hz, 1H), 3.01 – 2.95 (m, 1H), 2.95 – 2.82 (m, 2H), 2.68 (dd, J = 11.2, 5.5 Hz, 1H), 2.65 – 2.57 (m, 1H), 2.46 (ddd, J = 17.5, 11.0, 7.3 Hz, 2H), 2.40 (t, J = 11.9 Hz, 1H), 2.22 (ddt, J = 11.4, 8.1, 4.1 Hz, 1H), 1.98 – 1.91 (m, 1H), 1.91 – 1.74 (m, 4H), 1.73 – 1.58 (m, 3H), 1.50 (dd, J = 12.3, 3.2 Hz, 1H), 1.34 (d, J = 12.0 Hz, 1H), 1.09 (dd, J = 12.0, 4.2 Hz, 1H). ^{13}C NMR (125 MHz, $CDCl_3$) δ 163.60, 156.95, 147.00, 139.70, 131.11, 128.95, 127.60, 109.36, 63.37, 59.70, 54.77, 51.70, 46.01, 40.04, 29.68, 26.33, 25.49, 25.32, 24.67, 23.17, 19.31. HRMS (ESI): $C_{23}H_{28}Cl_2N_3O_2$ (448.1553) $[M+H]^+$ = 448.1559.

S1.14 (*E*)-10-chloro-2,3,4^1,6,7,7a,12,13,13a,13b-decahydro-1*H*,5*H*,8*H*-dipyrido[2,1-*f*:3',2',1'-*ij*][1,6]naphthyridine-11-carbaldehyde *O*-cyclohexanecarbonyl oxime (**SOP-2n**, $C_{23}H_{34}ClN_3O_2$)

The compound was obtained in 76% yield as a white solid. ^{1}H NMR (500 MHz, $CDCl_3$) δ 8.40 (s, 1H), 3.59 (dd, J = 12.1, 4.3 Hz, 1H), 3.01 (d, J = 11.7 Hz, 1H), 2.99 – 2.87 (m, 3H), 2.76 (dd, J = 11.2, 5.2 Hz, 1H), 2.53 (dd, J = 16.9, 5.4 Hz, 2H), 2.44 – 2.35 (m, 3H), 2.32 (t, J = 11.8 Hz, 1H), 2.21 (dtt, J = 19.5, 11.8, 4.1 Hz, 1H), 2.03 –

1.95 (m, 1H), 1.91 – 1.72 (m, 5H), 1.67 (tt, *J* = 15.0, 8.2 Hz, 4H), 1.53 – 1.47 (m, 1H), 1.42 – 1.15 (m, 5H), 1.09 (qd, *J* = 12.7, 4.1 Hz, 1H), 0.86 (t, *J* = 7.0 Hz, 3H). ^{13}C NMR (125 MHz, $CDCl_3$) δ 171.74, 155.97, 146.62, 110.35, 77.41, 63.46, 59.55, 54.52, 51.46, 45.76, 39.74, 33.05, 31.90, 29.37, 29.30, 29.25, 29.21, 26.23, 25.43, 25.14, 25.03, 24.82, 22.85, 22.75, 19.02, 14.21. HRMS (ESI): $C_{23}H_{35}ClN_3O_2$ (420.2412) $[M+H]^+$ = 420.2406.

S1.15 (*E*)-10-chloro-2,3,4^1,6,7,7a,12,13,13a,13b-decahydro-1*H*,5*H*,8*H*-dipyri- do[2,1-*f*:3',2',1'-*ij*][1,6]naphthyridine-11-carbaldehyde *O*-propionyl oxime (**SOP-2o**, $C_{19}H_{28}ClN_3O_2$)

The compound was obtained in 70% yield as a white solid. ^{1}H NMR (500 MHz, $CDCl_3$) δ 8.38 (s, 1H), 3.63 (dd, *J* = 11.8, 3.8 Hz, 1H), 3.11 – 2.95 (m, 5H), 2.74 (d, *J* = 11.2 Hz, 1H), 2.53 (dd, *J* = 17.3, 5.8 Hz, 1H), 2.45 – 2.15 (m, 6H), 1.96 – 1.72 (m, 6H), 1.65 (dd, *J* = 13.3, 7.7 Hz, 1H), 1.58 – 1.47 (m, 2H), 1.19 (t, *J* = 7.6 Hz, 3H), 1.13 (td, *J* = 13.8, 12.7, 5.5 Hz, 1H). ^{13}C NMR (125 MHz, $CDCl_3$) δ 172.41, 155.67, 146.26, 111.43, 63.32, 59.42, 53.78, 50.99, 45.47, 38.74, 28.43, 26.54, 26.36, 24.92, 24.80, 24.11, 22.38, 18.60, 9.16. HRMS (ESI): $C_{19}H_{29}ClN_3O_2$ (366.1943) $[M+H]^+$ = 366.1939.

S1.16 (*E*)-10-chloro-2,3,4^1,6,7,7a,12,13,13a,13b-decahydro-1*H*,5*H*,8*H*-dipyri- do[2,1-*f*:3',2',1'-*ij*][1,6]naphthyridine-11-carbaldehyde *O*-butyryl oxime **(SOP-2p**, $C_{20}H_{30}ClN_3O_2$)

The compound was obtained in 80% yield as a yellow solid. ^{1}H NMR (500 MHz, $CDCl_3$) δ 8.37 (s, 1H), 3.68 (d, *J* = 8.0 Hz, 1H), 3.31 – 3.10 (m, 3H), 3.11 – 3.05 (m, 2H), 3.06 – 2.92 (m, 2H), 2.55 (dd, *J* = 17.4, 6.1 Hz, 1H), 2.49 – 2.29 (m, 5H), 1.93 – 1.78 (m, 4H), 1.70 (h, *J* = 7.4 Hz, 4H), 1.62 (q, *J* = 7.4 Hz,1H), 1.37 (t, *J* = 7.3 Hz, 2H), 1.27 – 1.14 (m, 1H), 0.96 (t, *J* = 7.4 Hz, 3H). ^{13}C NMR (125 MHz, $CDCl_3$) δ 171.42, 155.43, 145.90, 112.66, 63.22, 59.18, 52.91, 50.51, 45.78, 37.64, 34.79, 27.43, 26.88, 25.06, 24.15, 22.99, 21.94, 18.46, 13.77, 8.74. HRMS (ESI): $C_{20}H_{31}ClN_3O_2$ (380.2099) $[M+H]^+$ = 380.2096.

S1.17 (*E*)-10-chloro-2,3,4^1,6,7,7a,12,13,13a,13b-decahydro-1*H*,5*H*,8*H*-dipyri- do[2,1-*f*:3',2',1'-*ij*][1,6]naphthyridine-11-carbaldehyde *O*-nonanoyl oxime (**SOP-2q**, $C_{25}H_{40}ClN_3O_2$)

The compound was obtained in 90% yield as a yellow solid. ^{1}H NMR (500 MHz, $CDCl_3$) δ 8.40 (s, 1H), 3.58 (dd, J = 12.2, 4.3 Hz, 1H), 3.01 (d, J = 11.7 Hz, 1H), 2.99 – 2.84 (m, 3H), 2.70 (dd, J = 11.1, 5.2 Hz, 1H), 2.51 (ddd, J = 17.5, 14.3, 4.3 Hz, 2H), 2.43 – 2.35 (m, 3H), 2.32 (t, J = 11.8 Hz, 1H), 2.25 – 2.15 (m, 1H), 1.96 – 1.90 (m, 1H), 1.89 – 1.74 (m, 4H), 1.67 (qt, J = 13.1, 5.6 Hz, 5H), 1.50 – 1.44 (m, 1H), 1.36 – 1.23 (m, 11H), 1.08 (qd, J = 12.8, 4.2 Hz, 1H), 0.86 (t, J = 6.9 Hz, 3H). ^{13}C NMR (125 MHz, $CDCl_3$) δ 171.73, 155.97, 146.62, 110.28, 63.45, 59.55, 54.55, 51.47, 45.78, 39.79, 33.04, 31.89, 29.41, 29.29, 29.24, 29.21, 26.21, 25.45, 25.19, 25.03, 24.81, 22.86, 22.74, 19.04, 14.20. HRMS (ESI): $C_{25}H_{41}ClN_3O_2$ (450.2882) [M+H]$^+$ = 450.2886.

S1.18 (*E*)-10-chloro-2,3,4^{1},6,7,7a,12,13,13a,13b-decahydro-1*H*,5*H*,8*H*-dipyri- do[2,1-*f*:3',2',1'-*ij*][1,6]naphthyridine-11-carbaldehyde *O*-decanoyl oxime (**SOP-2r**, $C_{26}H_{42}ClN_3O_2$)

The compound was obtained in 80% yield as a white solid. ^{1}H NMR (500 MHz, $CDCl_3$) δ 8.40 (s, 1H), 3.58 (dd, J = 12.2, 4.3 Hz, 1H), 3.05 – 2.99 (m, 1H), 2.99 – 2.87 (m, 5H), 2.74 (dd, J = 11.2, 5.3 Hz, 1H), 2.57 – 2.50 (m, 3H), 2.43 – 2.35 (m, 3H), 2.31 (d, J = 11.9 Hz, 1H), 2.27 – 2.15 (m, 2H), 2.01 – 1.94 (m, 2H), 1.89 – 1.74 (m, 6H), 1.74 – 1.60 (m, 6H), 1.49 (dd, J = 8.4, 3.8 Hz, 1H), 1.39 – 1.18 (m, 5H), 1.09 (dd, J = 12.1, 4.3 Hz, 1H), 0.86 (t, J = 7.0 Hz, 3H). ^{13}C NMR (125 MHz, $CDCl_3$) δ 171.73, 155.97, 146.62, 110.28, 63.45, 59.55, 54.55, 51.47, 45.78, 39.79, 33.04, 31.89, 29.41, 29.29, 29.24, 29.21, 26.21, 25.45, 25.19, 25.03, 24.81, 22.86, 22.74, 19.04, 14.21. HRMS (ESI): $C_{26}H_{43}ClN_3O_2$ (464.3038) [M+H]$^+$ = 464.3034.

S1.19 (4^{1}S,7a*R*,13a*R*,13b*R*)-11-((*E*)-((benzoyloxy)imino)methyl)-10-chloro-2,3, 6,7,7a,8,12,13,13a,13b-decahydro-1*H*,5*H*-4λ^{4}-dipyrido[2,1-*f*:3',2',1'-*ij*][1,6]naphthyridin-4(4^{1}*H*)-olate (**SOP-3a**, $C_{23}H_{28}ClN_3O_3$)

The compound was obtained in 54% yield as a yellow solid. ^{1}H NMR (500 MHz, $CDCl_3$) δ 8.51 (s, 1H), 8.01 (d, J = 6.4 Hz, 2H), 7.55 – 7.48 (m, 1H), 7.39 (t, J = 6.8 Hz, 2H), 5.30 – 5.10 (m, 1H), 3.66 (d, J = 8.4 Hz, 1H), 3.51 (t, J = 12.3 Hz, 2H), 3.37 (dt, J = 21.7, 5.4 Hz, 2H), 3.07 (dd, J = 16.8, 11.4 Hz, 2H), 2.89 (dd, J = 39.8, 9.5 Hz, 2H), 2.58 – 2.37 (m, 3H), 2.27 (q, J = 18.0, 15.2 Hz, 2H), 1.87 – 1.42 (m, 5H), 1.31 – 1.10 (m, 2H). ^{13}C NMR (125 MHz, $CDCl_3$) δ 164.23, 156.26, 146.12, 133.39, 128.61,

127.92, 113.07, 73.38, 70.27, 63.95, 56.95, 53.57, 50.56, 33.92, 31.74, 26.79, 25.28, 23.67, 22.12, 21.77, 19.85.

S1.20 (4^1S,7a*R*,13a*R*,13b*R*)-10-chloro-11-((*E*)-((cinnamoyloxy)imino)methyl)-2,3,6,7,7a,8,12,13,13a,13b-decahydro-1*H*,5*H*-4λ^4-dipyrido[2,1-*f*:3',2',1'-ij][1,6]naphthyridin-4(4^1H)-olate (**SOP-3b**, $C_{25}H_{30}ClN_3O_3$)

The compound was obtained in 56% yield as a yellow solid. ^1H NMR (500 MHz, $CDCl_3$) δ 8.48 (s, 1H), 7.79 (d, J = 16.0 Hz, 1H), 7.53 (dd, J = 6.4, 3.0 Hz, 2H), 7.44 – 7.30 (m, 3H), 6.49 (d, J = 16.0 Hz, 1H), 3.71 (d, J = 8.3 Hz, 1H), 3.61 – 3.51 (m, 2H), 3.48 – 3.37 (m, 1H), 3.16 – 3.05 (m, 2H), 3.05 – 2.96 (m, 1H), 2.86 (d, J = 9.4 Hz, 1H), 2.57 (qd, J = 15.5, 13.6, 7.7 Hz, 2H), 2.44 (ddd, J = 17.6, 11.3, 8.0 Hz, 1H), 2.35 – 2.25 (m, 2H), 1.91 – 1.76 (m, 4H), 1.75 – 1.60 (m, 2H), 1.54 (d, J = 13.1 Hz, 1H), 1.33 – 1.15 (m, 2H). ^{13}C NMR (125 MHz, $CDCl_3$) δ 164.89, 155.80, 146.08, 134.34, 130.67, 129.02, 128.28, 115.67, 112.93, 73.87, 70.93, 64.04, 57.25, 50.60, 33.86, 31.75, 27.00, 25.25, 23.81, 22.15, 21.77, 19.86. HRMS (ESI): $C_{25}H_{31}ClN_3O_3$ (456.2048) $[M+H]^+$ = 456.2041.

S1.21 (4^1S,7a*R*,13a*R*,13b*R*)-10-chloro-11-((*E*)-(((3-chlorobenzoyl)oxy)imino) methyl)-2,3,6,7,7a,8,12,13,13a,13b-decahydro-1*H*,5*H*-4λ^4-dipyrido[2,1-*f*:3',2',1'-ij][1,6]naphthyridin-4(4^1H)-olate (**SOP-3c**, $C_{23}H_{27}Cl_2N_3O_3$)

The compound was obtained in 80% yield as a white solid. ^1H NMR (500 MHz, $CDCl_3$) δ 8.57 (s, 1H), 8.04 (s, 1H), 7.96 (d, J = 7.8 Hz, 1H), 7.58 – 7.50 (m, 1H), 7.40 (t, J = 7.9 Hz, 1H), 5.29 (s, 1H), 3.80 – 3.65 (m, 2H), 3.65 – 3.53 (m, 2H), 3.32 (dd, J = 10.9, 3.9 Hz, 1H), 3.12 (d, J = 12.1 Hz, 1H), 3.06 (d, J = 9.9 Hz, 1H), 3.03 – 2.96 (m, 1H), 2.62 (dd, J = 17.4, 6.2 Hz, 1H), 2.58 – 2.42 (m, 2H), 2.39 – 2.27 (m, 2H), 1.95 – 1.78 (m, 4H), 1.75 – 1.67 (m, 2H), 1.58 (d, J = 13.2 Hz, 1H), 1.37 – 1.19 (m, 1H). ^{13}C NMR (125 MHz, $CDCl_3$) δ 163.03, 156.59, 146.44, 134.75, 133.41, 130.67, 129.97, 129.71, 127.91, 112.76, 73.40, 70.36, 63.98, 57.00, 50.59, 33.91, 31.71, 26.81, 25.24, 23.69, 22.12, 21.84, 19.86. HRMS (ESI): $C_{23}H_{28}Cl_2N_3O_3$ (464.1502) $[M+H]^+$ = 464.1502.

S1.22 (4^1S,7aR,13aR,13bR)-10-chloro-11-((E)-(((4-fluorobenzoyl)oxy)imino) methyl)-2,3,6,7,7a,8,12,13,13a,13b-decahydro-1H,5H-4λ^4-dipyrido[2,1-f:3',2',1'-ij][1,6]naphthyridin-4(4^1H)-olate (**SOP-3d**, $C_{23}H_{27}ClFN_3O_3$)

The compound was obtained in 80% yield as a white solid. ^{1}H NMR (500 MHz, $CDCl_3$) δ 8.54 (s, 1H), 8.08 (d, J = 13.0 Hz, 2H), 7.11 (t, J = 8.2 Hz, 2H), 3.72 (d, J = 8.9 Hz, 1H), 3.57 (t, J = 12.7 Hz, 2H), 3.44 (dd, J = 19.0, 10.6 Hz, 1H), 3.11 (t, J = 10.3 Hz, 1H), 3.01 (d, J = 13.1 Hz, 1H), 2.89 (d, J = 10.7 Hz, 1H), 2.61 (dd, J = 17.5, 5.9 Hz, 1H), 2.57 – 2.39 (m, 2H), 2.39 – 2.24 (m, 2H), 1.93 – 1.77 (m, 4H), 1.75 – 1.60 (m, 2H), 1.55 (d, J = 13.0 Hz, 1H), 1.26 (dd, J = 24.0, 15.6 Hz, 3H). ^{13}C NMR (125 MHz, $CDCl_3$) δ 163.25, 156.36, 146.30, 132.33, 132.26, 125.11, 125.09, 115.88, 115.71, 112.74, 73.78, 70.84, 64.05, 57.20, 50.61, 33.87, 31.72, 26.97, 25.24, 23.80, 22.14, 21.81, 19.86. HRMS (ESI): $C_{23}H_{28}ClFN_3O_3$ (448.1798) $[M+H]^+$ = 448.1794.

S1.23 (4^1S,7aR,13aR,13bR)-10-chloro-11-((E)-(((cyclohexanecarbonyl)oxy)im-ino)methyl)-2,3,6,7,7a,8,12,13,13a,13b-decahydro-1H,5H-4λ^4-dipyrido[2,1-f:3',2',1'-ij][1,6]naphthyridin-4(4^1H)-olate (**SOP-3e**, $C_{23}H_{34}ClN_3O_3$)

The compound was obtained in 80% yield as a yellow solid. ^{1}H NMR (500 MHz, $CDCl_3$) δ 8.35 (s, 1H), 3.67 (q, J = 10.5 Hz, 2H), 3.58 – 3.47 (m, 2H), 3.46 – 3.36 (m, 1H), 3.11 (dd, J = 10.9, 4.1 Hz, 1H), 3.05 (d, J = 12.1 Hz, 1H), 2.98 – 2.92 (m, 1H), 2.88 (d, J = 9.5 Hz, 1H), 2.59 – 2.44 (m, 1H), 2.37 (ddt, J = 13.5, 10.1, 5.2 Hz, 2H), 2.28 (q, J = 10.0, 9.2 Hz, 2H), 1.95 – 1.81 (m, 4H), 1.77 (ddd, J = 23.0, 14.5, 8.4 Hz, 4H), 1.70 – 1.56 (m, 3H), 1.55 – 1.41 (m, 3H), 1.23 (td, J = 21.2, 18.5, 11.4 Hz, 5H). ^{13}C NMR (125 MHz, $CDCl_3$) δ 171.74, 155.97, 146.62, 110.35, 63.46, 59.55, 54.52, 51.46, 45.76, 39.74, 33.05, 31.90, 29.37, 29.30, 29.25, 29.21, 26.23, 25.43, 25.03, 24.82, 22.75, 19.02, 14.21.

S1.24 (4^1S,7aR,13aR,13bR)-10-chloro-11-((E)-((nonanoyloxy)imino)methyl)-2, 3,6,7,7a,8,12,13,13a,13b-decahydro-1H,5H-4λ^4-dipyrido[2,1-f:3',2',1'-ij][1,6]naphthyridin-4(4^1H)-olate (**SOP-3f**, $C_{25}H_{40}ClN_3O_3$)

The compound was obtained in 85% yield as a white solid. ^{1}H NMR (500 MHz, $CDCl_3$) δ 8.37 (s, 1H), 3.69 (dd, J = 17.4, 11.0 Hz, 2H), 3.56 (td, J = 19.5, 16.6, 8.4 Hz, 2H), 3.28 (dd, J = 10.5, 4.1 Hz, 1H), 3.08 (d, J = 12.1 Hz, 1H), 3.02 (d, J = 11.2 Hz,

1H), 2.97 (dt, J = 13.1, 4.4 Hz, 1H), 2.54 (dd, J = 17.3, 6.0 Hz, 2H), 2.44 – 2.28 (m, 5H), 1.92 – 1.76 (m, 5H), 1.66 (dt, J = 15.3, 7.8 Hz, 4H), 1.56 (t, J = 12.5 Hz, 1H), 1.31 – 1.18 (m, 11H), 0.85 (t, J = 6.9 Hz, 3H). ^{13}C NMR (125 MHz, $CDCl_3$) δ 171.64, 155.47, 145.94, 113.02, 73.42, 70.38, 63.94, 56.98, 50.54, 33.88, 32.96, 31.88, 31.73, 29.28, 29.21, 29.19, 26.84, 25.21, 24.96, 23.70, 22.73, 22.12, 21.69, 19.85, 14.20. HRMS (ESI): $C_{25}H_{41}ClN_3O_3$ (466.2831) $[M+H]^+$ = 466.2831.

S2. The ^{1}H and ^{13}C NMR and HRESIMS spectra of the derivatives

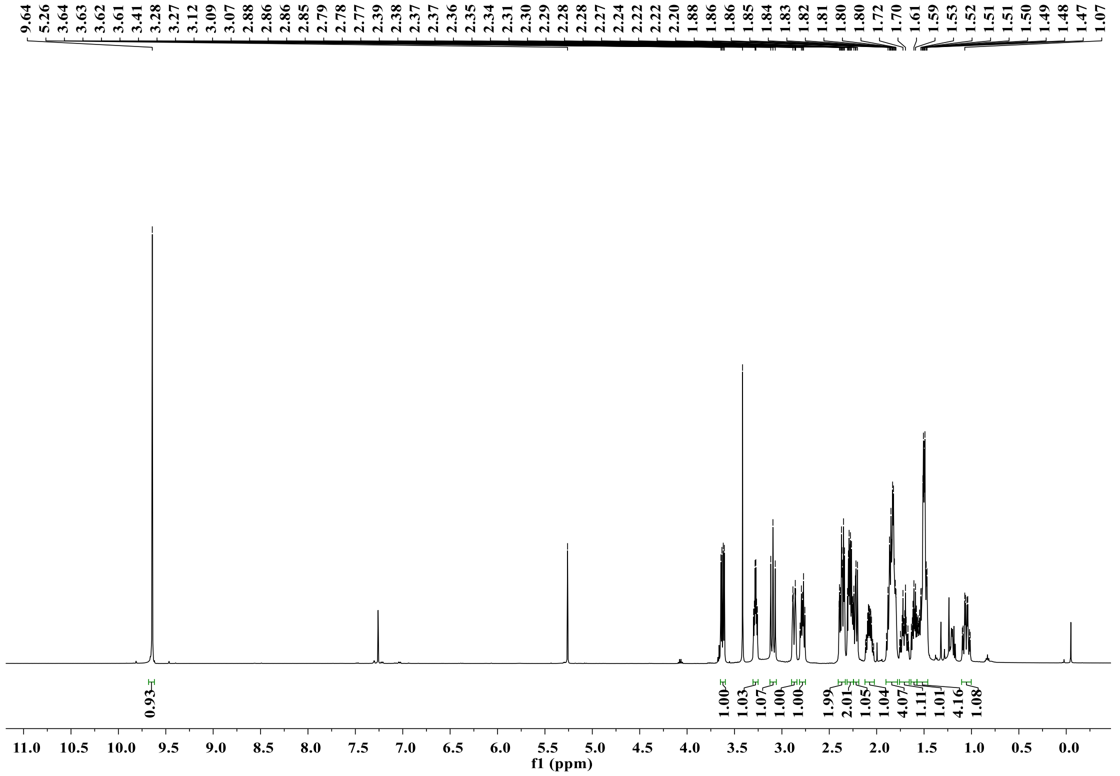

Figure S1. ^{1}H NMR spectrum of **SOP-1**

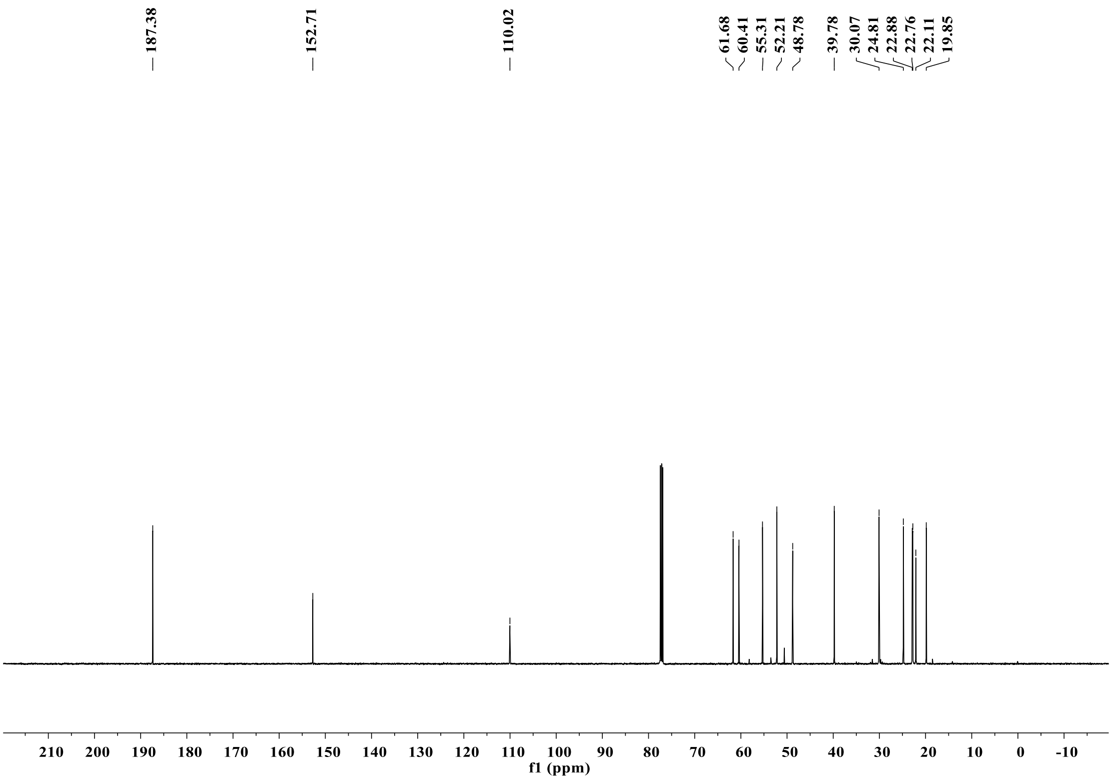

Figure S2. ^{13}C NMR spectrum of **SOP-1**

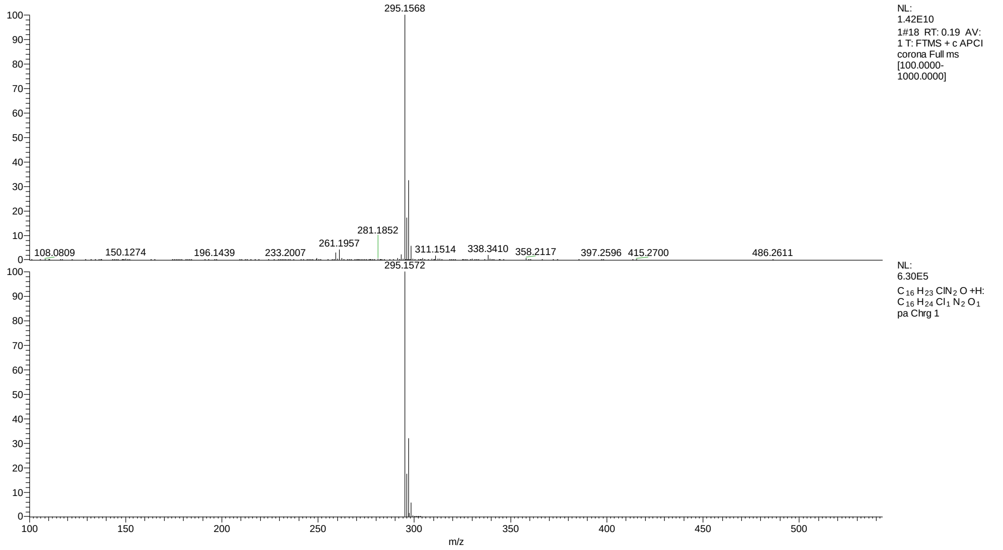

Figure S3. HR-ESI-MS spectrum of **SOP-1**

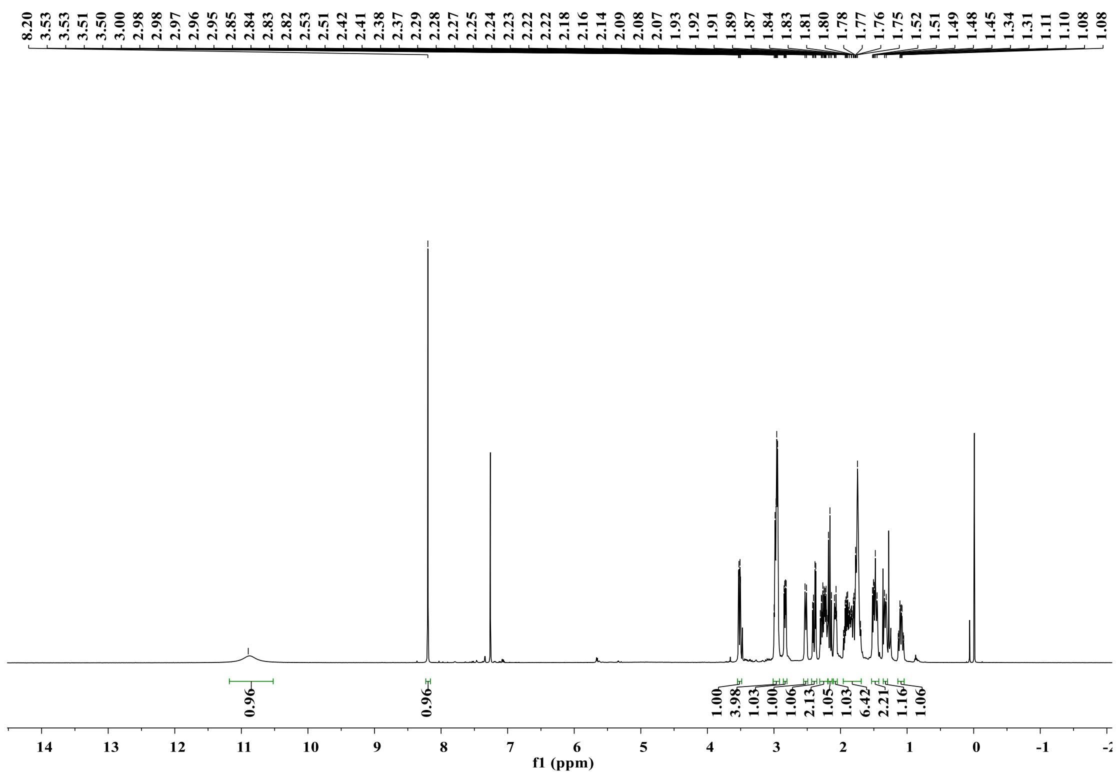

Figure S4. ^{1}H NMR spectrum of **SOP-2**

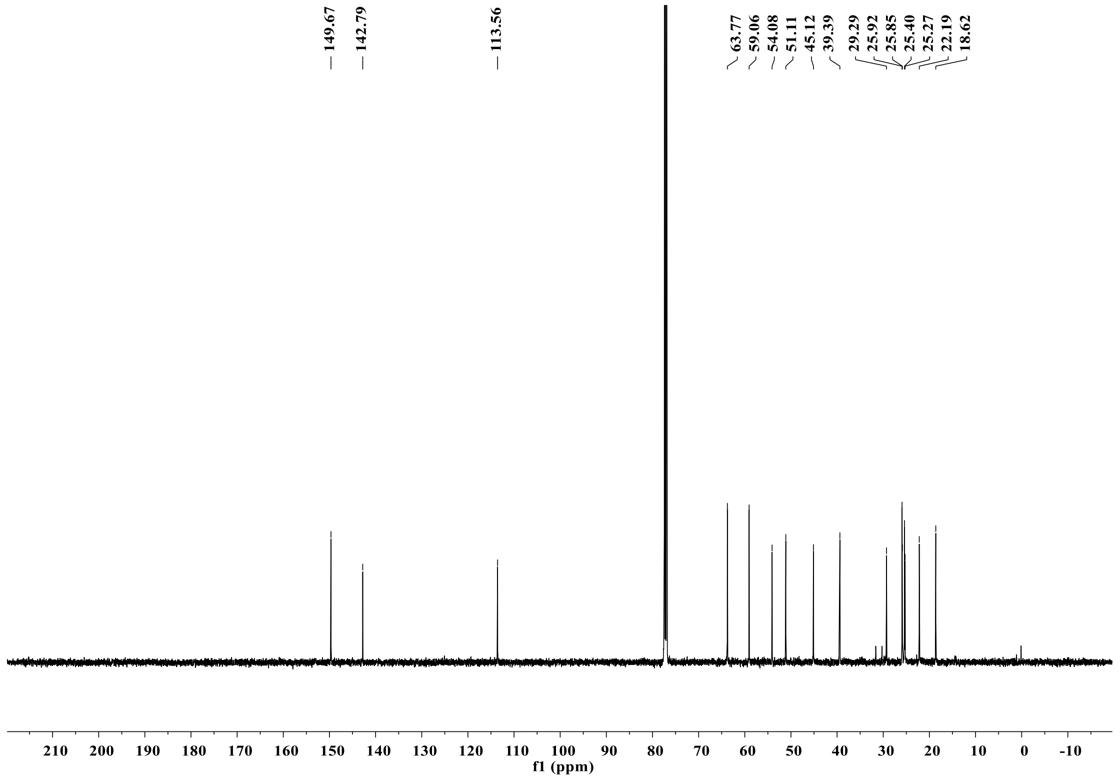

Figure S5. ^{13}C NMR spectrum of **SOP-2**

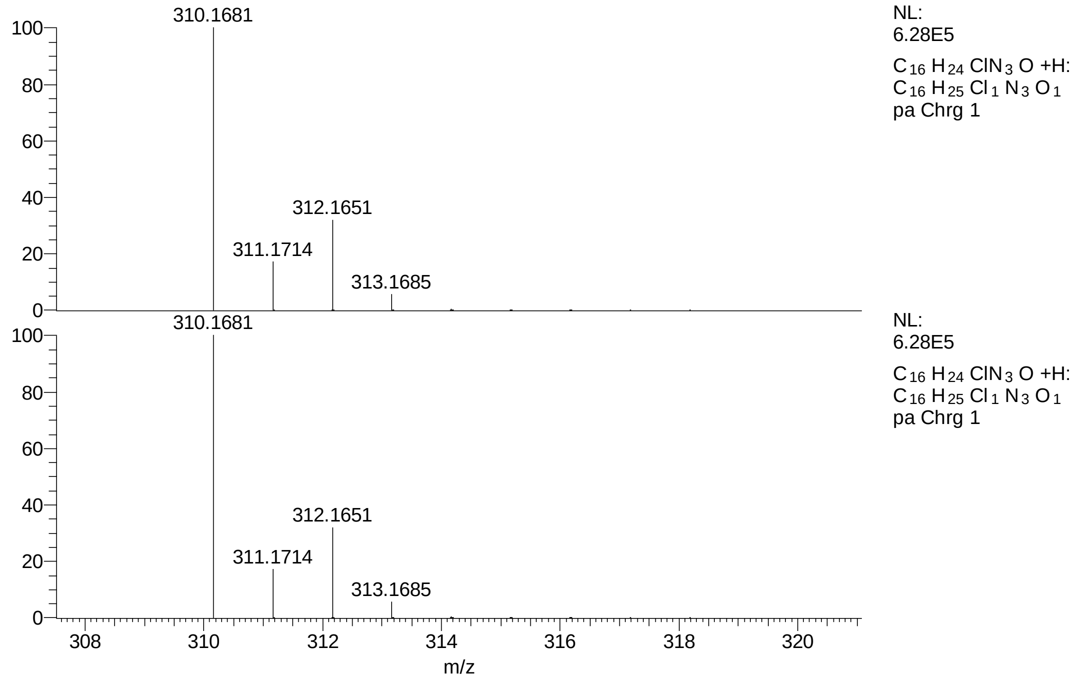

Figure S6. HR-ESI-MS spectrum of **SOP-2**

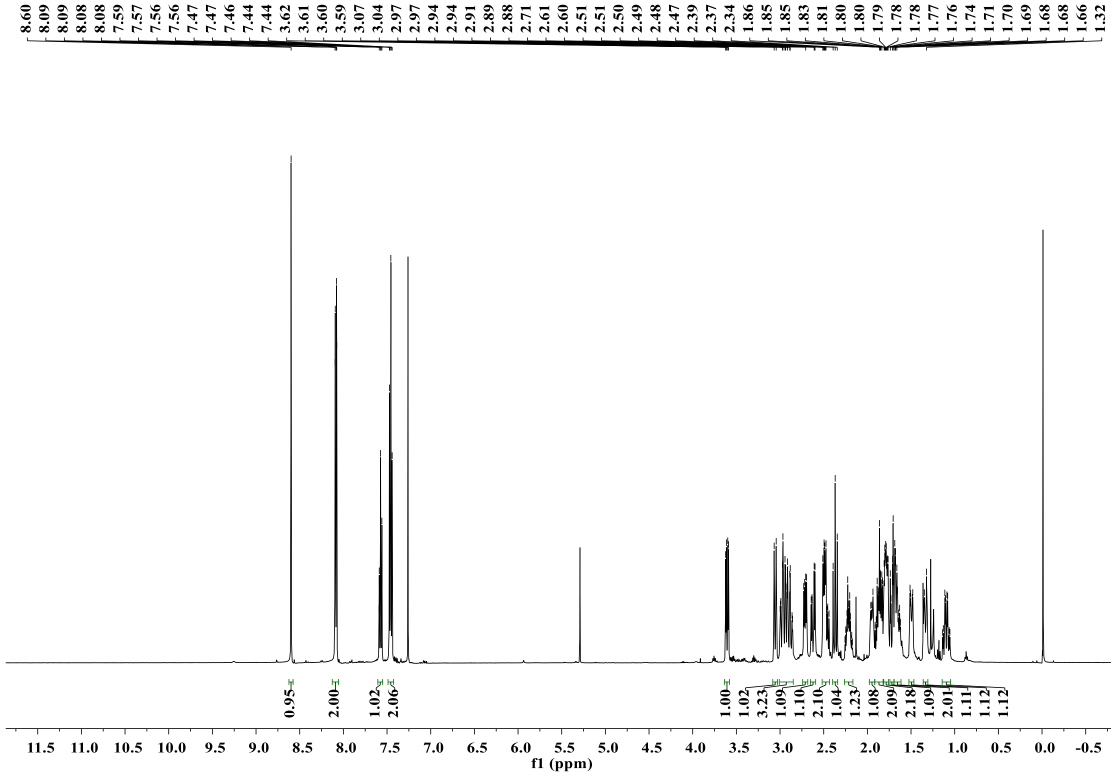

Figure S7. ^{1}H NMR spectrum of **SOP-2a**

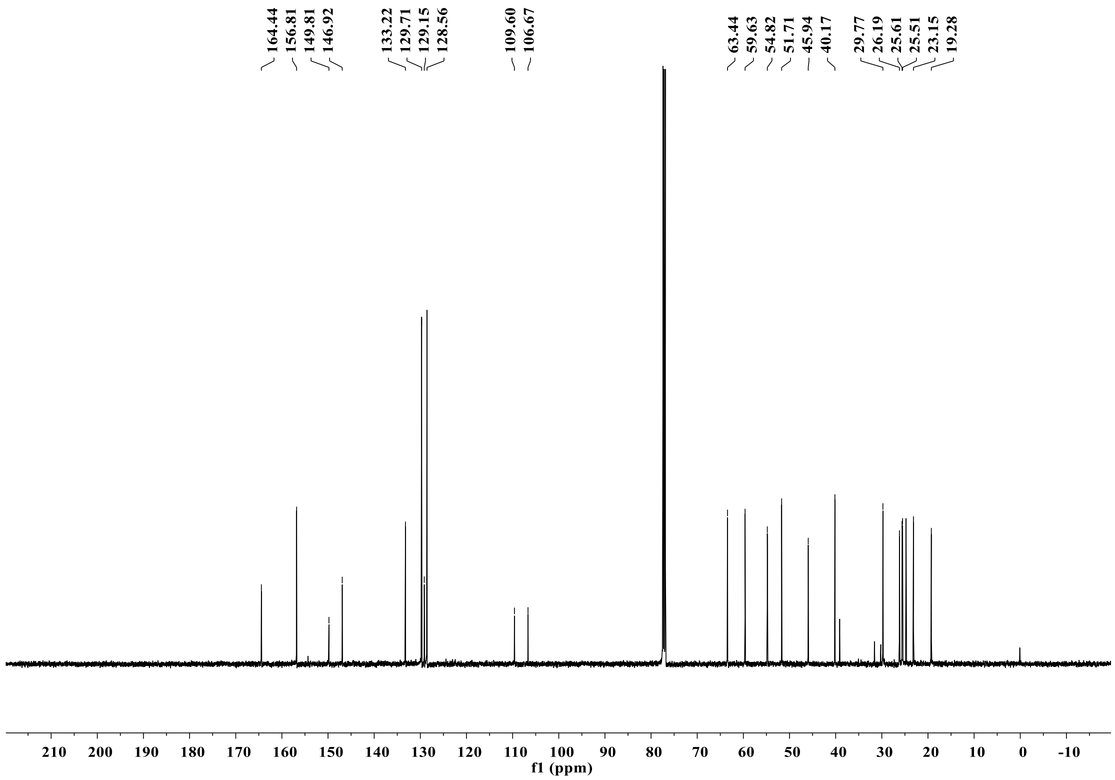

Figure S8. ^{13}C NMR spectrum of **SOP-2a**

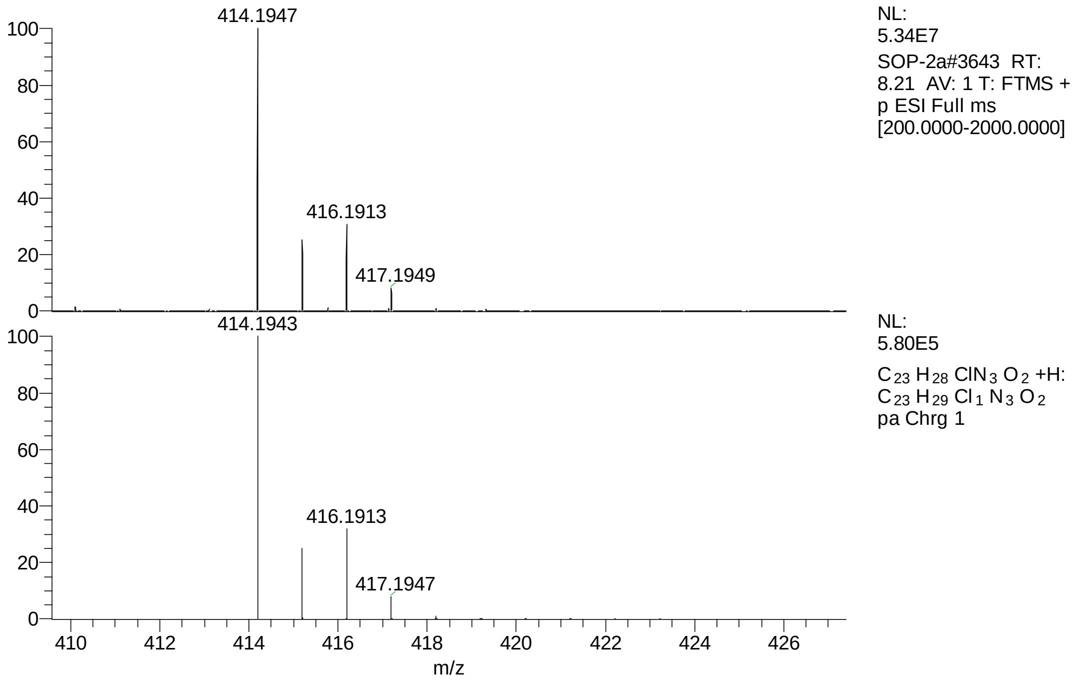

Figure S9. HR-ESI-MS spectrum of **SOP-2a**

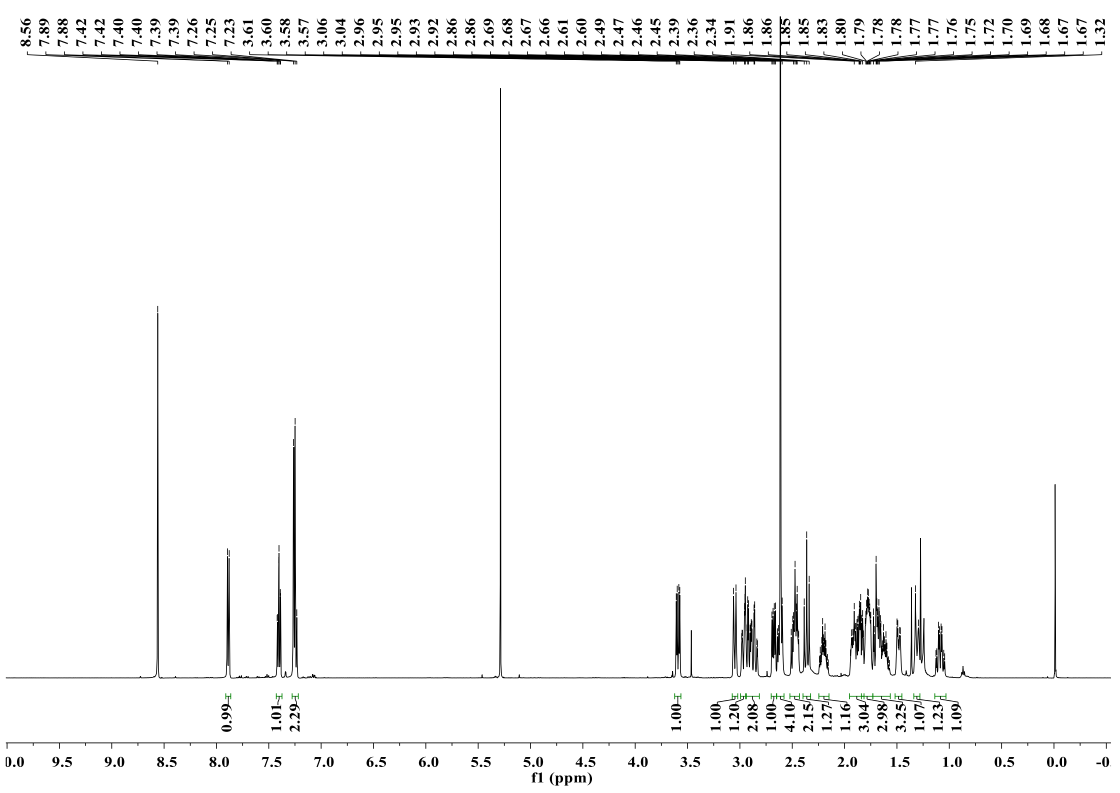

Figure S10. ^{1}H NMR spectrum of **SOP-2b**

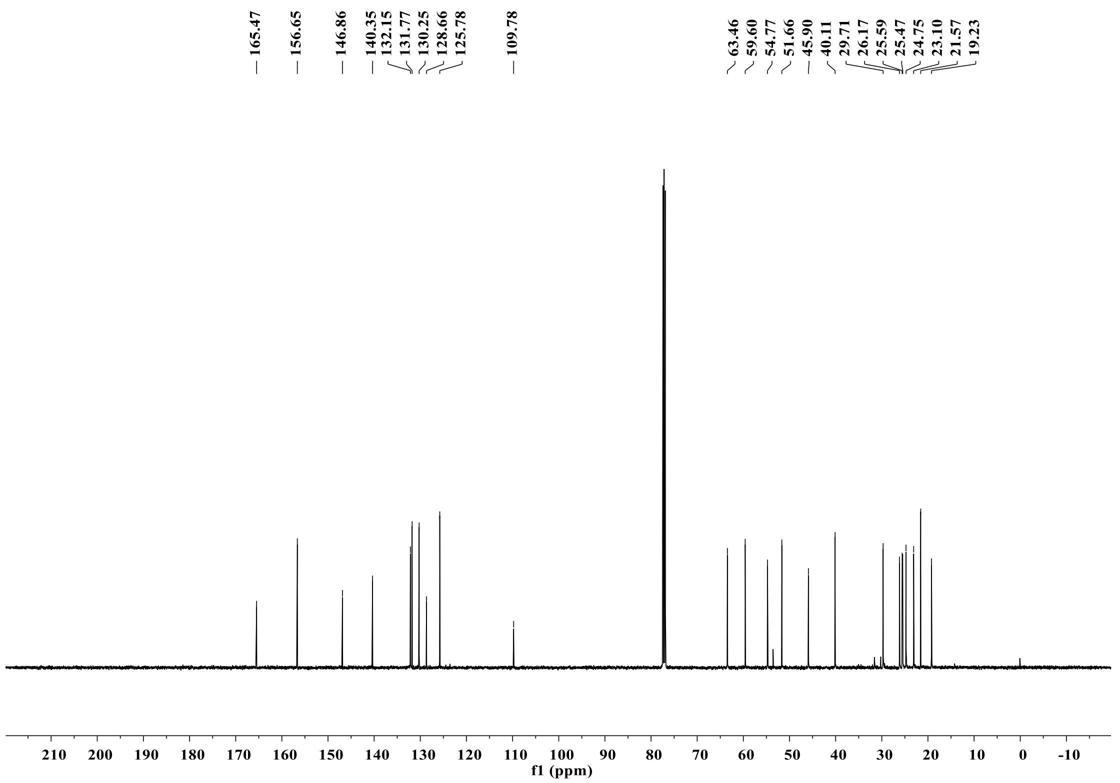

Figure S11. ^{13}C NMR spectrum of **SOP-2b**

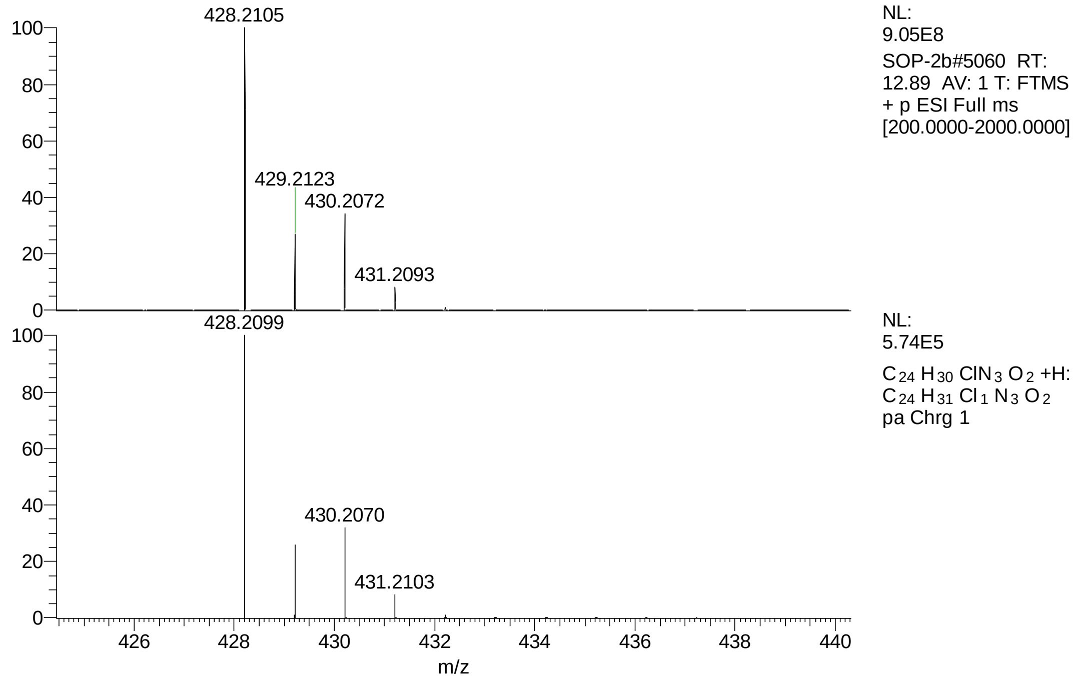

Figure S12. HR-ESI-MS spectrum of **SOP-2b**

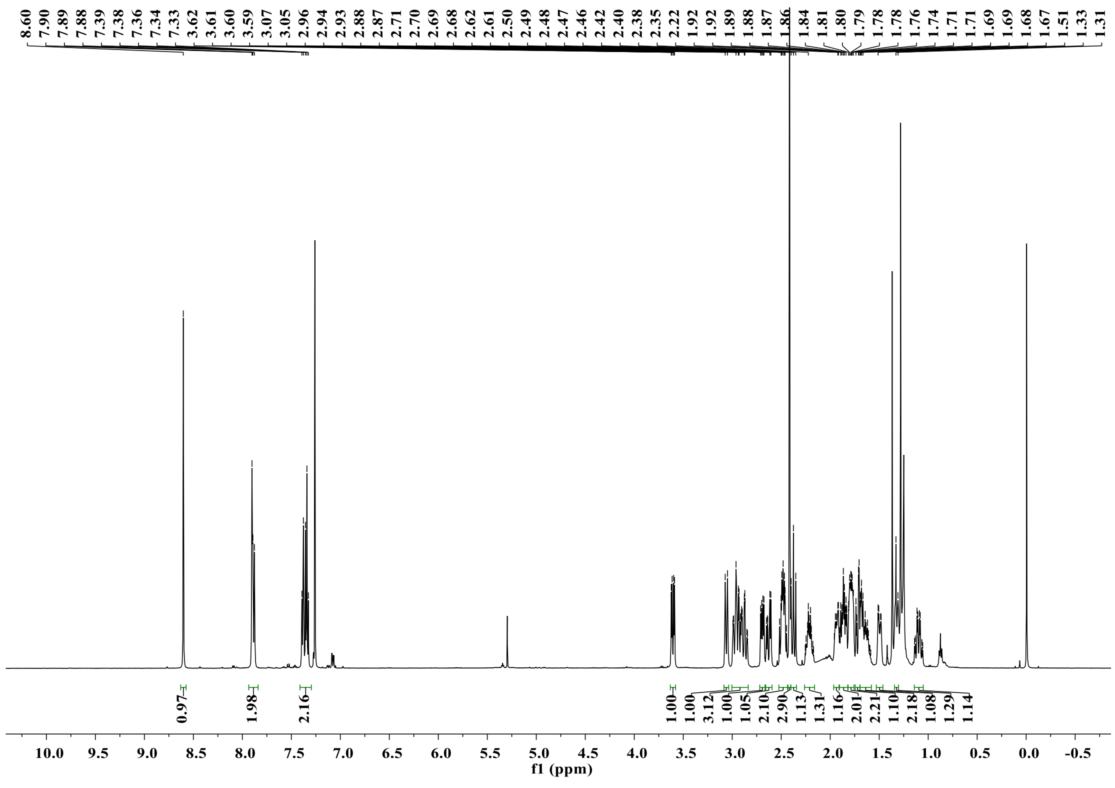

Figure S13. ^{1}H NMR spectrum of **SOP-2c**

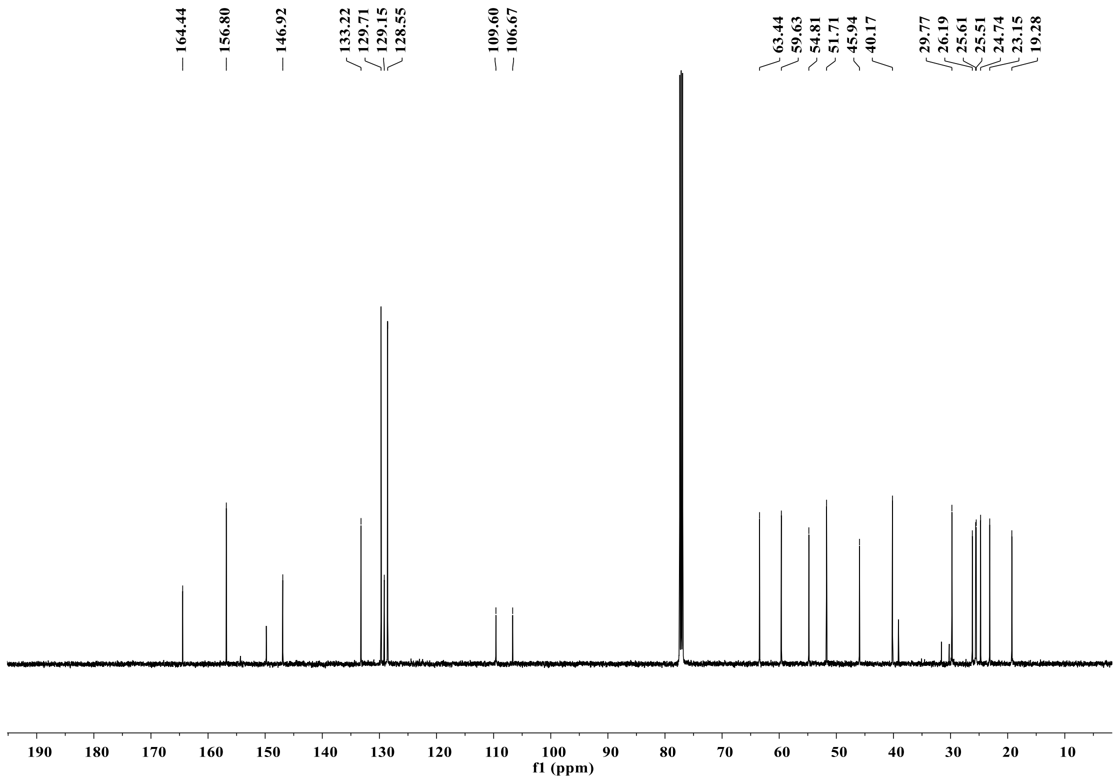

Figure S14. ^{13}C NMR spectrum of **SOP-2c**

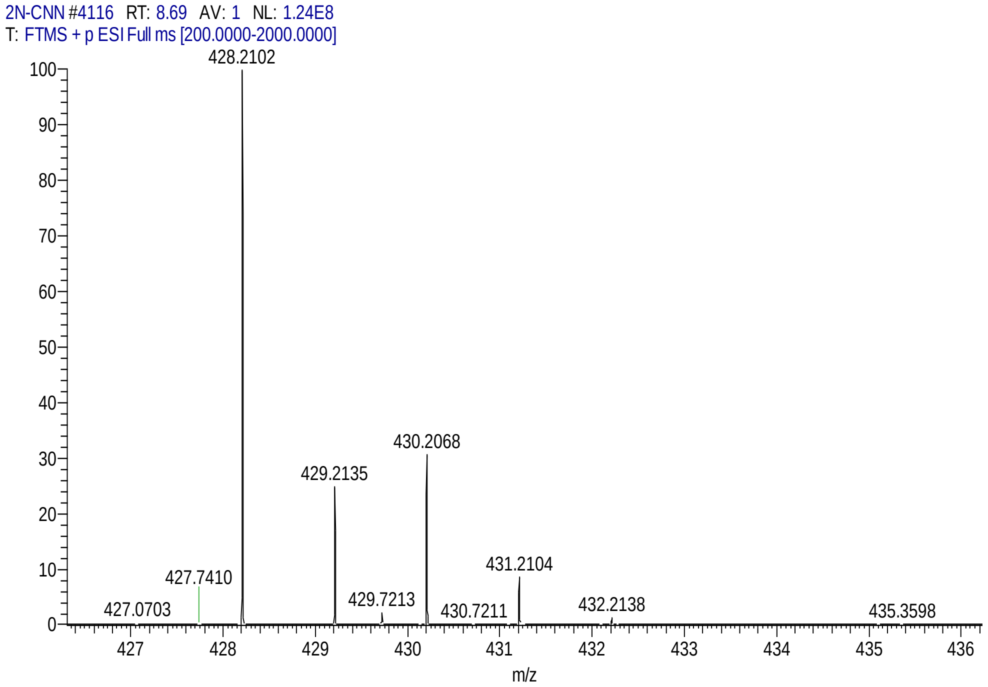

Figure S15. HR-ESI-MS spectrum of **SOP-2c**

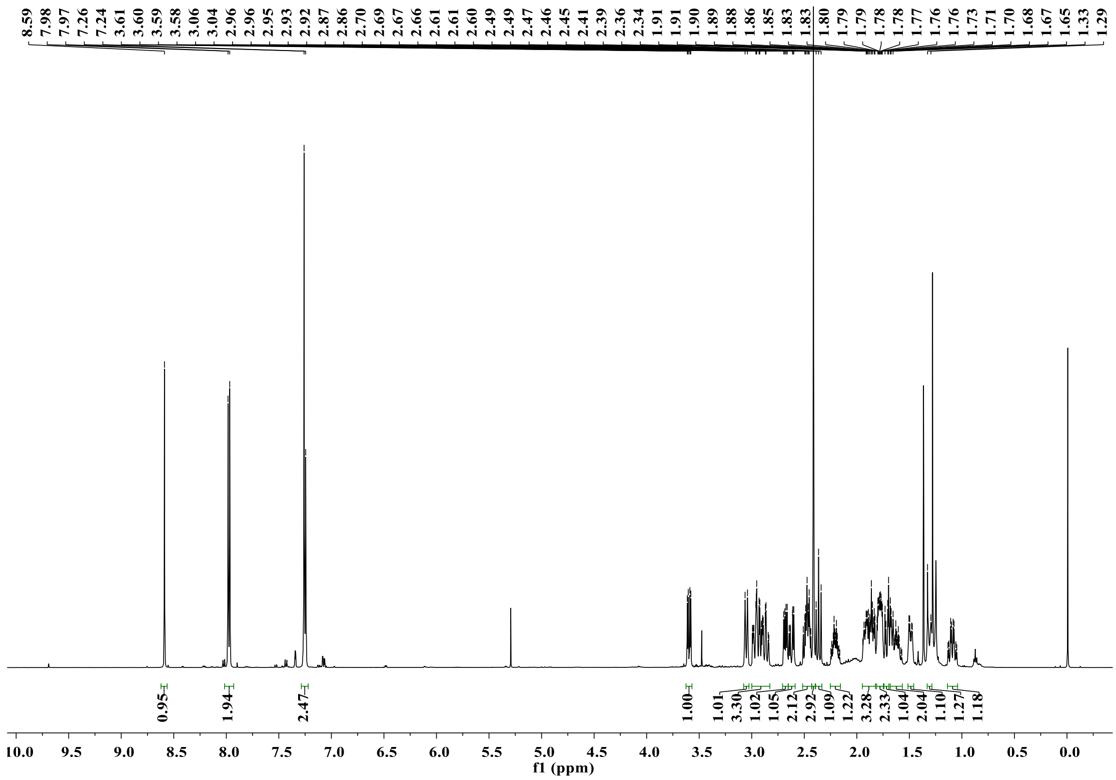

Figure S16. ^{1}H NMR spectrum of **SOP-2d**

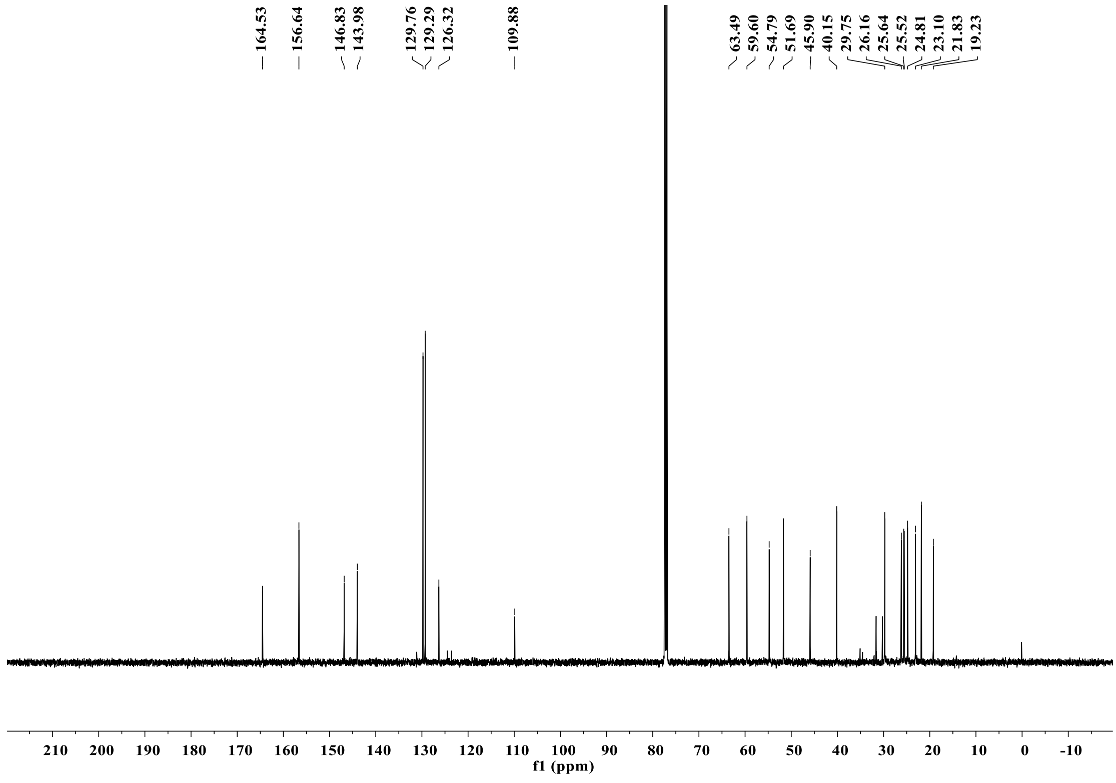

Figure S17. ^{13}C NMR spectrum of **SOP-2d**

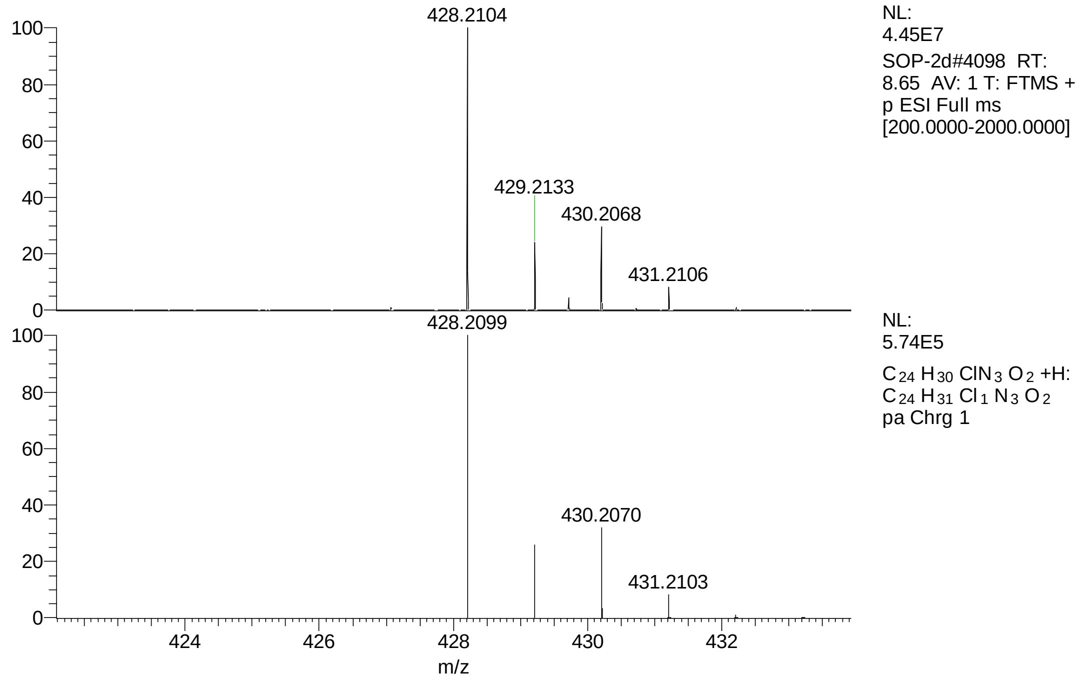

Figure S18. HR-ESI-MS spectrum of **SOP-2d**

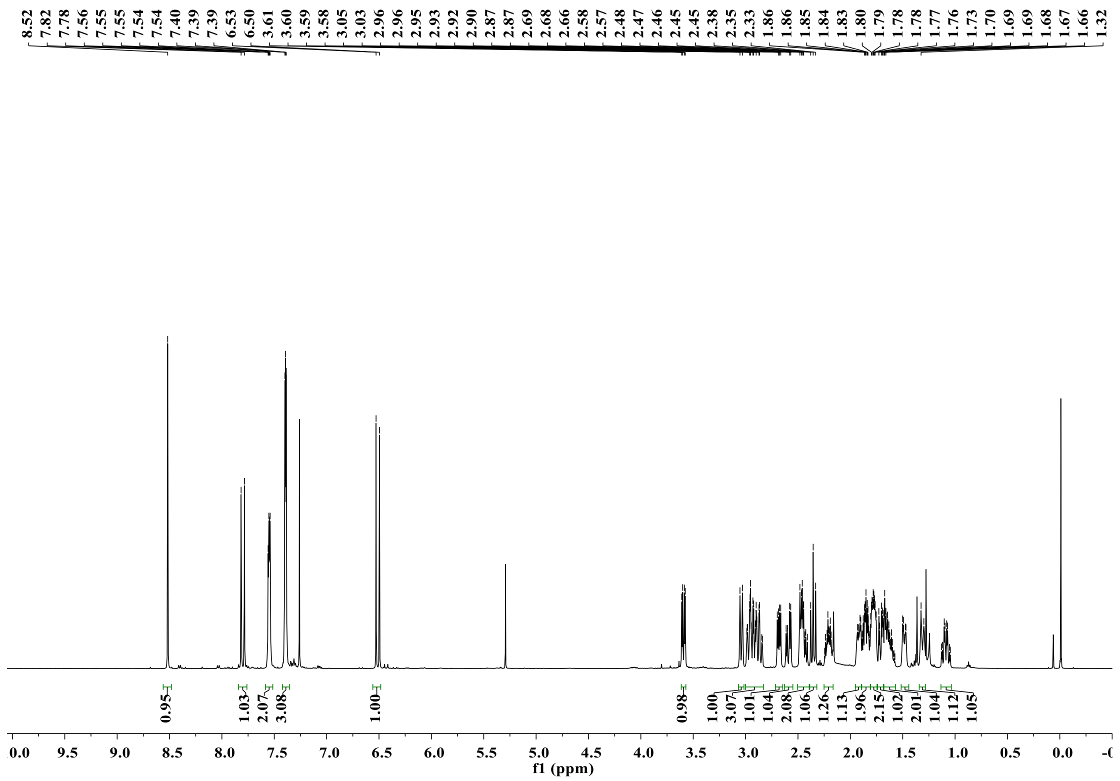

Figure S19. ^{1}H NMR spectrum of **SOP-2e**

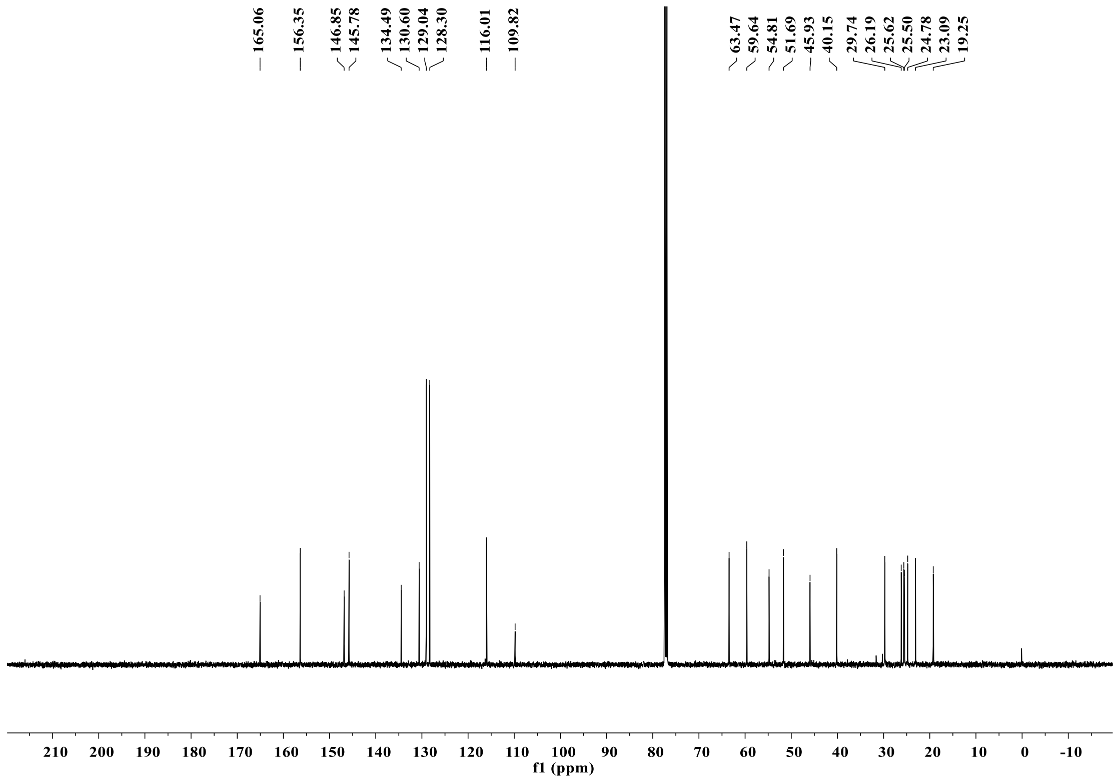

Figure S20. ^{13}C NMR spectrum of **SOP-2e**

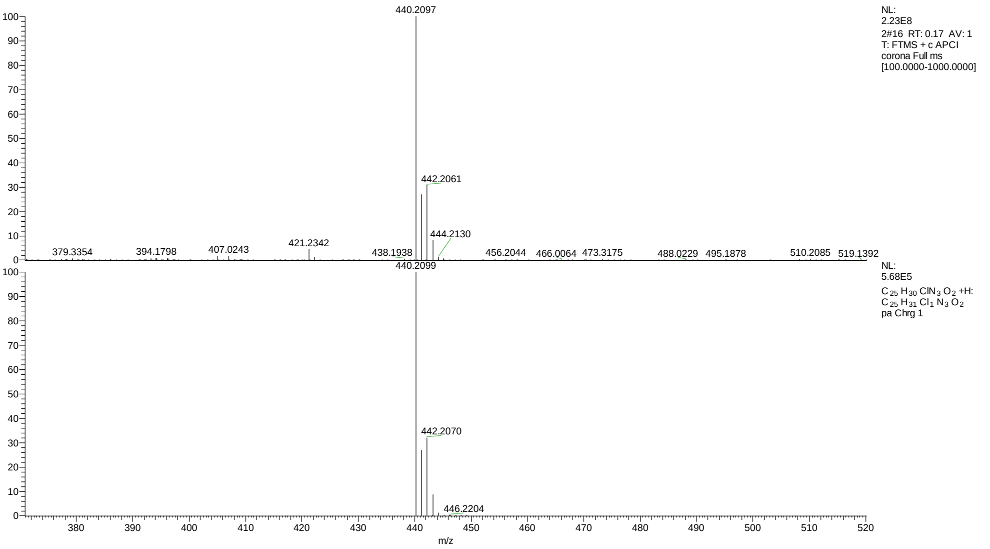

Figure S21. HR-ESI-MS spectrum of **SOP-2e**

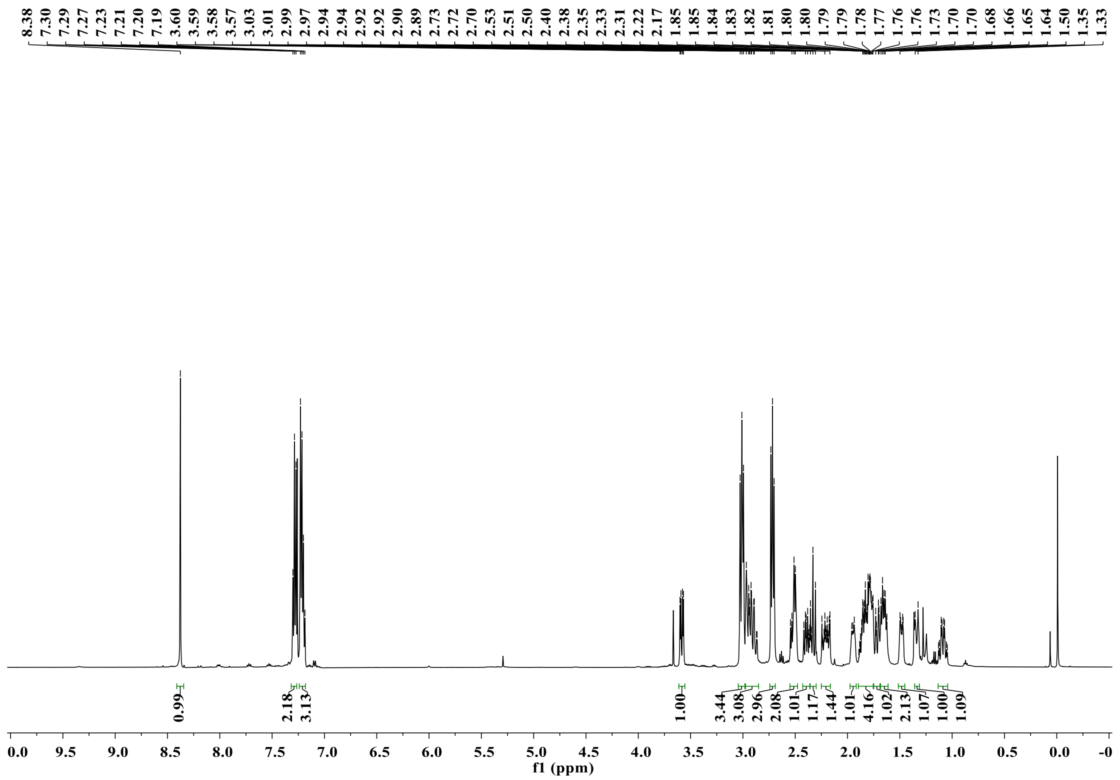

Figure S22. ^{1}H NMR spectrum of **SOP-2f**

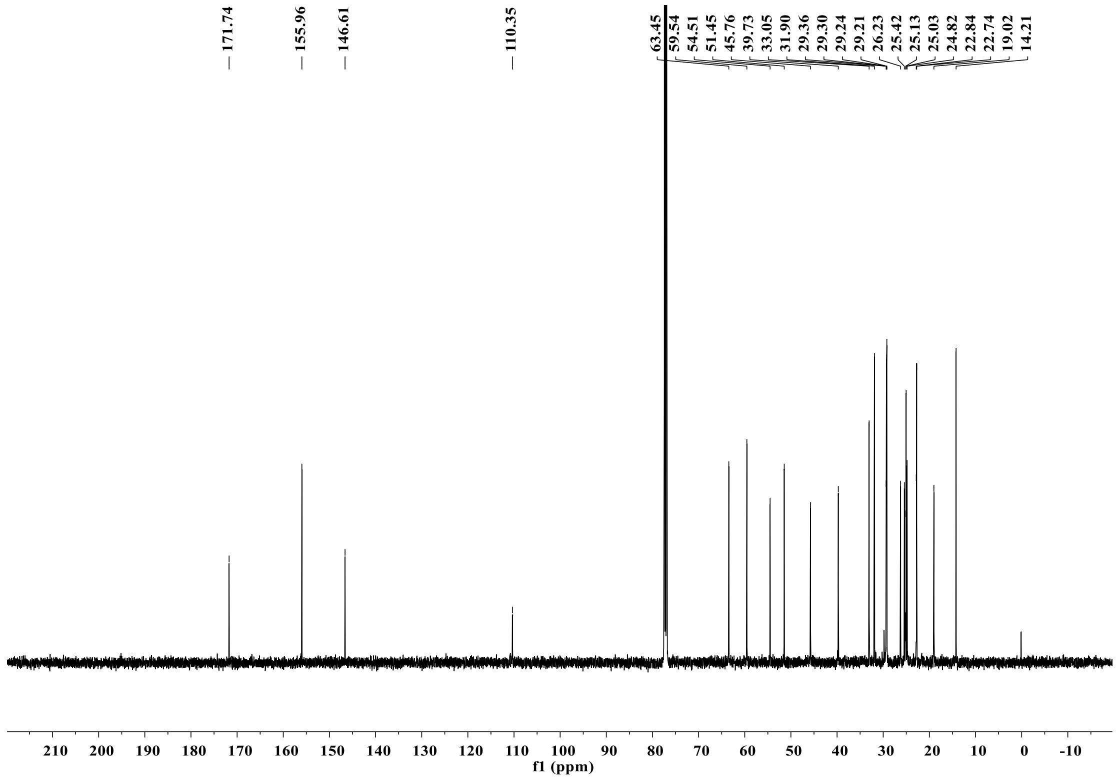

Figure S23. ^{13}C NMR spectrum of **SOP-2f**

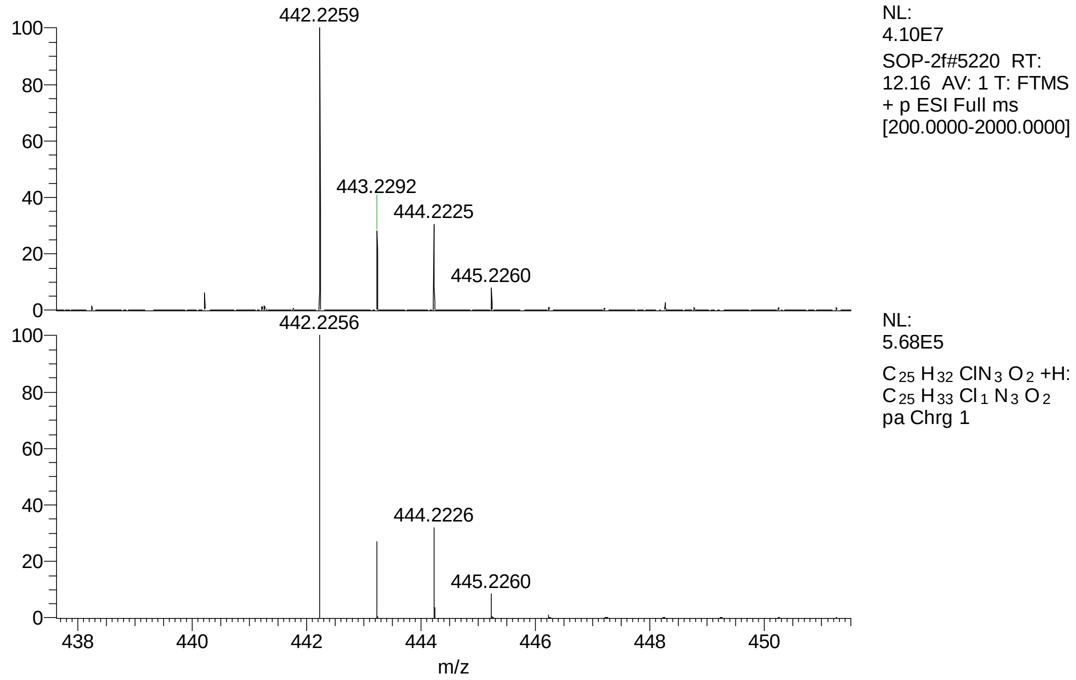

Figure S24. HR-ESI-MS spectrum of **SOP-2f**

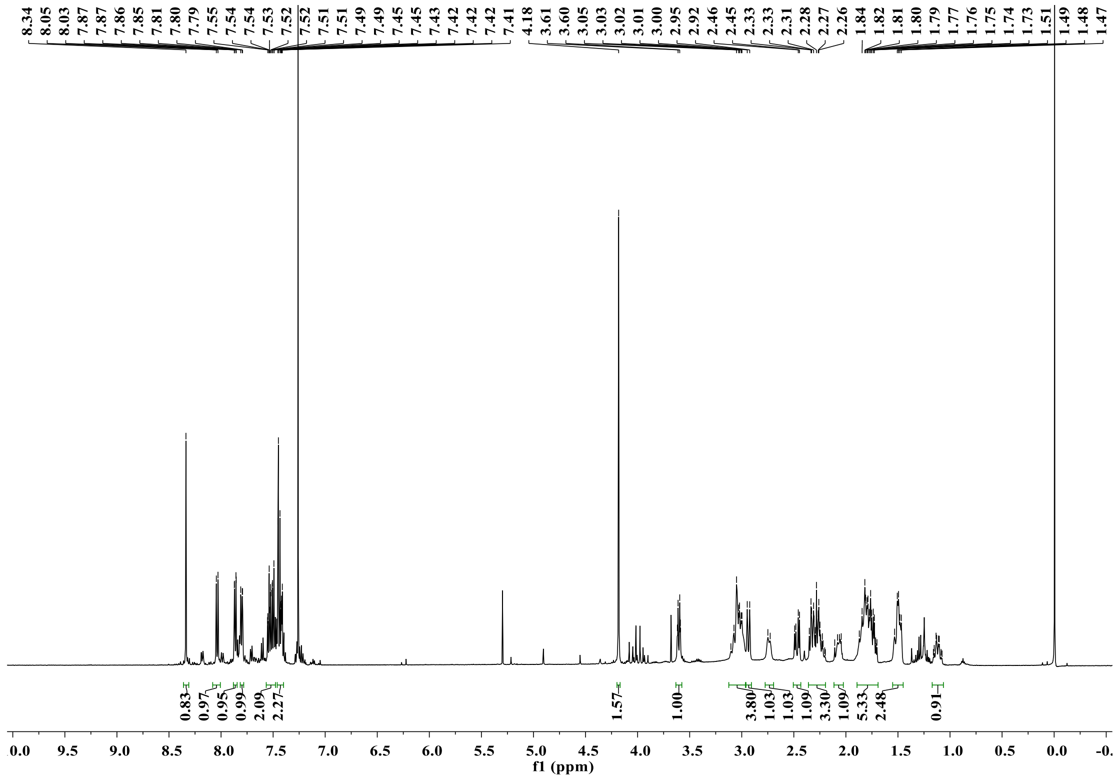

Figure S25. ^{1}H NMR spectrum of **SOP-2g**

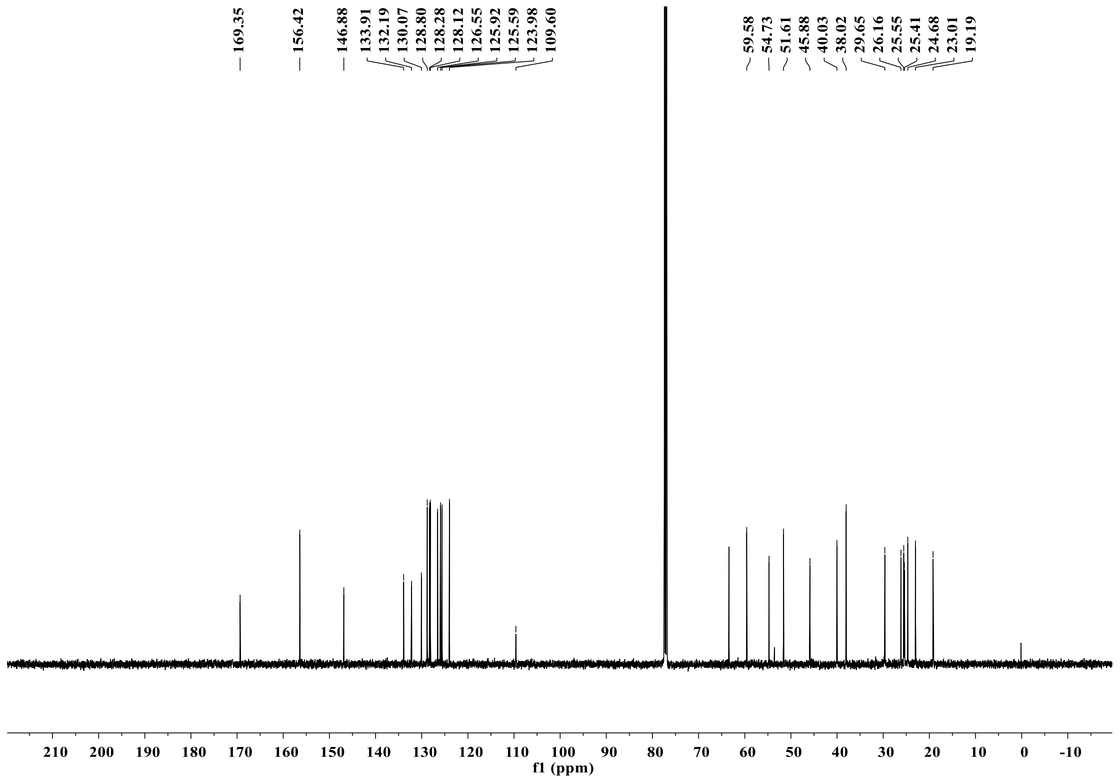

Figure S26. ^{13}C NMR spectrum of **SOP-2g**

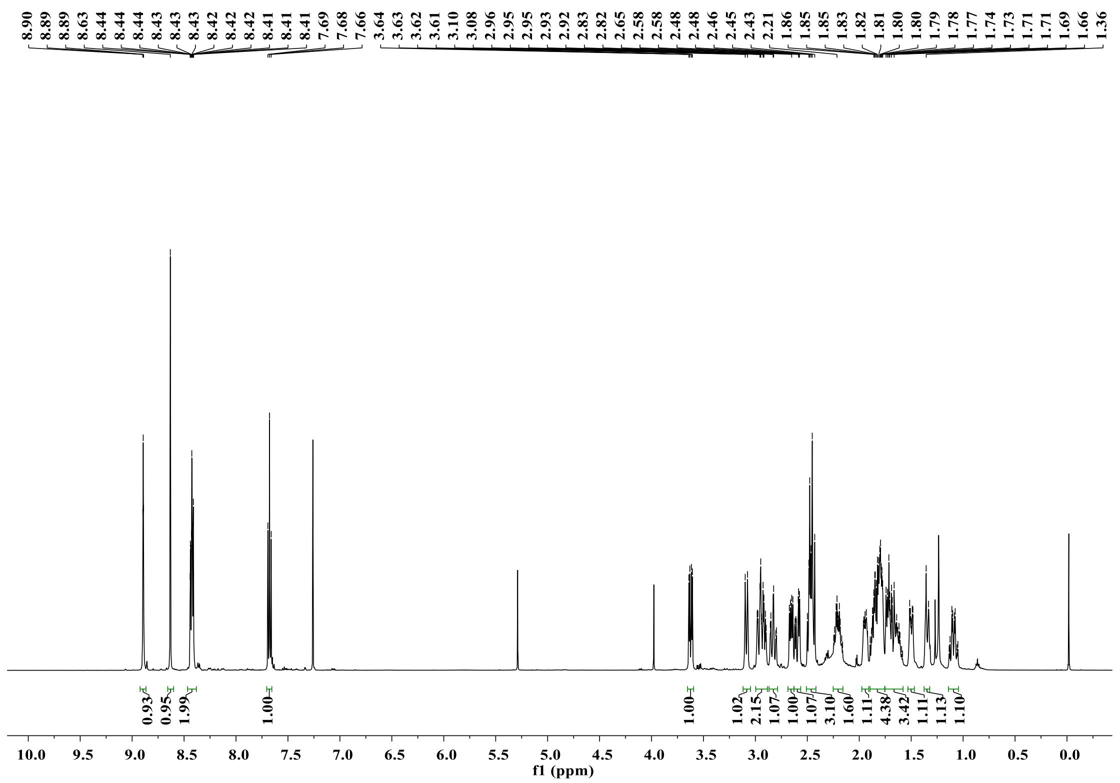

Figure S27. ^{1}H NMR spectrum of **SOP-2h**

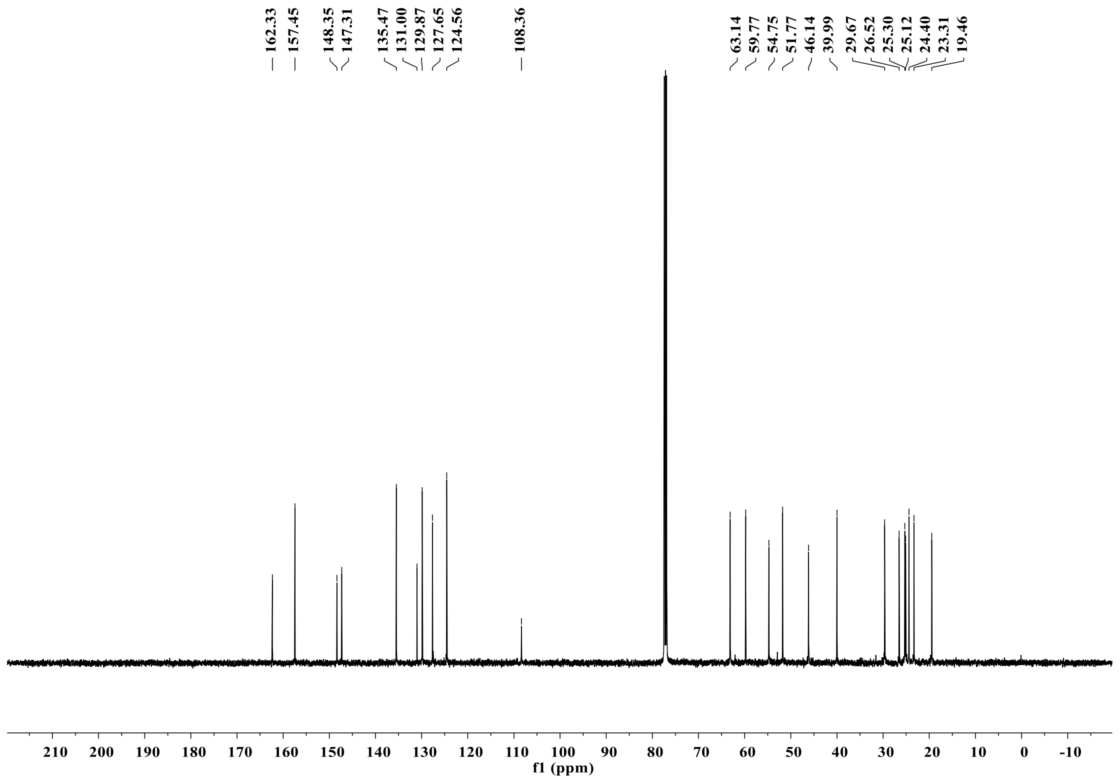

Figure S28. ^{13}C NMR spectrum of **SOP-2h**

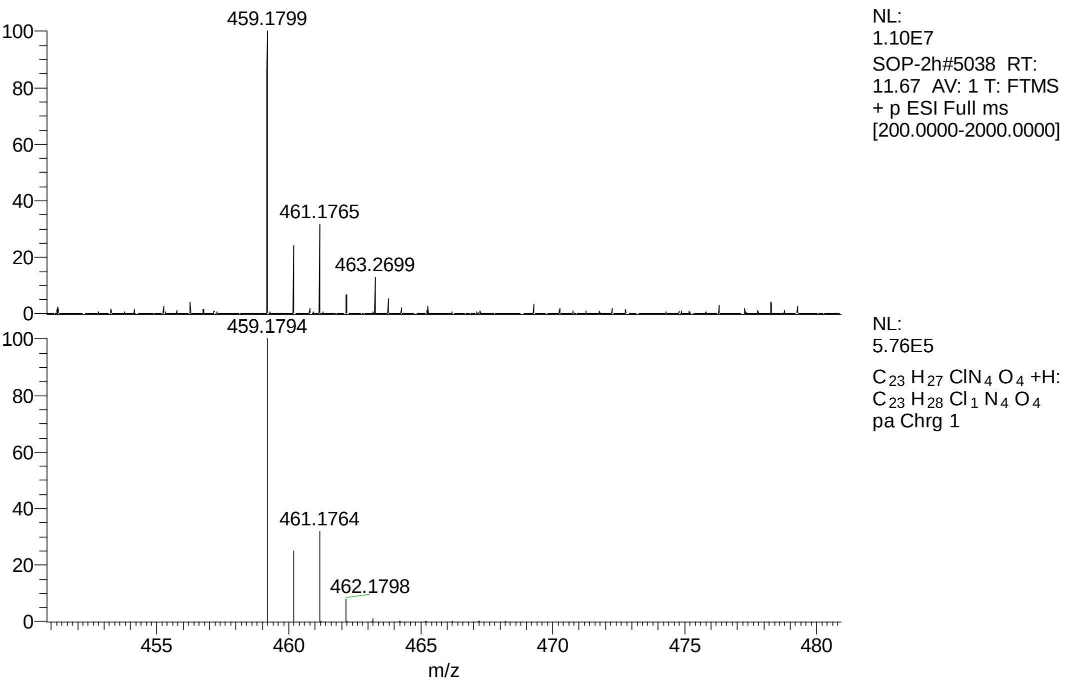

Figure S29. HR-ESI-MS spectrum of **SOP-2h**

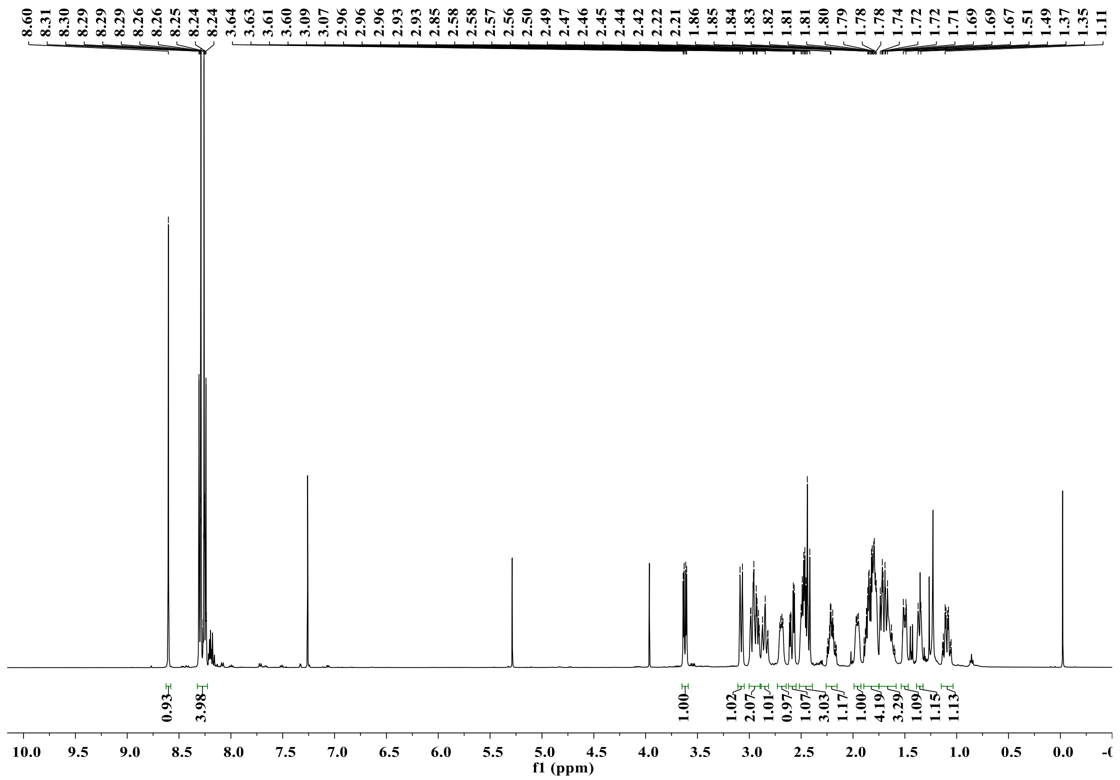

Figure S30. ^{1}H NMR spectrum of **SOP-2i**

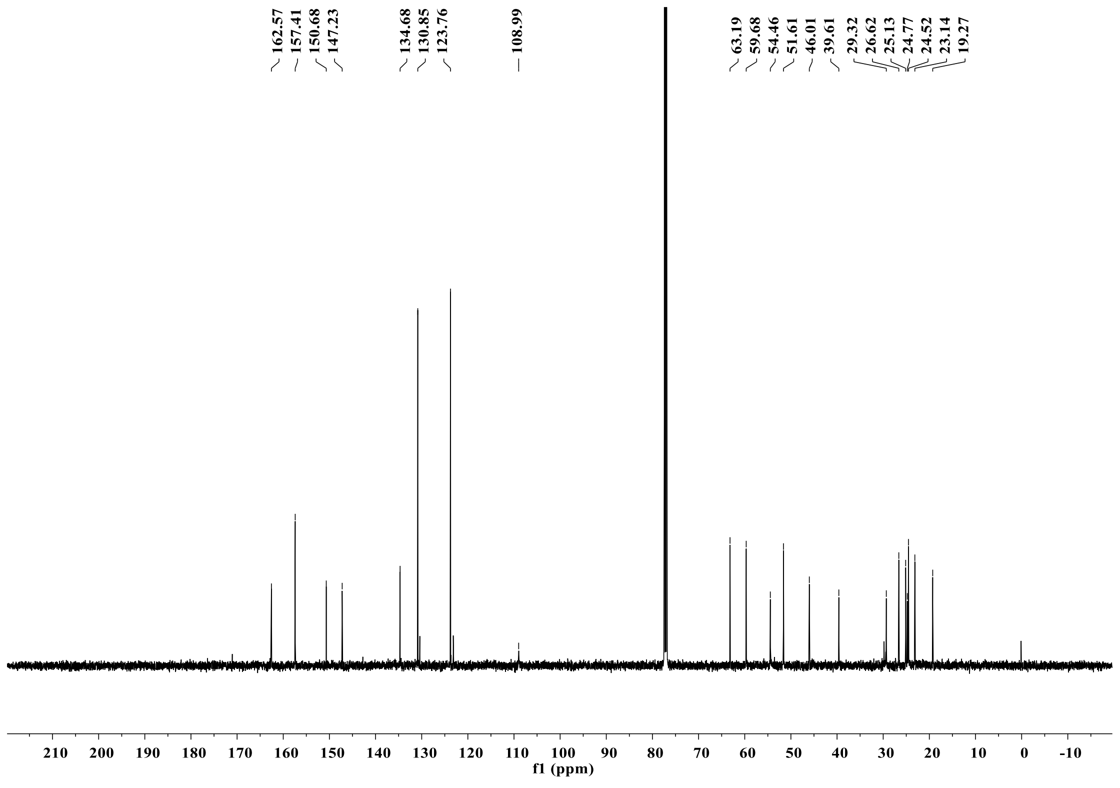

Figure S31. ^{13}C NMR spectrum of **SOP-2i**

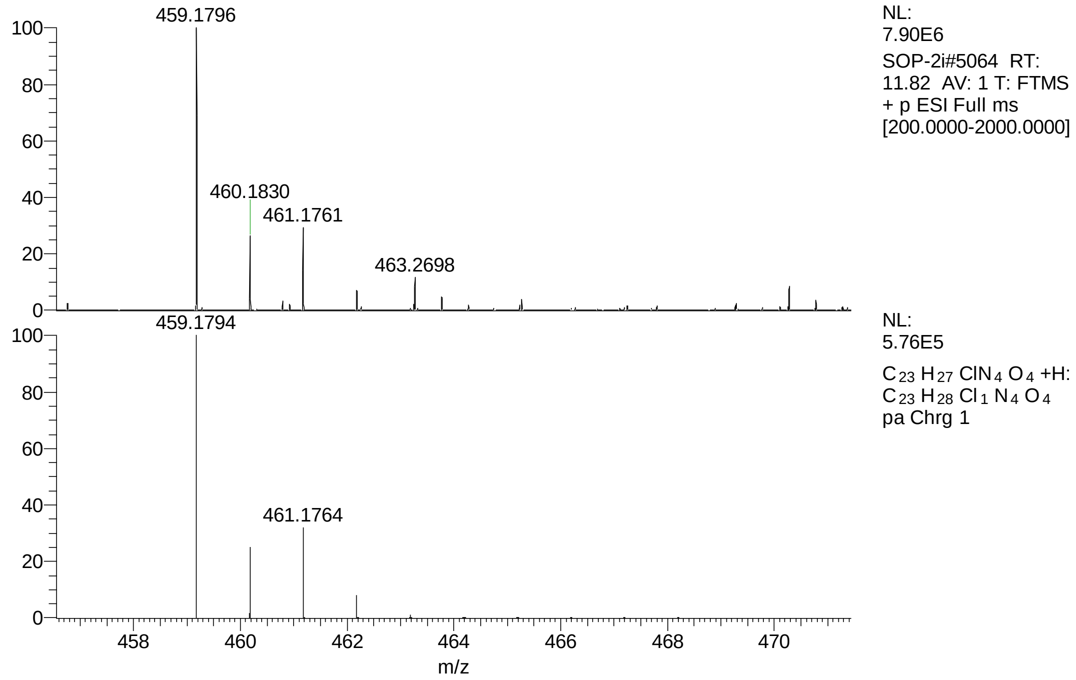

Figure S32. HR-ESI-MS spectrum of **SOP-2i**

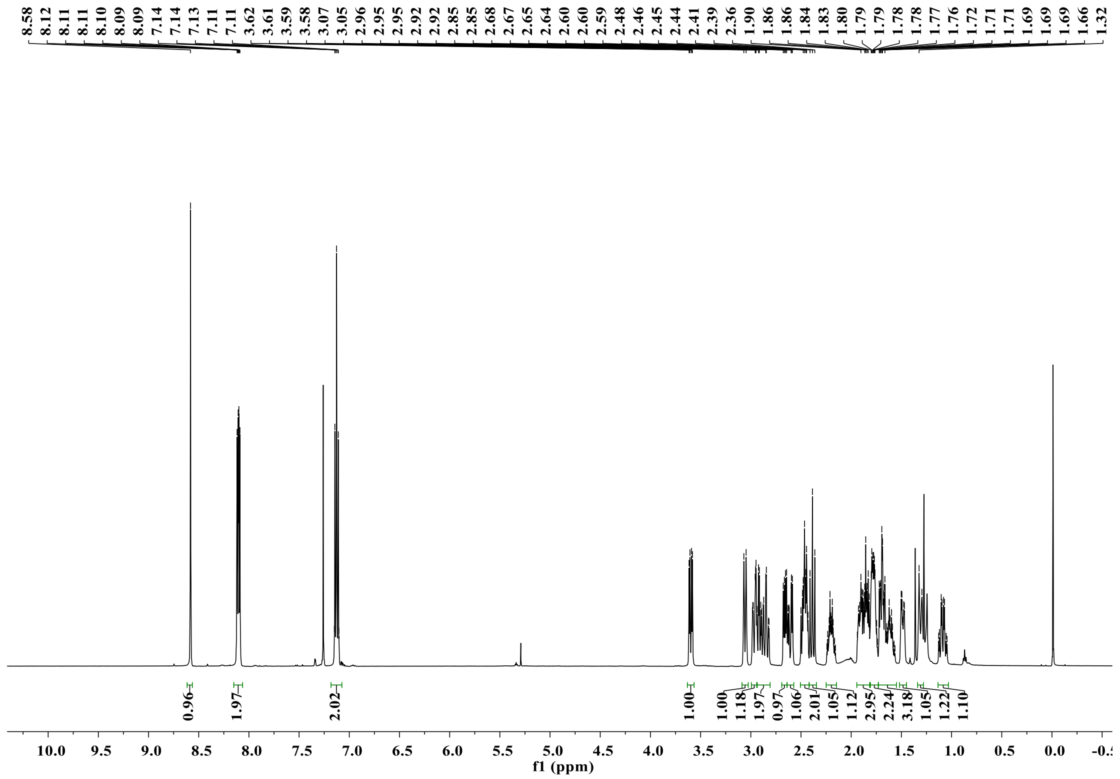

Figure S33. ^{1}H NMR spectrum of **SOP-2j**

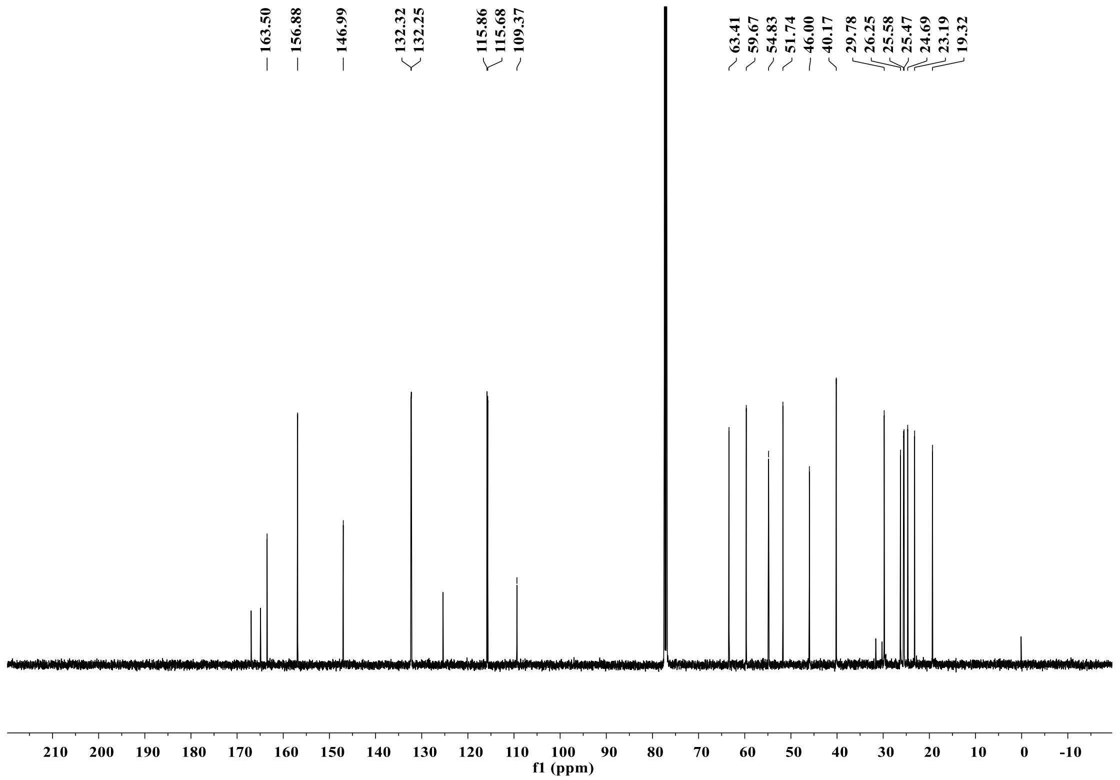

Figure S34. ^{13}C NMR spectrum of **SOP-2j**

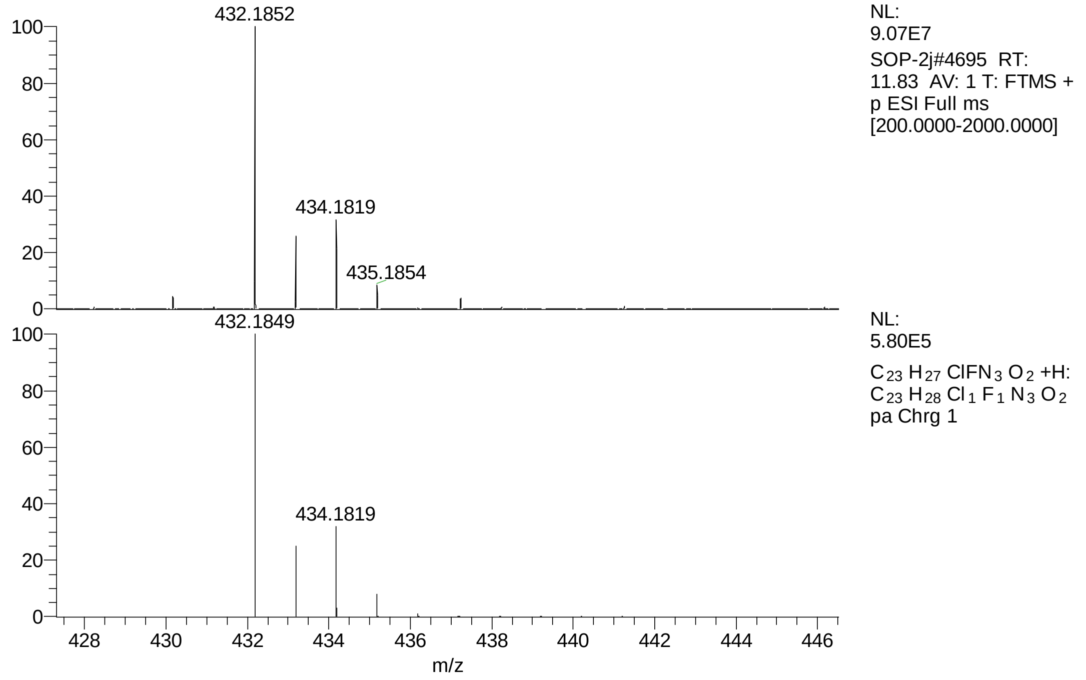

Figure S35. HR-ESI-MS spectrum of **SOP-2j**

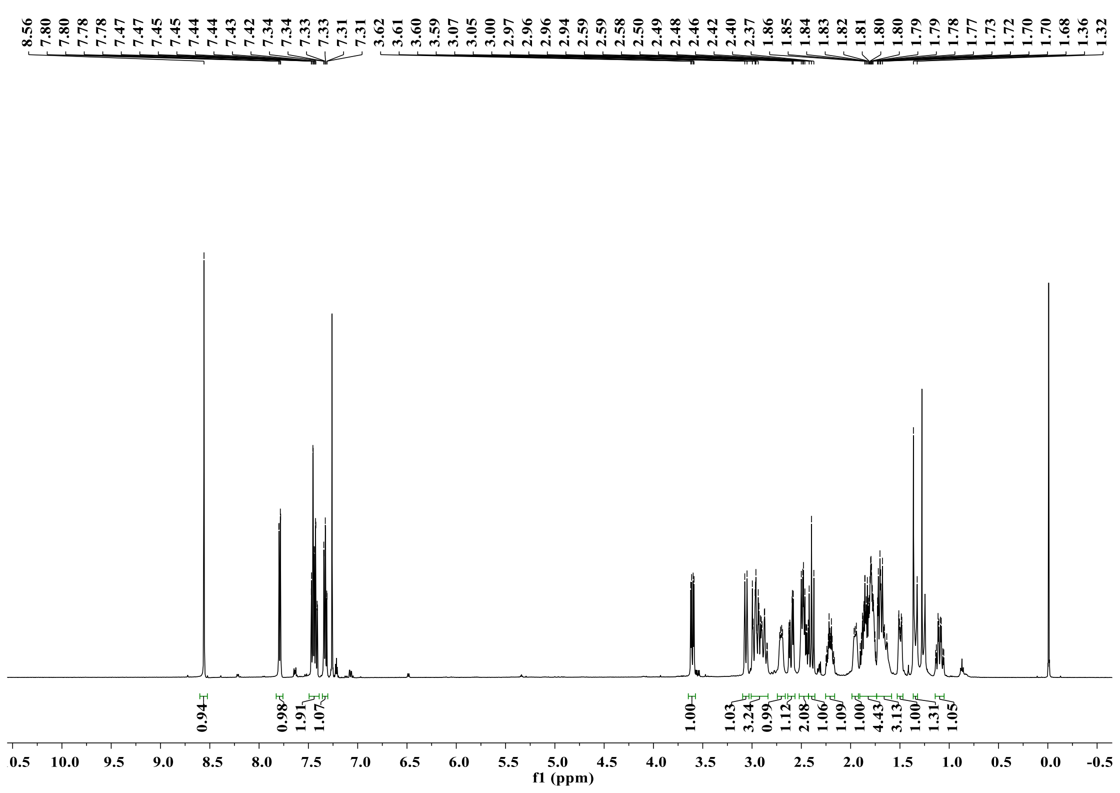

Figure S36. ^{1}H NMR spectrum of **SOP-2k**

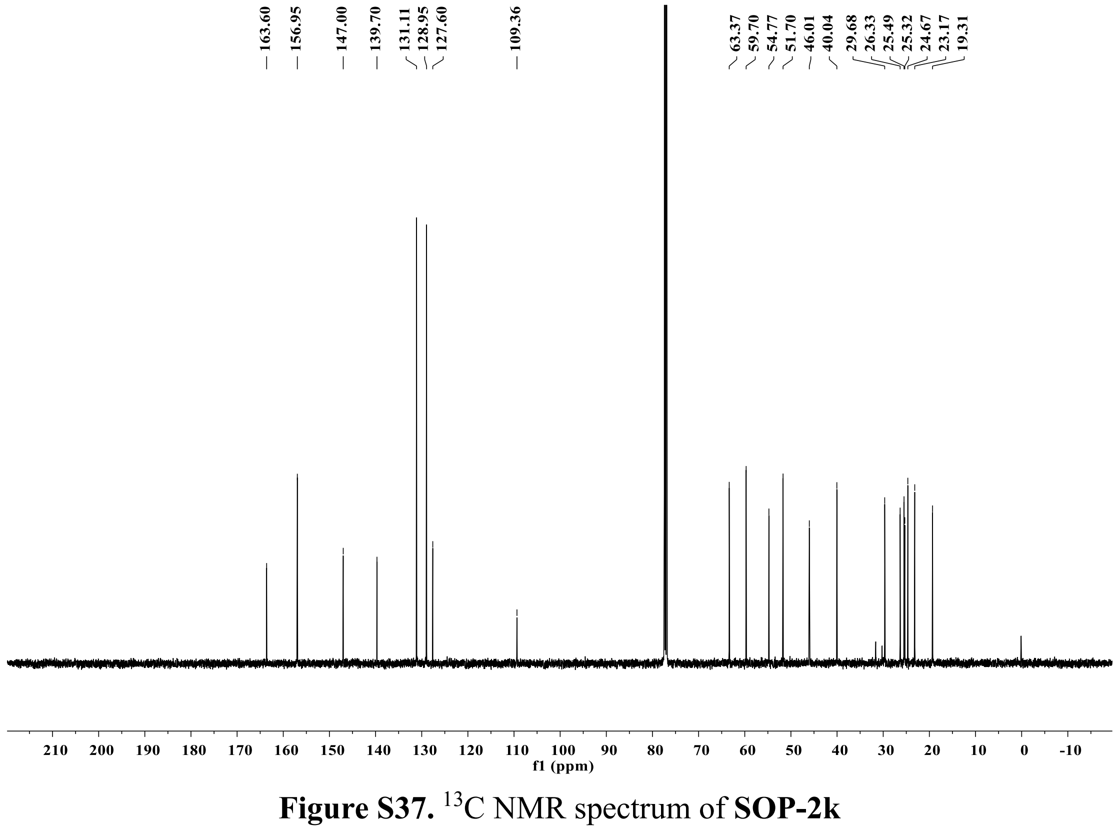

Figure S37. ^{13}C NMR spectrum of **SOP-2k**

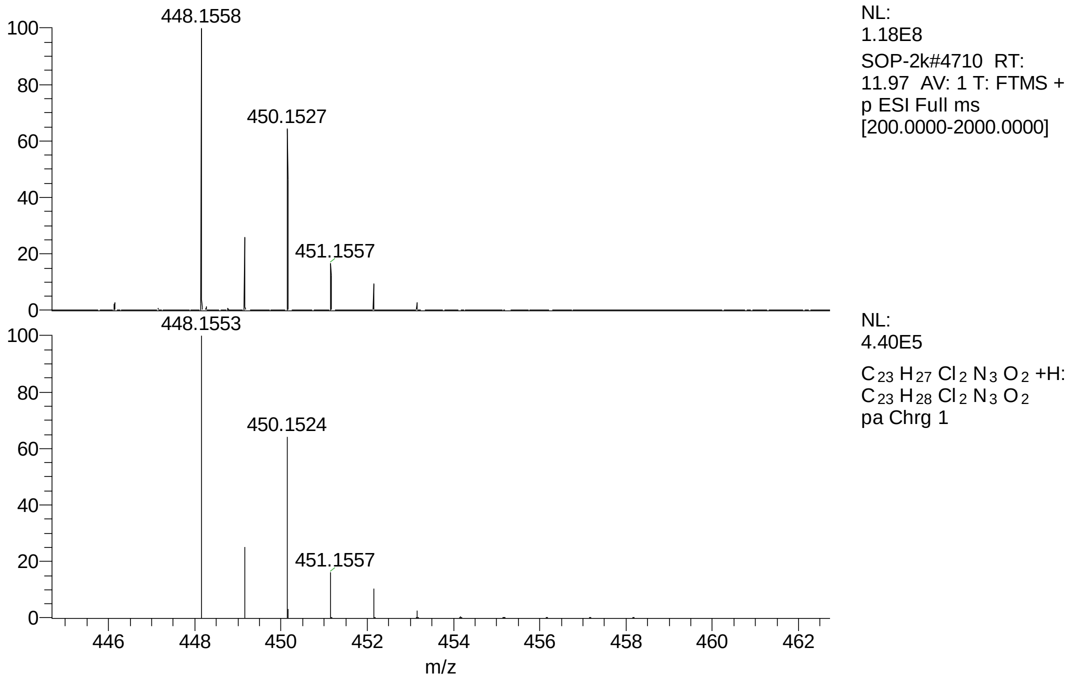

Figure S38. HR-ESI-MS spectrum of **SOP-2k**

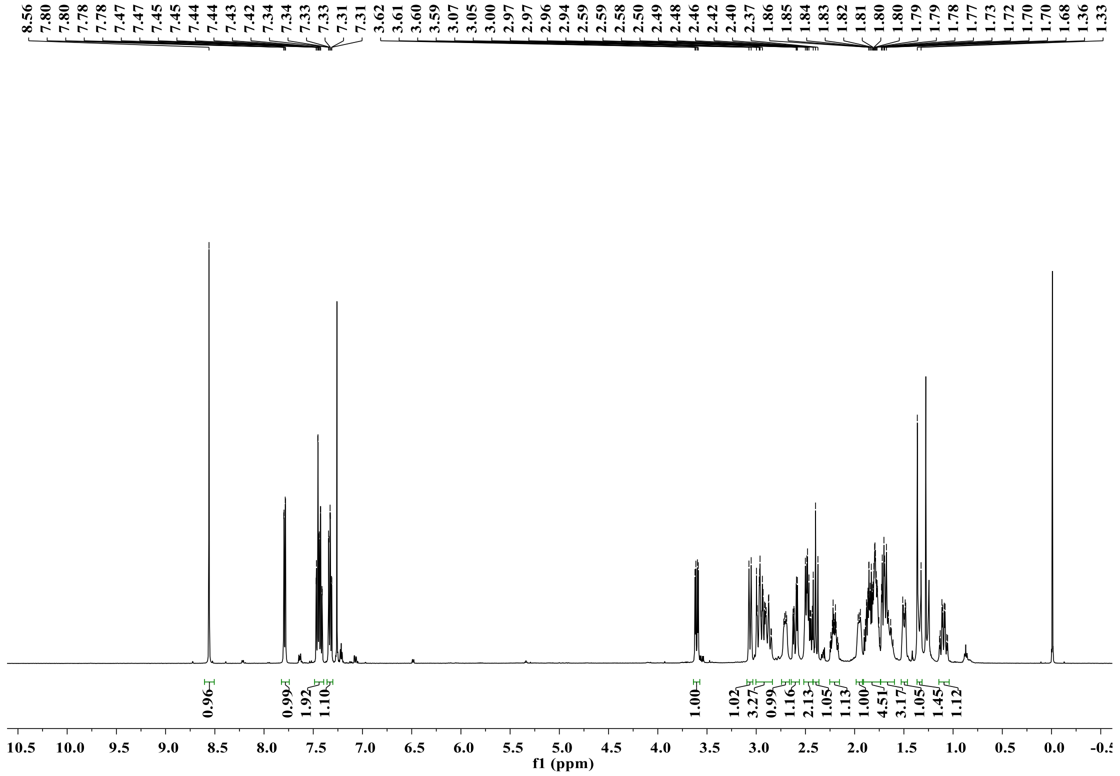

Figure S39. ^{1}H NMR spectrum of **SOP-2l**

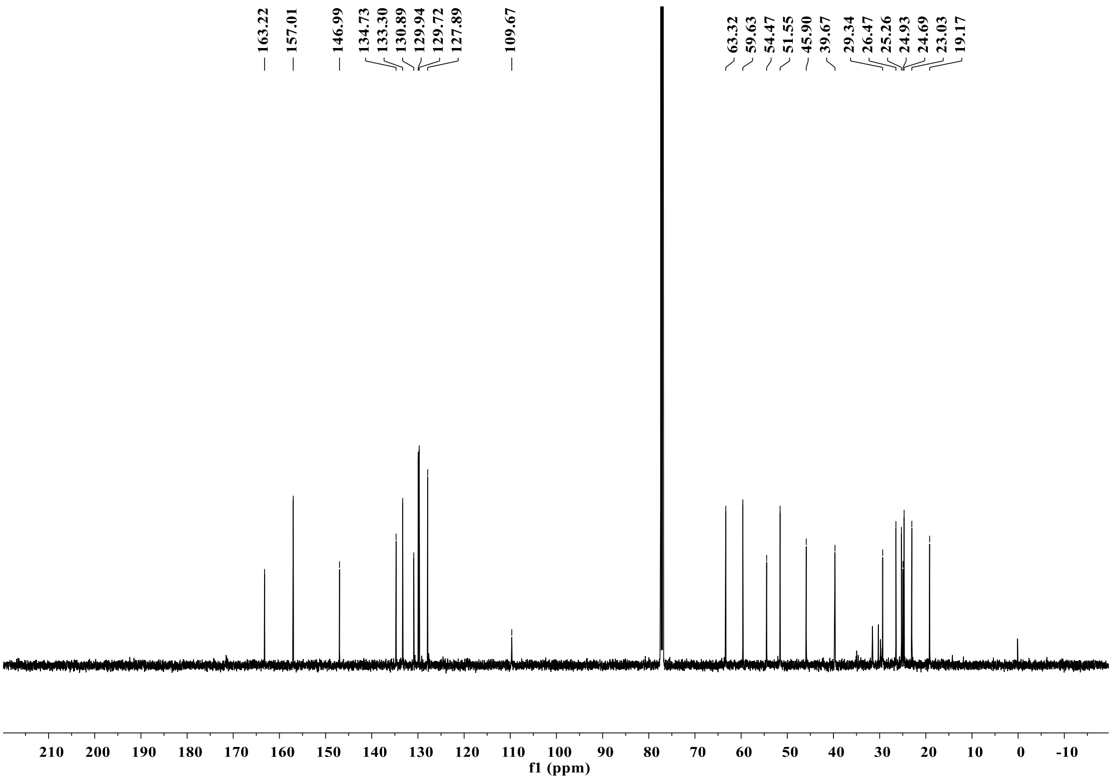

Figure S40. ^{13}C NMR spectrum of **SOP-2l**

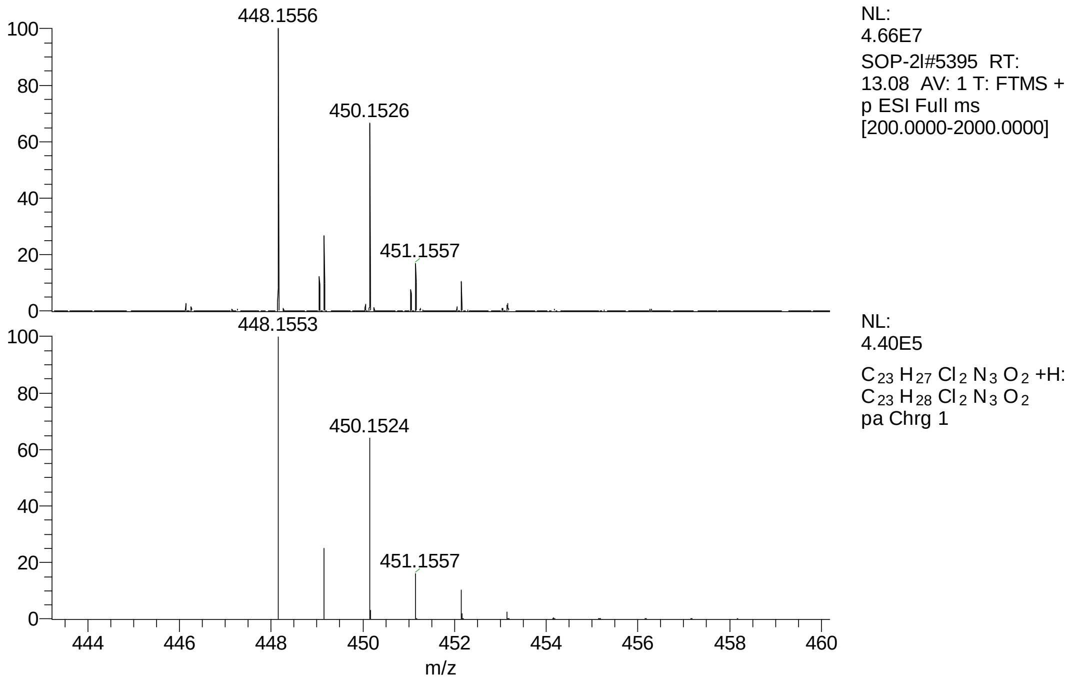

Figure S41. HR-ESI-MS spectrum of **SOP-2l**

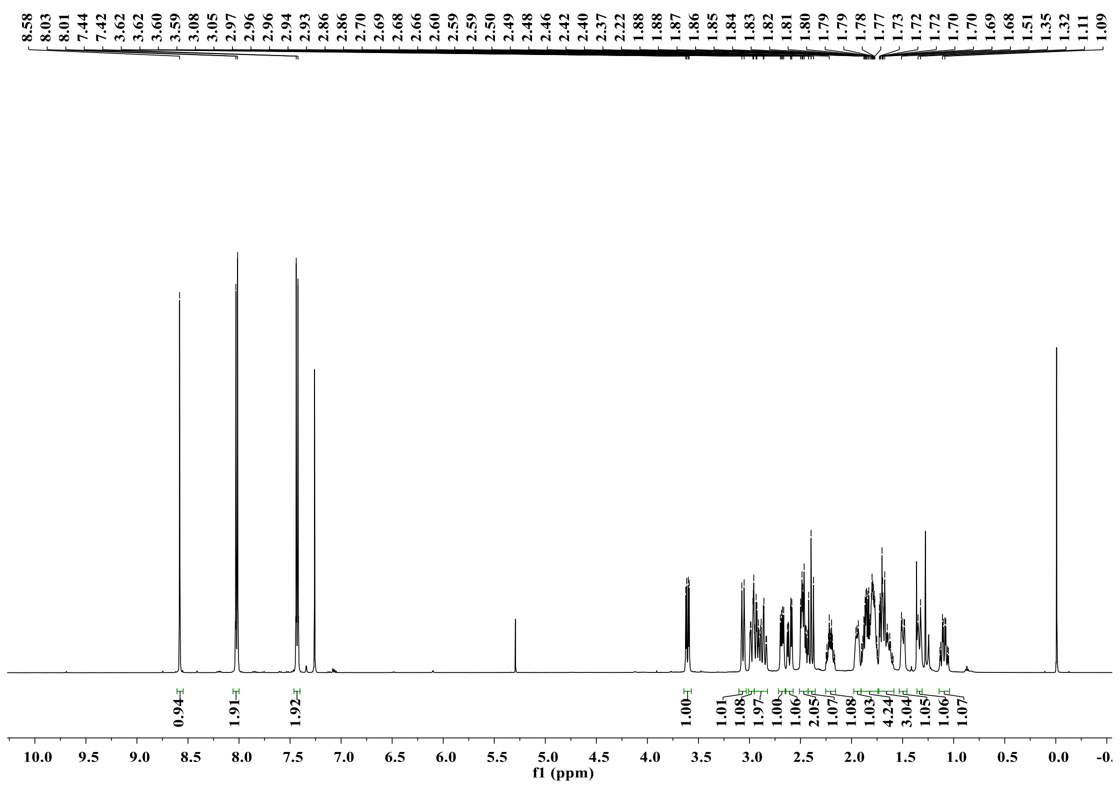

Figure S42. ^{1}H NMR spectrum of **SOP-2m**

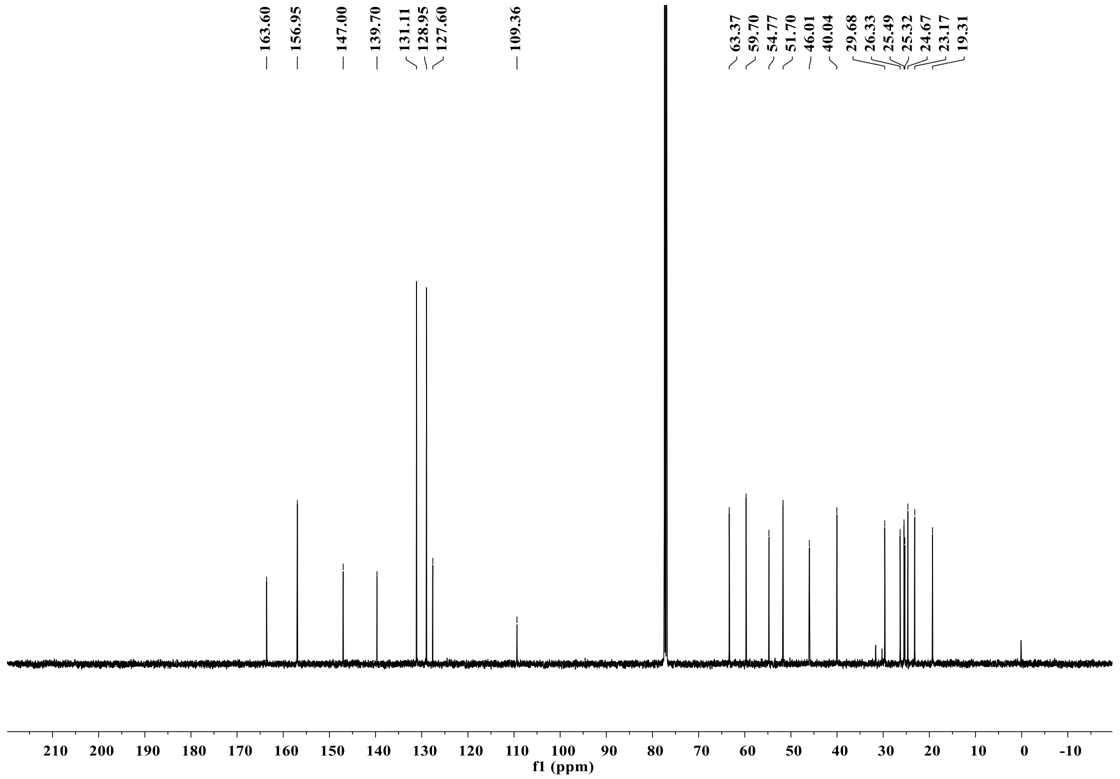

Figure S43. ^{13}C NMR spectrum of **SOP-2m**

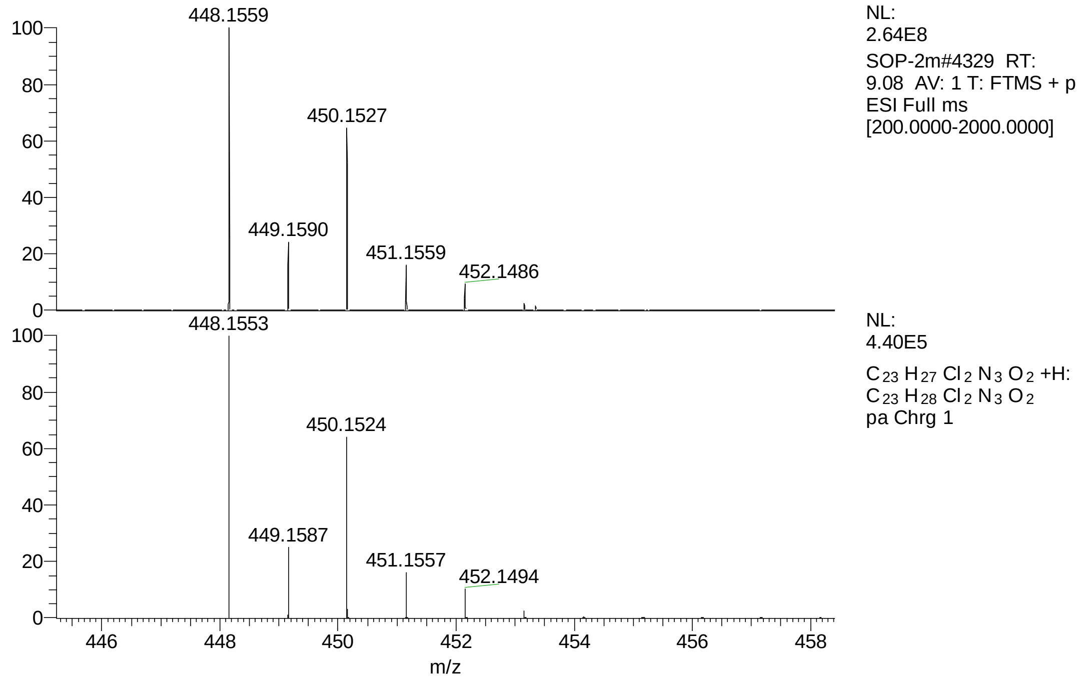

Figure S44. HR-ESI-MS spectrum of **SOP-2m**

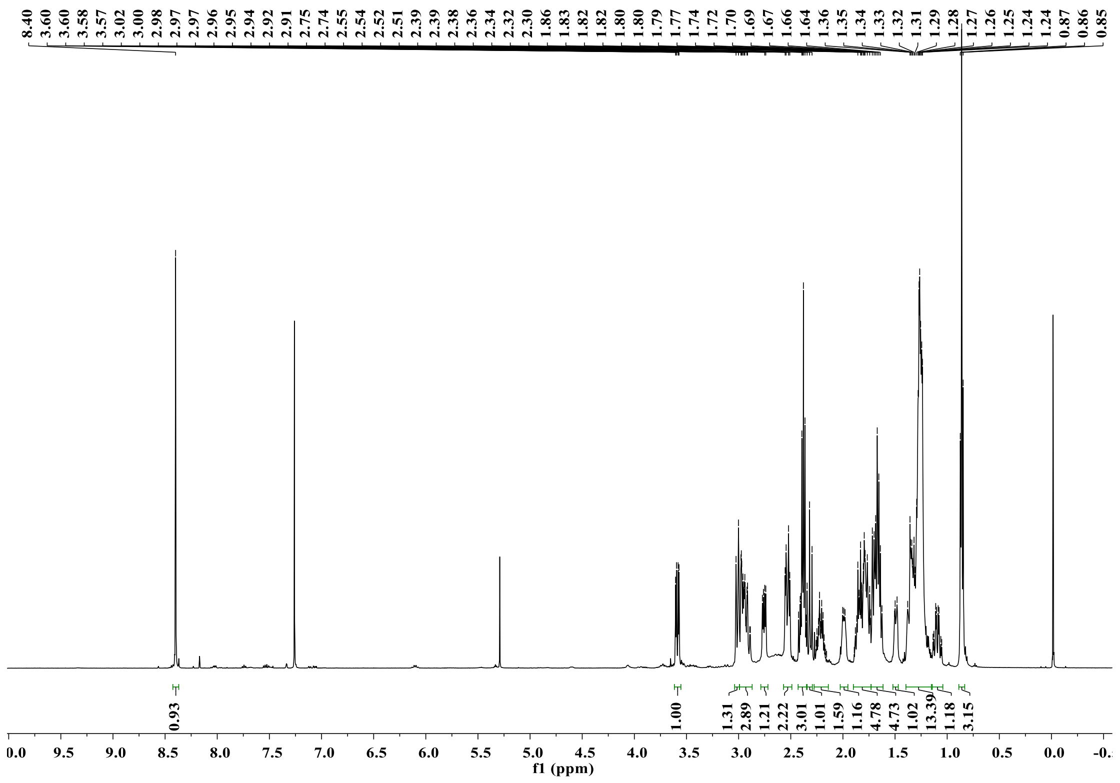

Figure S45. ^{1}H NMR spectrum of **SOP-2n**

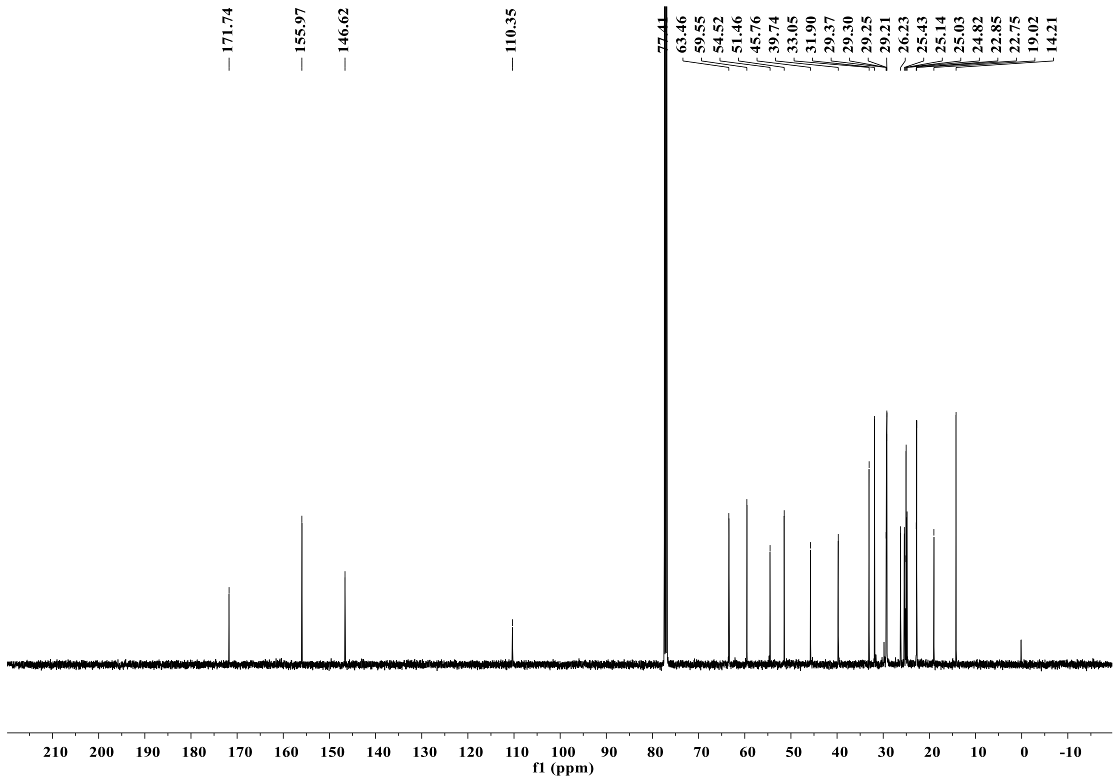

Figure S46. ^{13}C NMR spectrum of **SOP-2n**

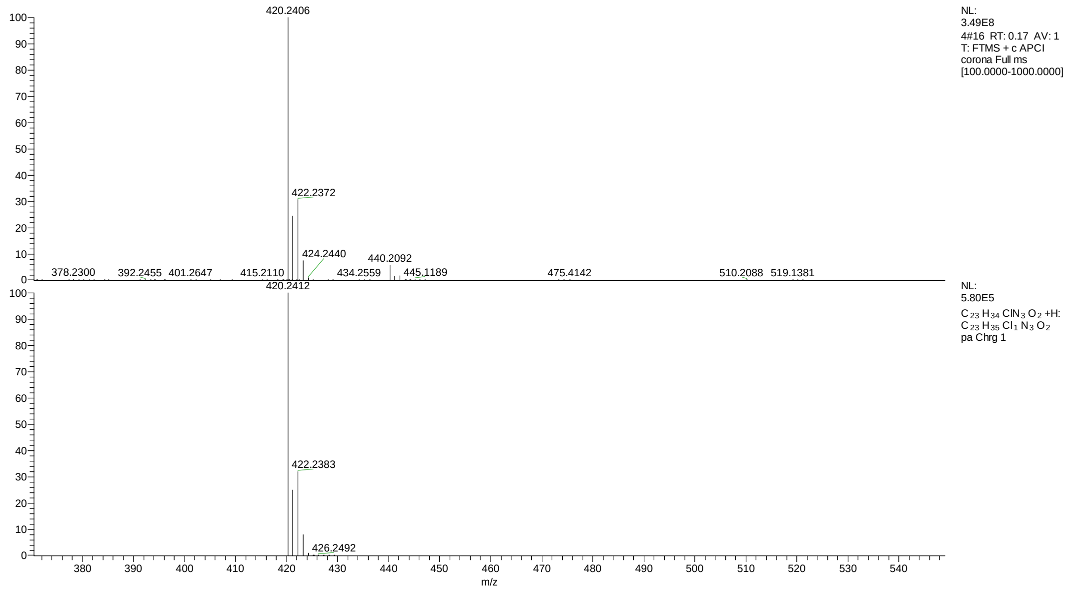

Figure S47. HR-ESI-MS spectrum of **SOP-2n**

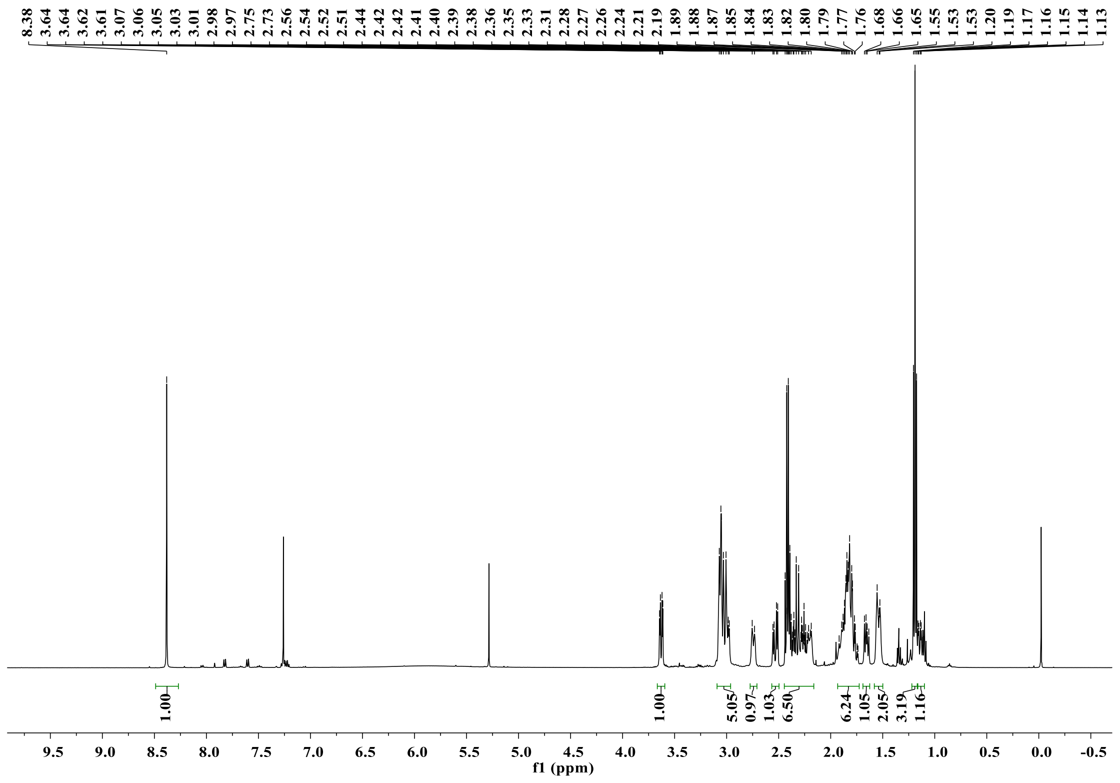

Figure S48. ^{1}H NMR spectrum of **SOP-2o**

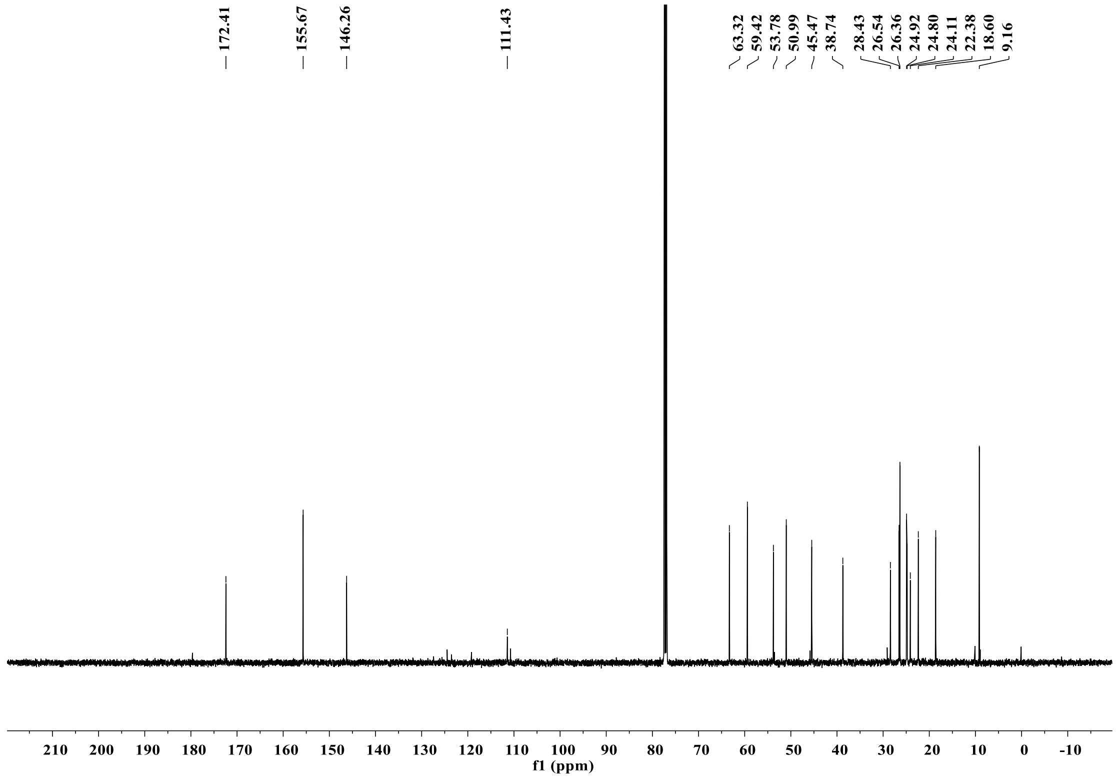

Figure S49. ^{13}C NMR spectrum of **SOP-2o**

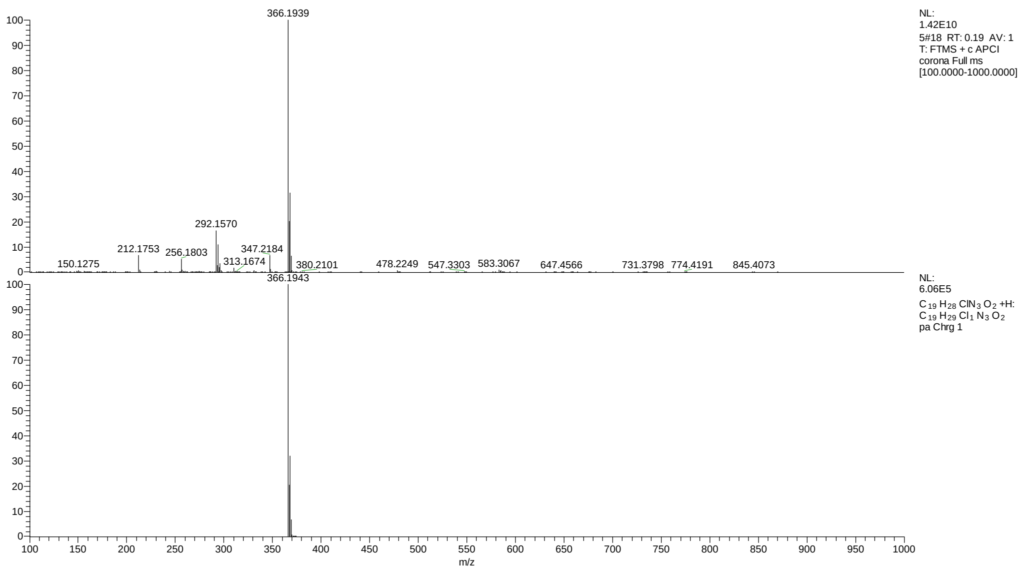

Figure S50. HR-ESI-MS spectrum of **SOP-2o**

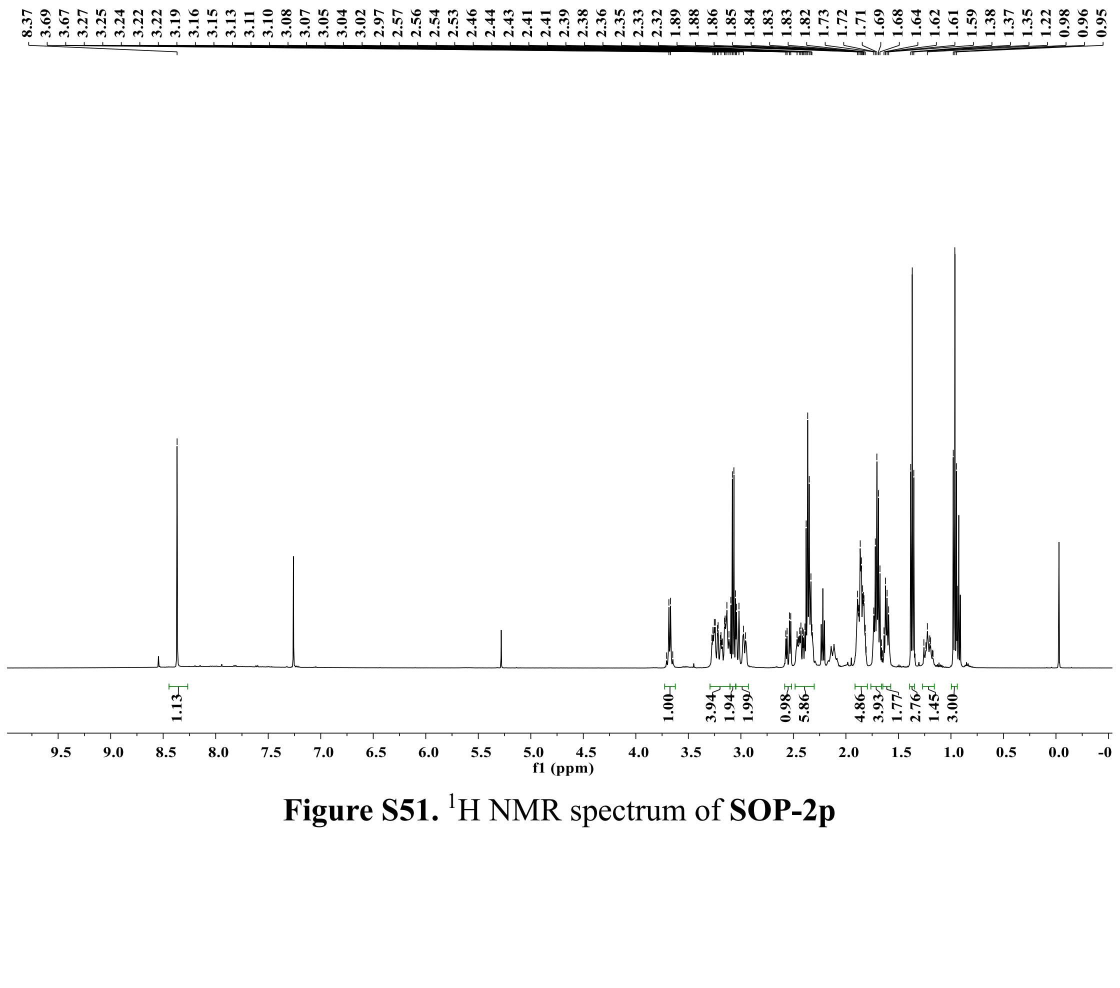

Figure S51. ^{1}H NMR spectrum of **SOP-2p**

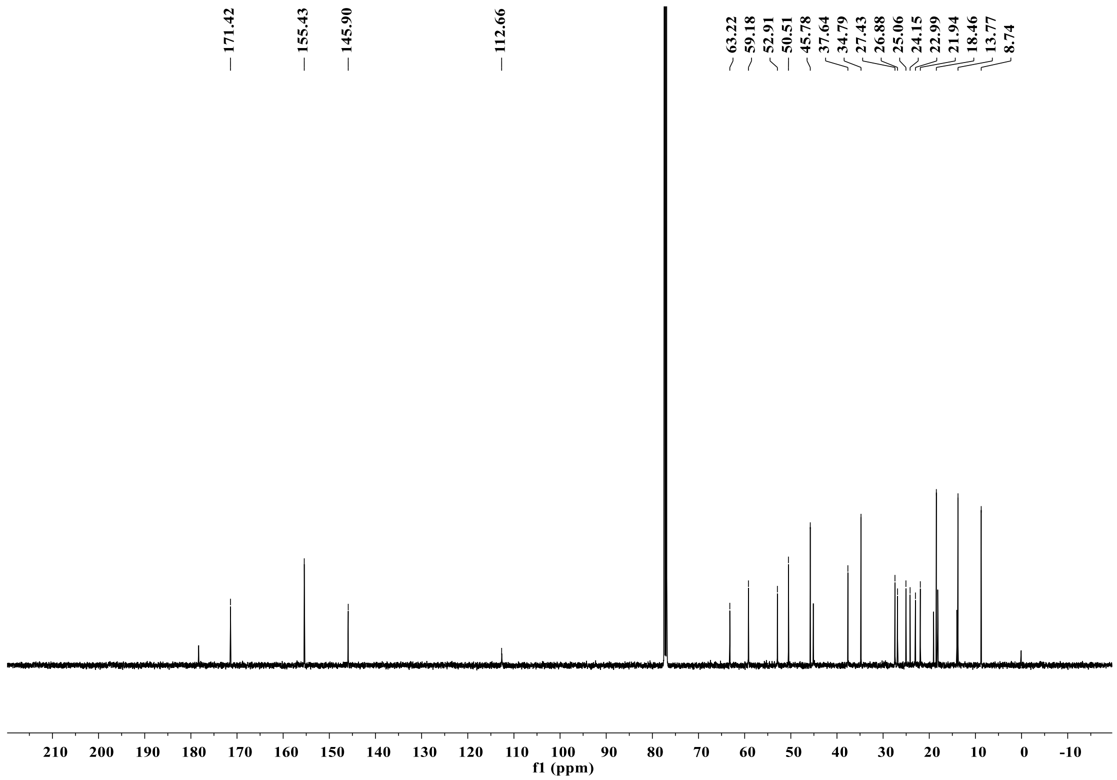

Figure S52. ^{13}C NMR spectrum of **SOP-2p**

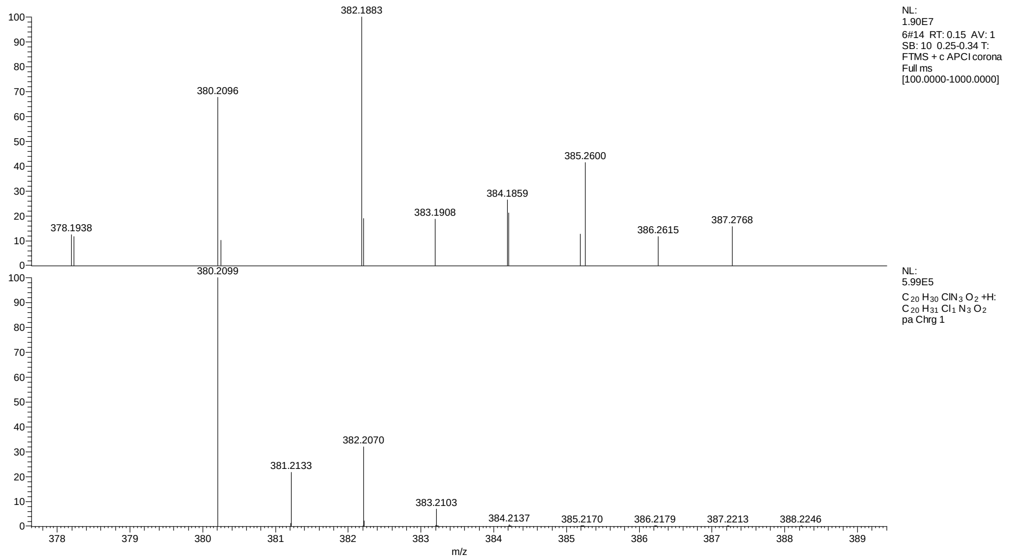

Figure S53. HR-ESI-MS spectrum of **SOP-2p**

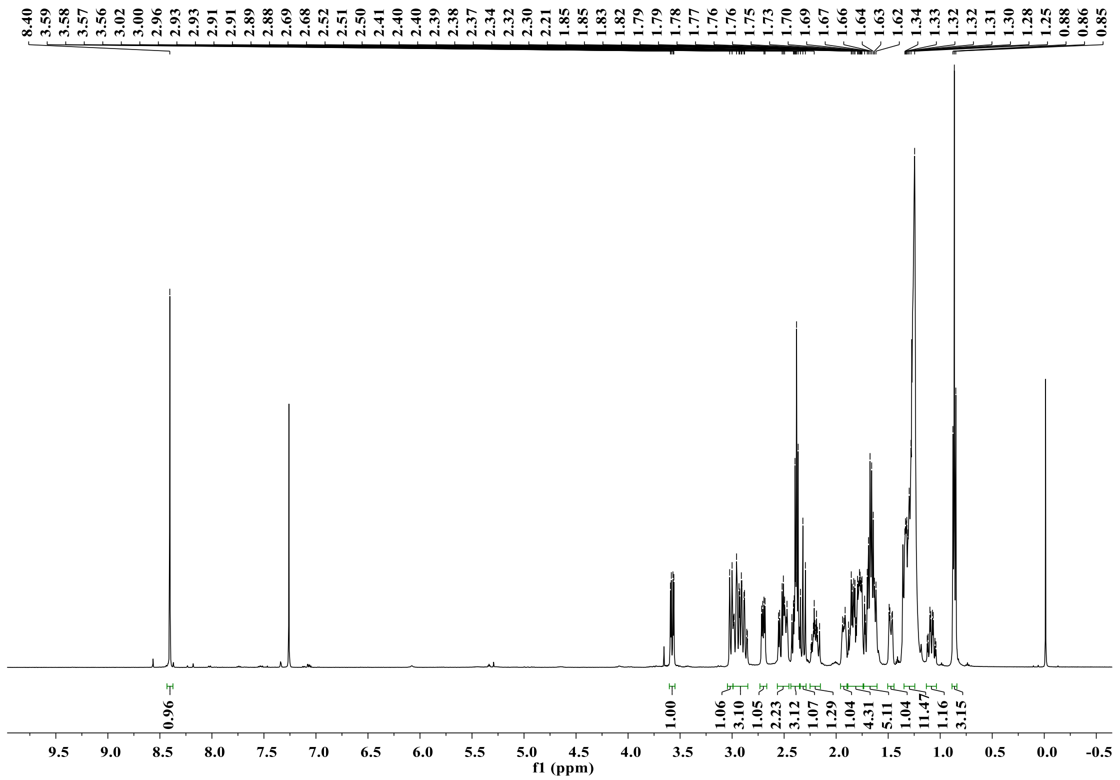

Figure S54. ^{1}H NMR spectrum of **SOP-2q**

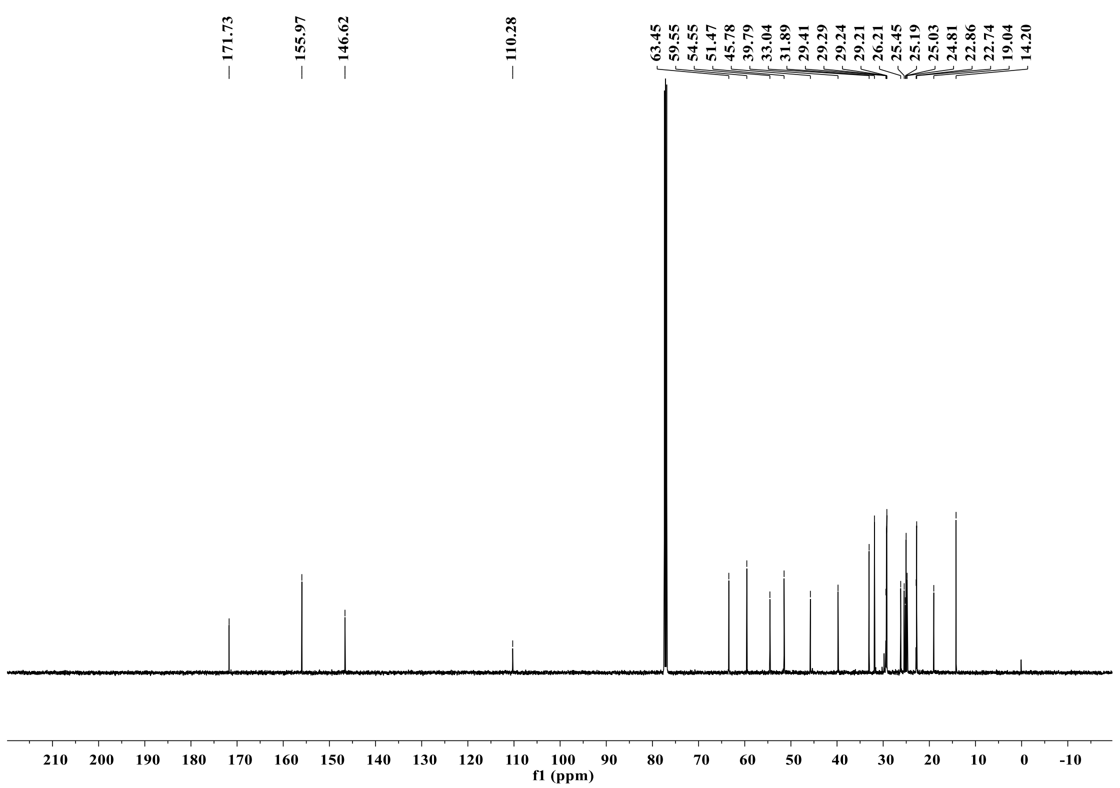

Figure S55. ^{13}C NMR spectrum of **SOP-2q**

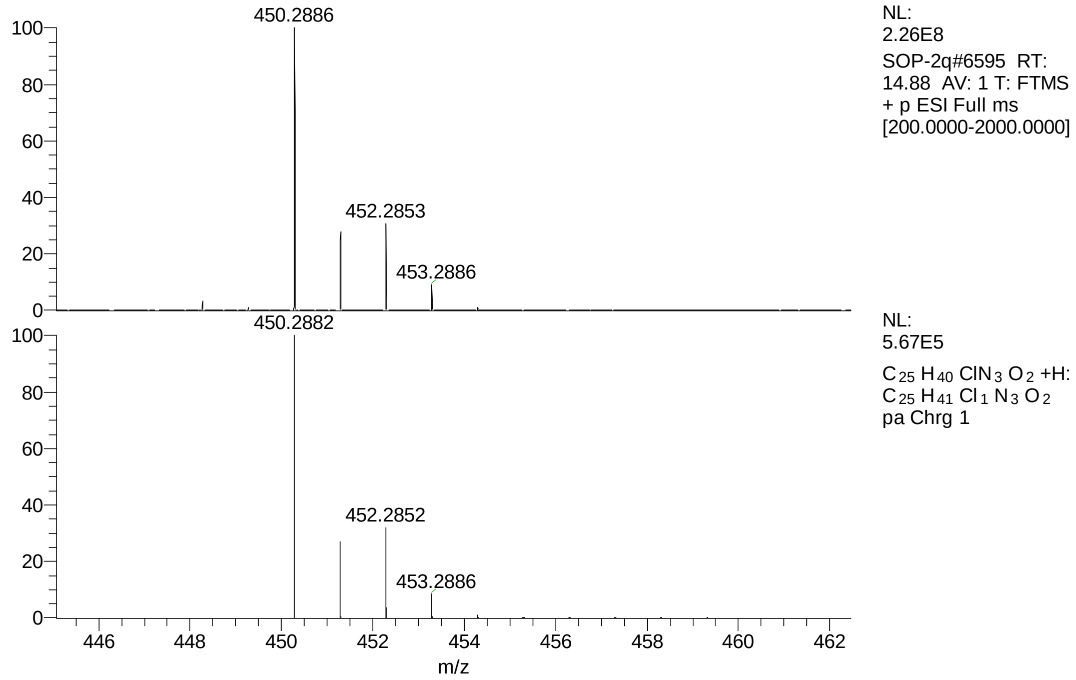

Figure S56. HR-ESI-MS spectrum of **SOP-2q**

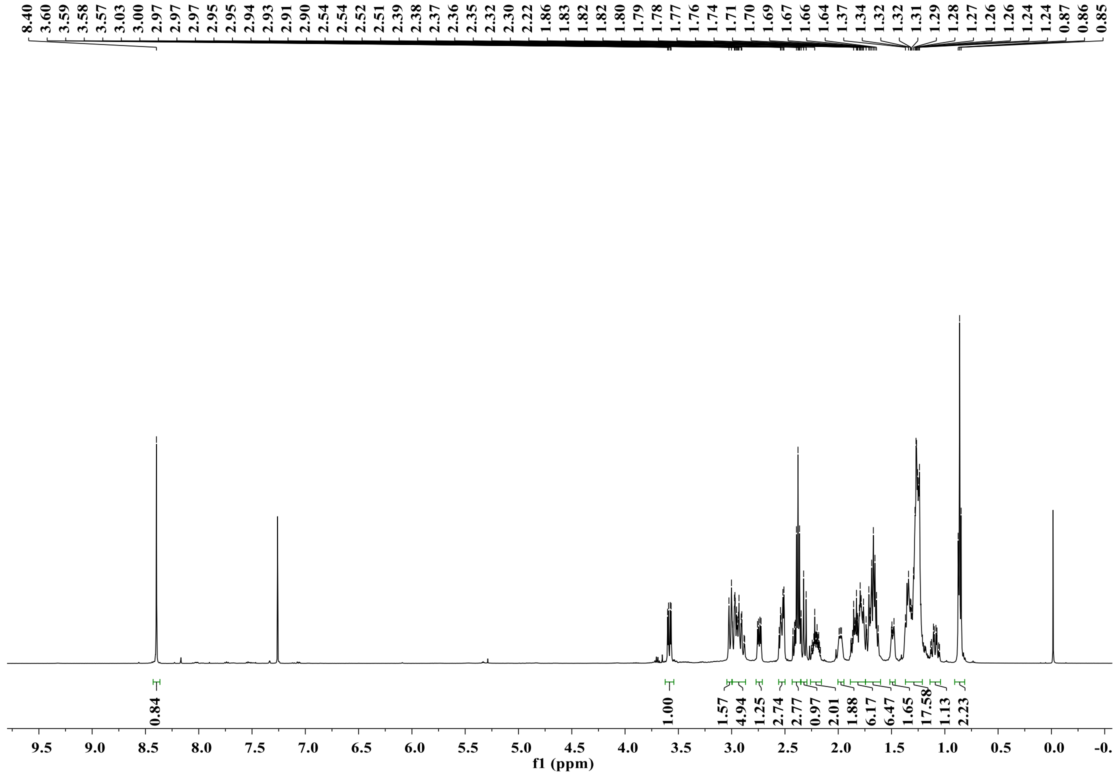

Figure S57. ^{1}H NMR spectrum of **SOP-2r**

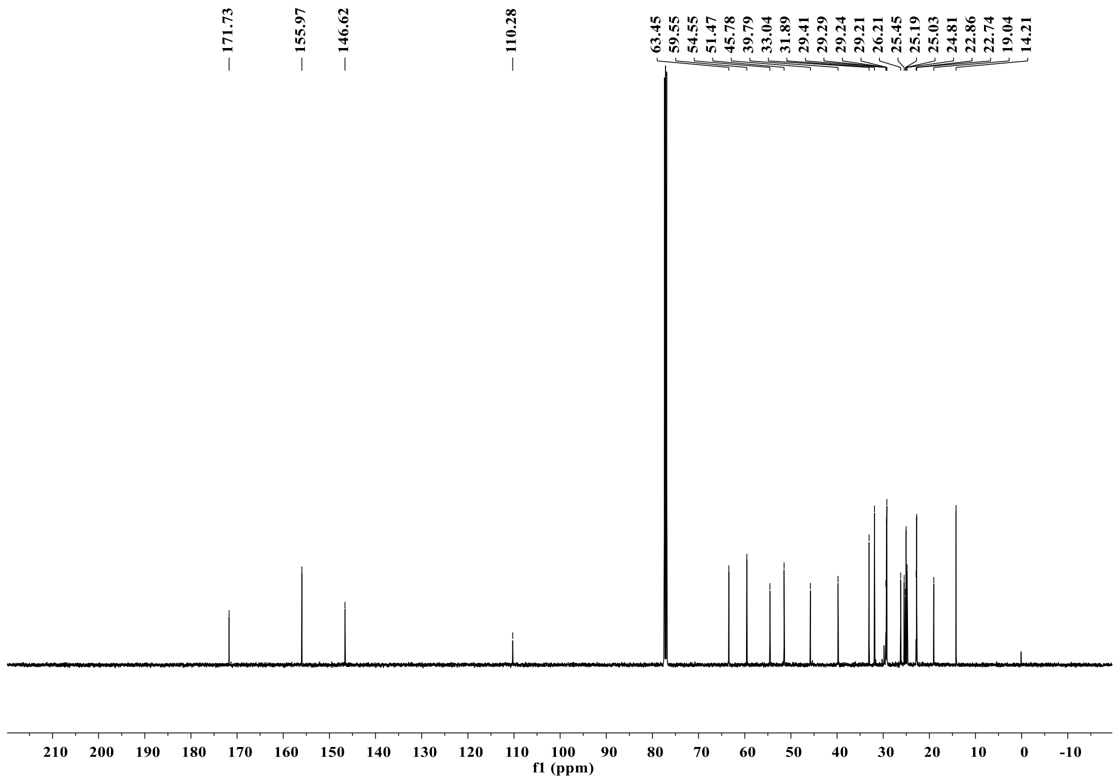

Figure S58. ^{13}C NMR spectrum of **SOP-2r**

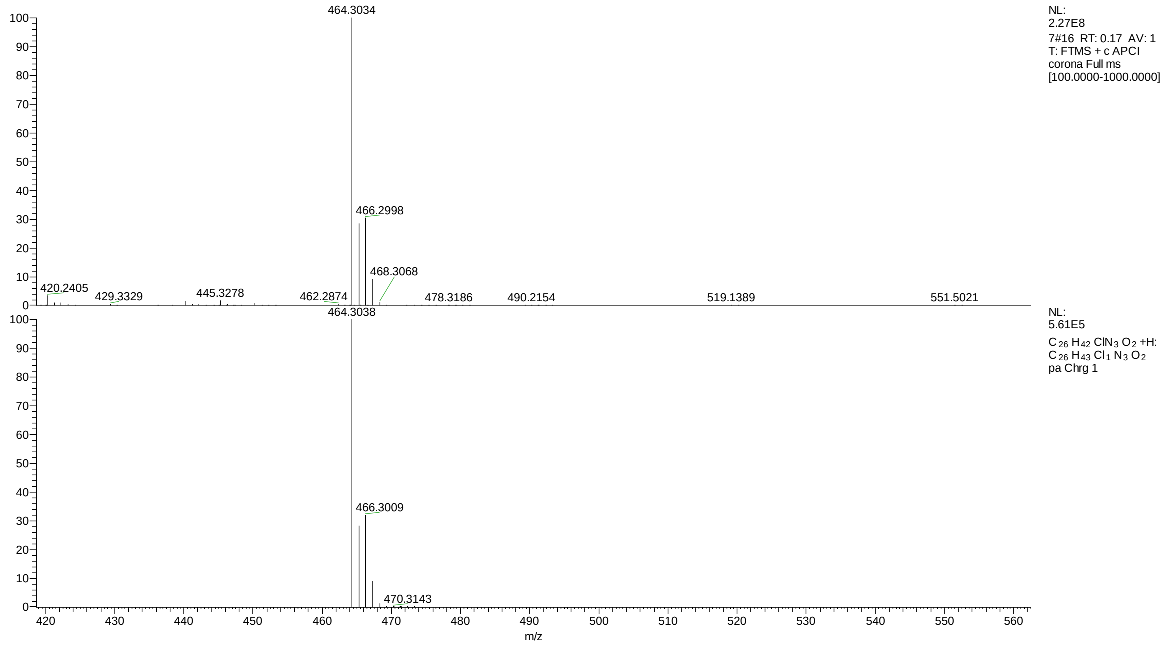

Figure S59. HR-ESI-MS spectrum of **SOP-2r**

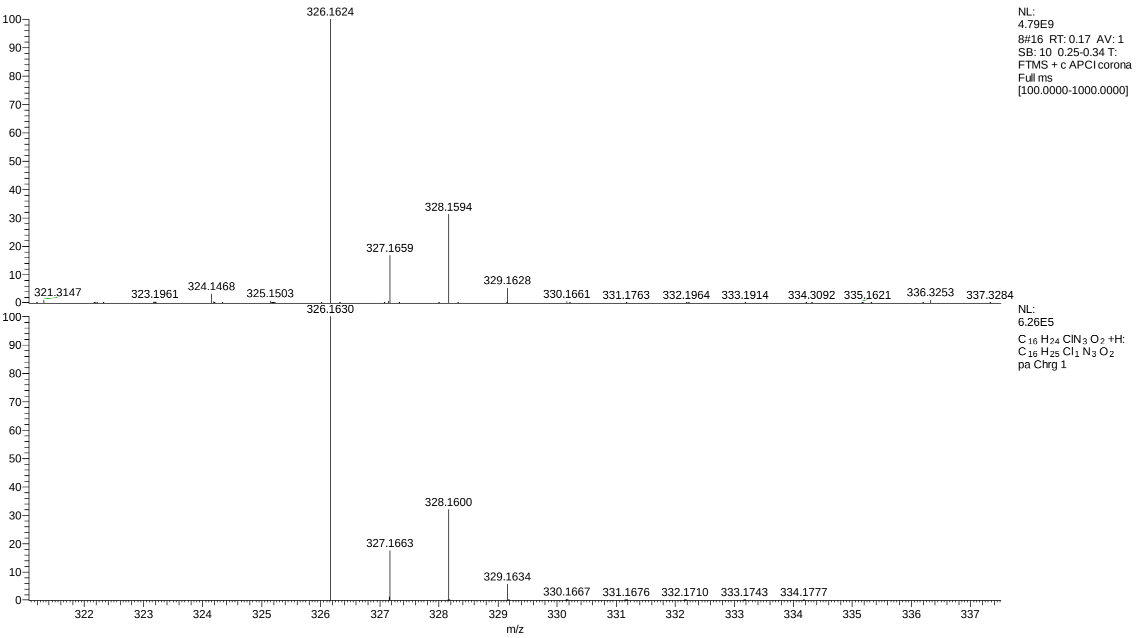

Figure S60. HR-ESI-MS spectrum of **SOP-3**

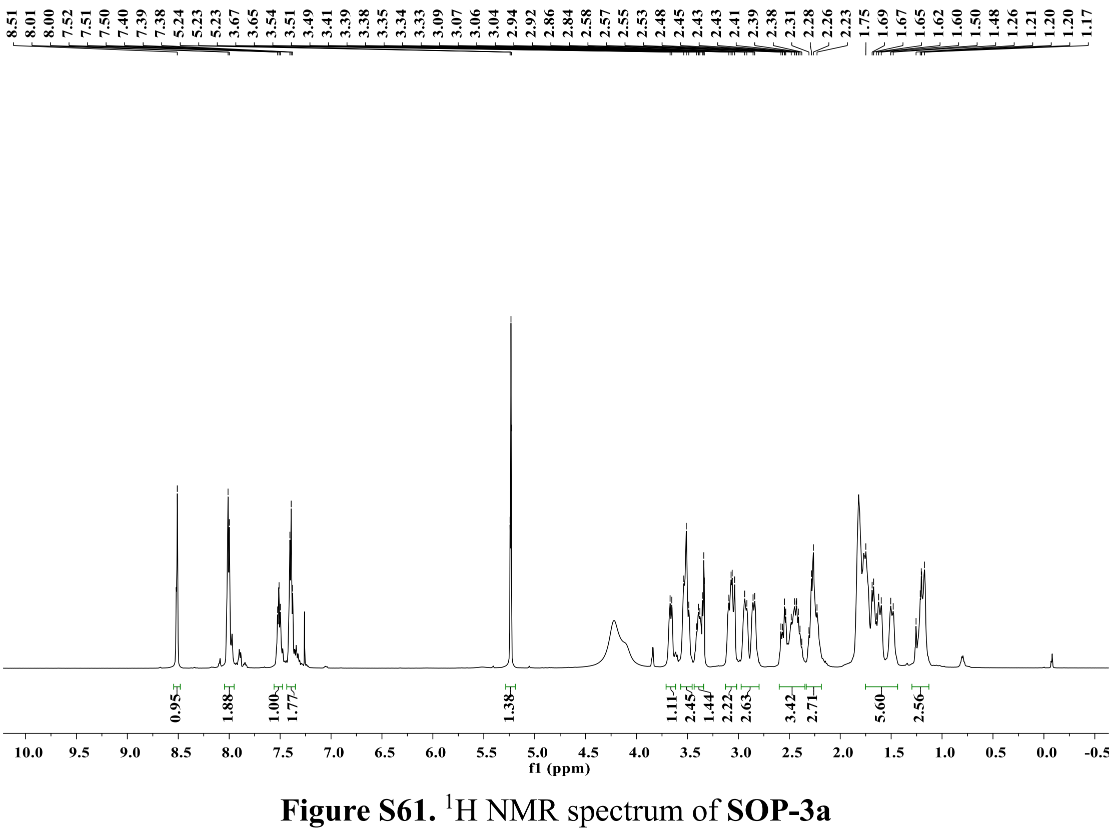

Figure S61. ^{1}H NMR spectrum of **SOP-3a**

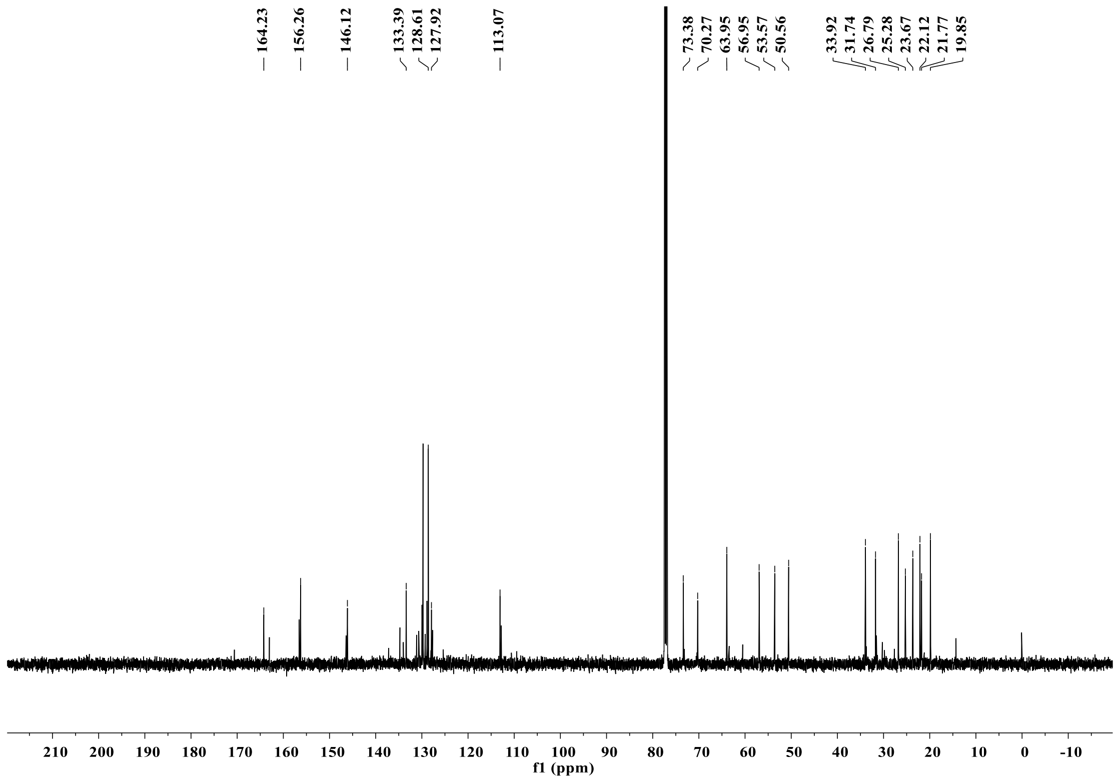

Figure S62. ^{13}C NMR spectrum of **SOP-3a**

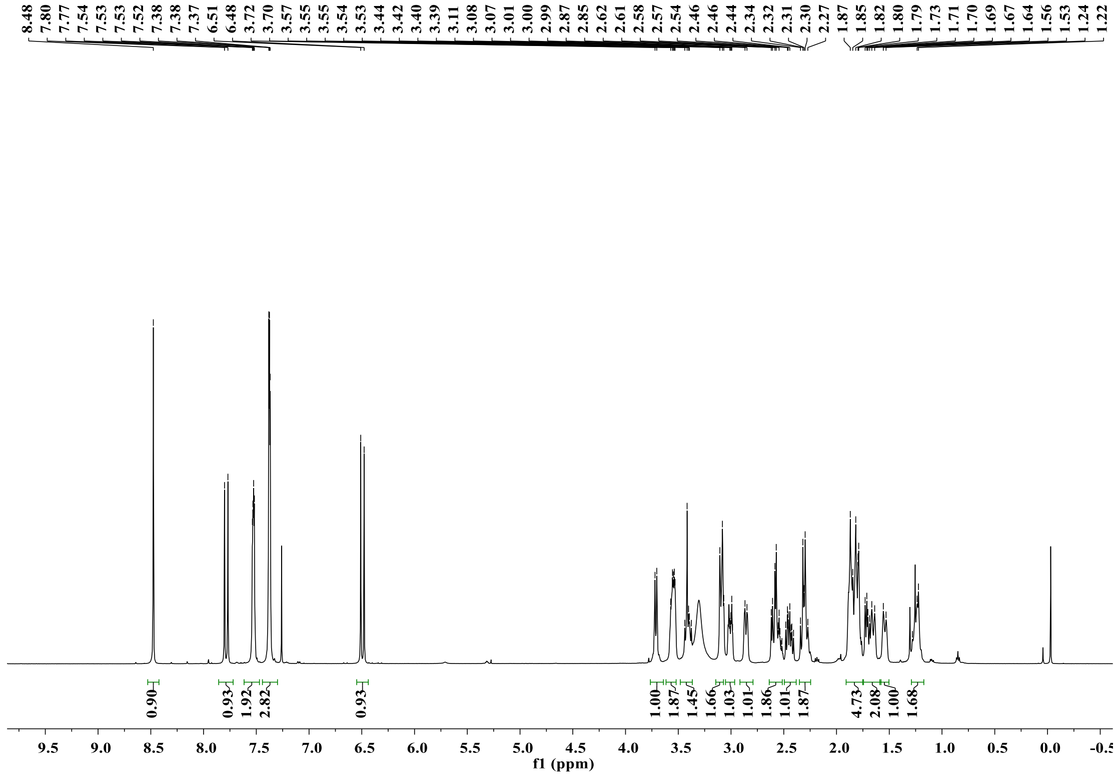

Figure S63. ^{1}H NMR spectrum of **SOP-3b**

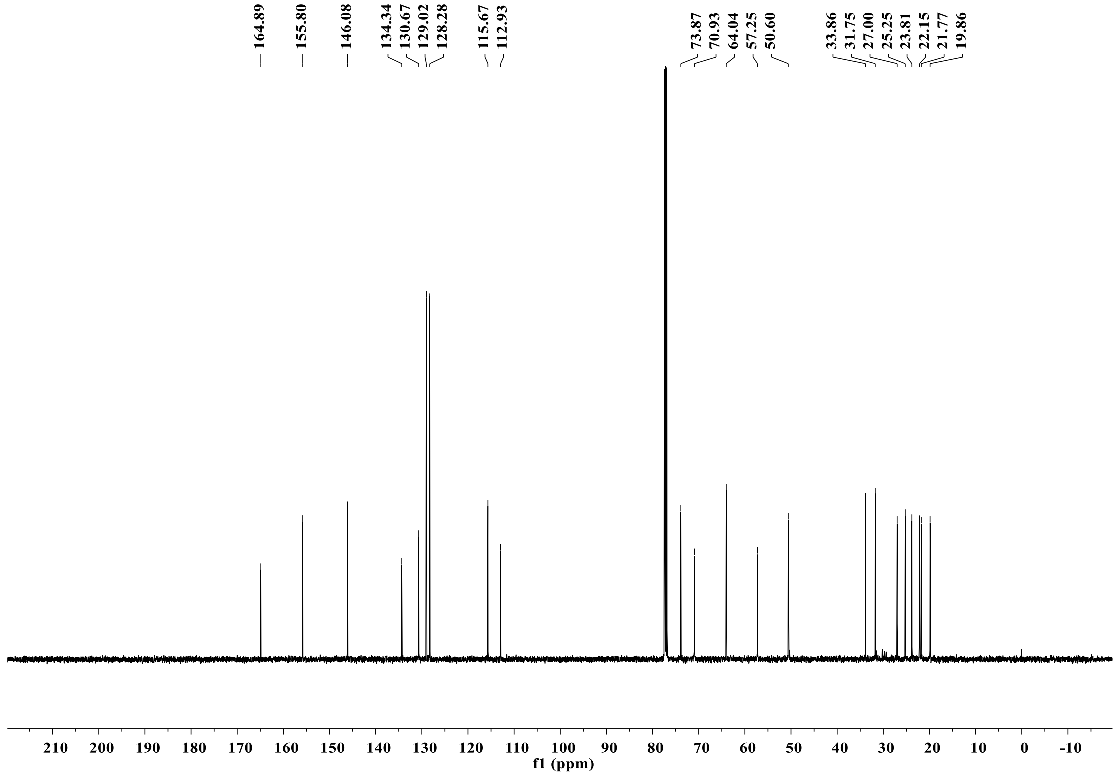

Figure S64. ^{13}C NMR spectrum of **SOP-3b**

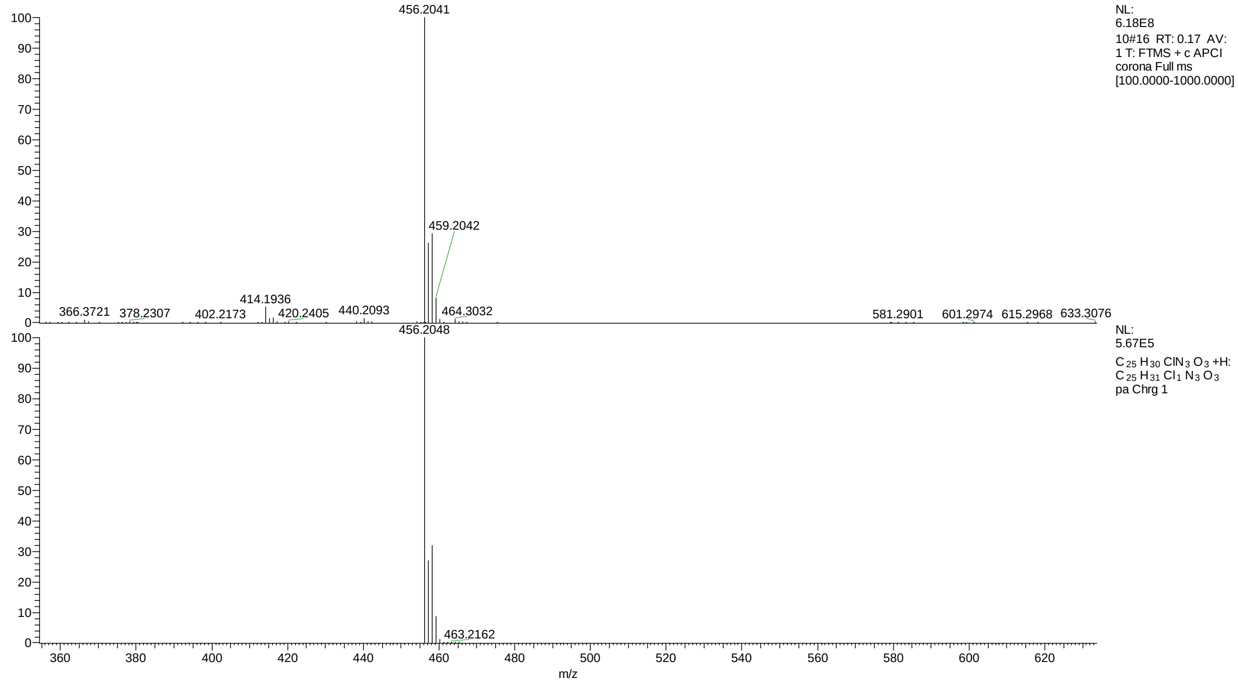

Figure S65. HR-ESI-MS spectrum of **SOP-3b**

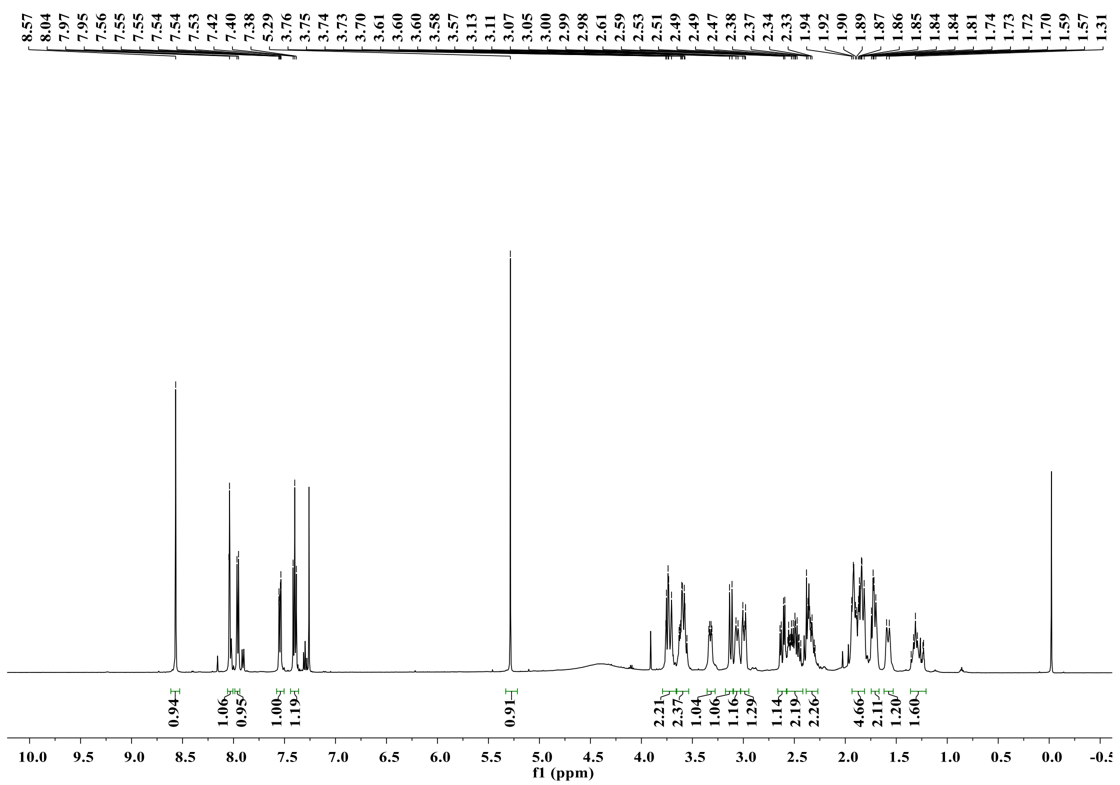

Figure S66. ^{1}H NMR spectrum of **SOP-3c**

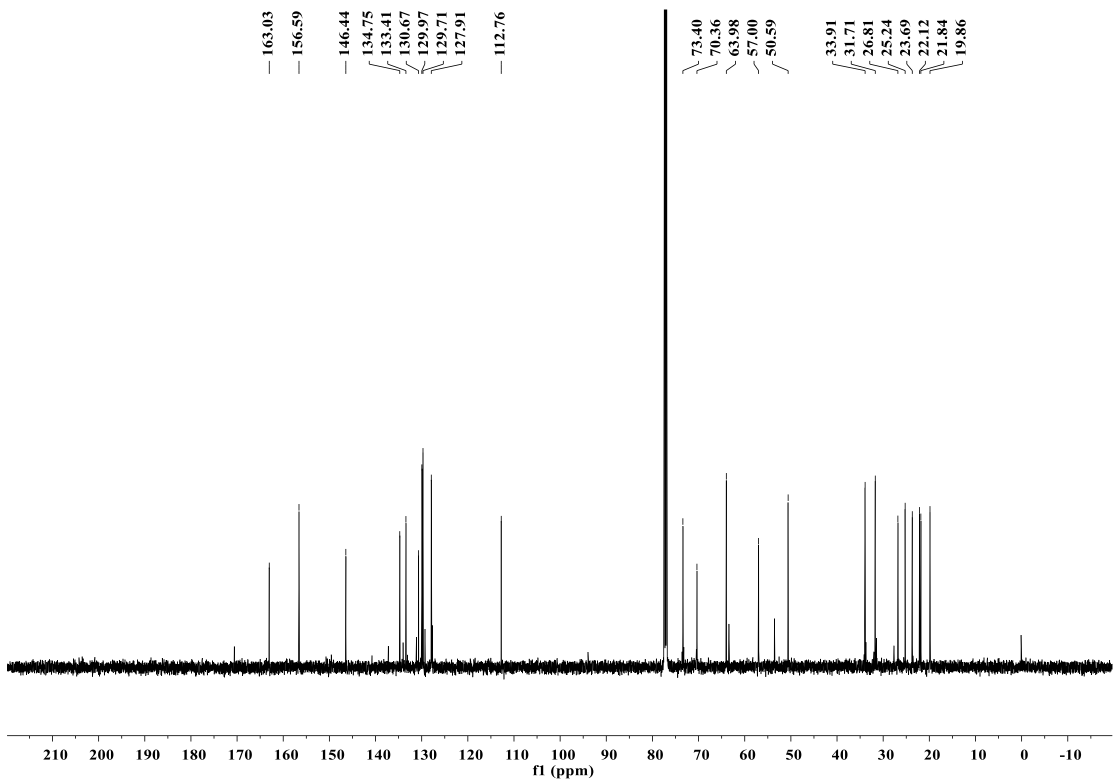

Figure S67. ^{13}C NMR spectrum of **SOP-3c**

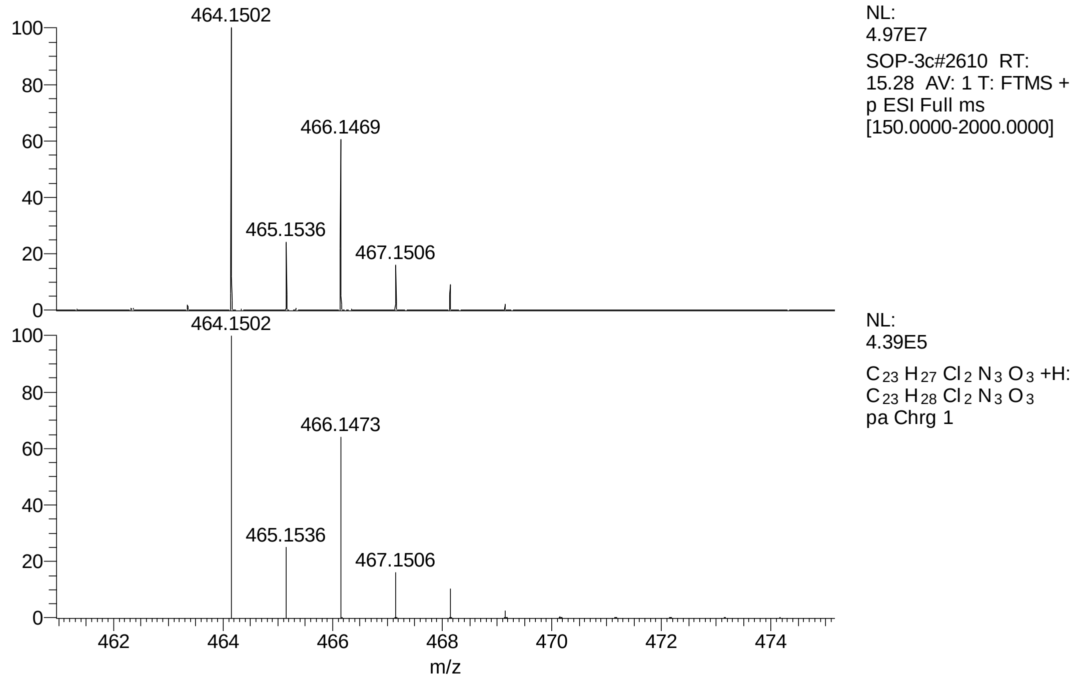

Figure S68. HR-ESI-MS spectrum of **SOP-3c**

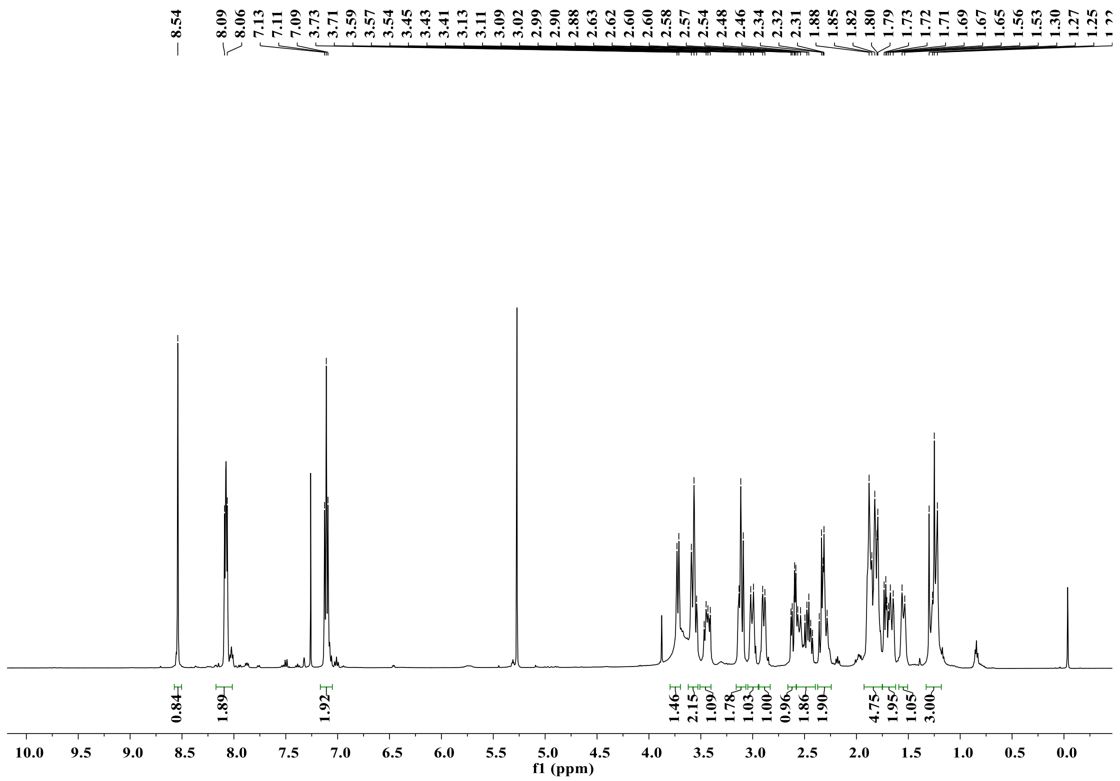

Figure S69. ^{1}H NMR spectrum of **SOP-3d**

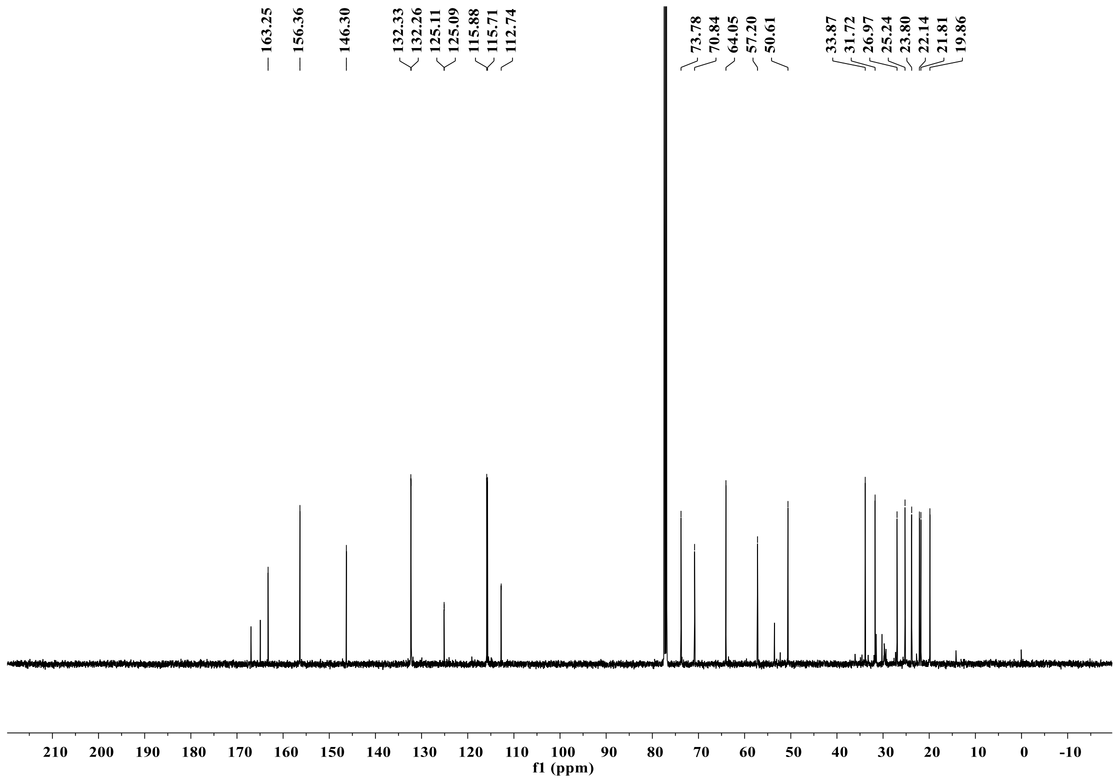

Figure S70. ^{13}C NMR spectrum of **SOP-3d**

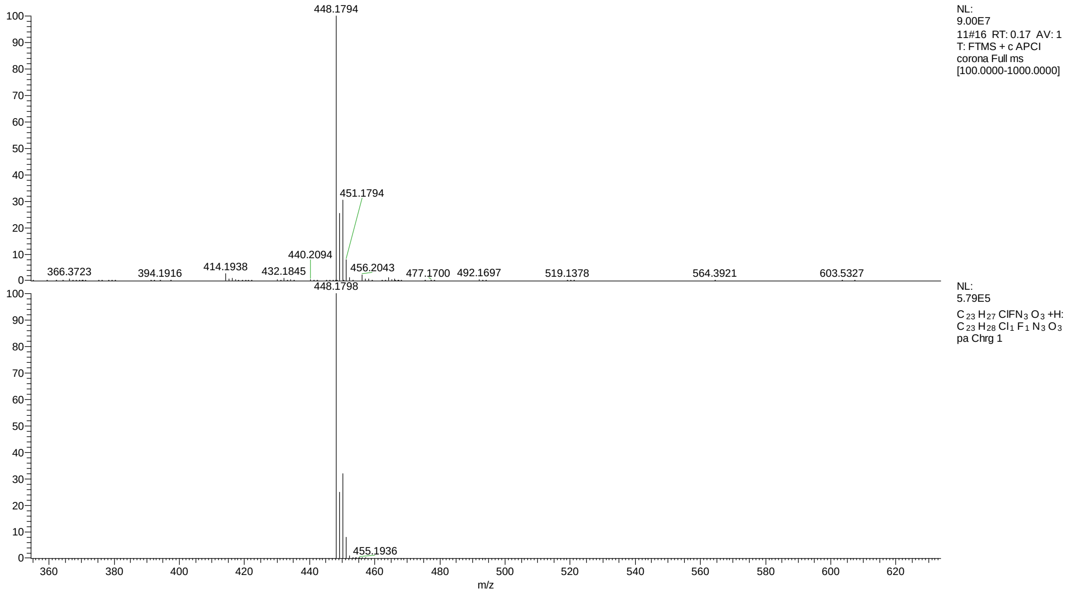

Figure S71. HR-ESI-MS spectrum of **SOP-3d**

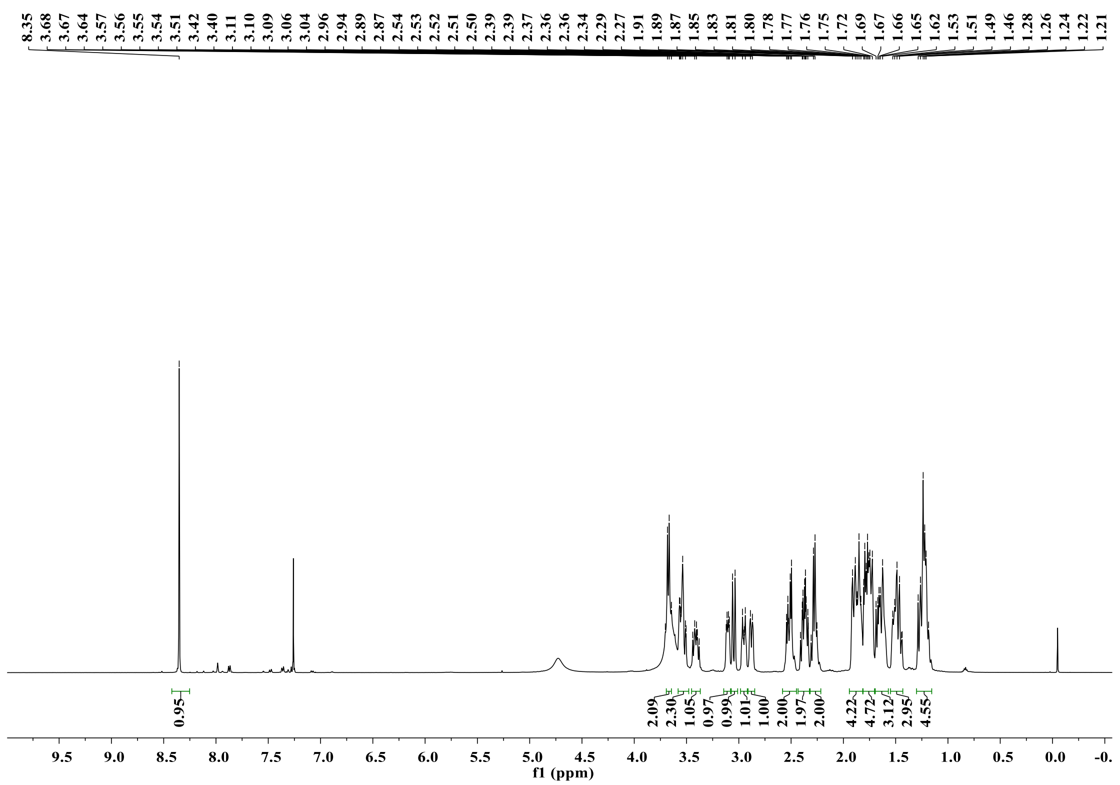

Figure S72. ^{1}H NMR spectrum of **SOP-3e**

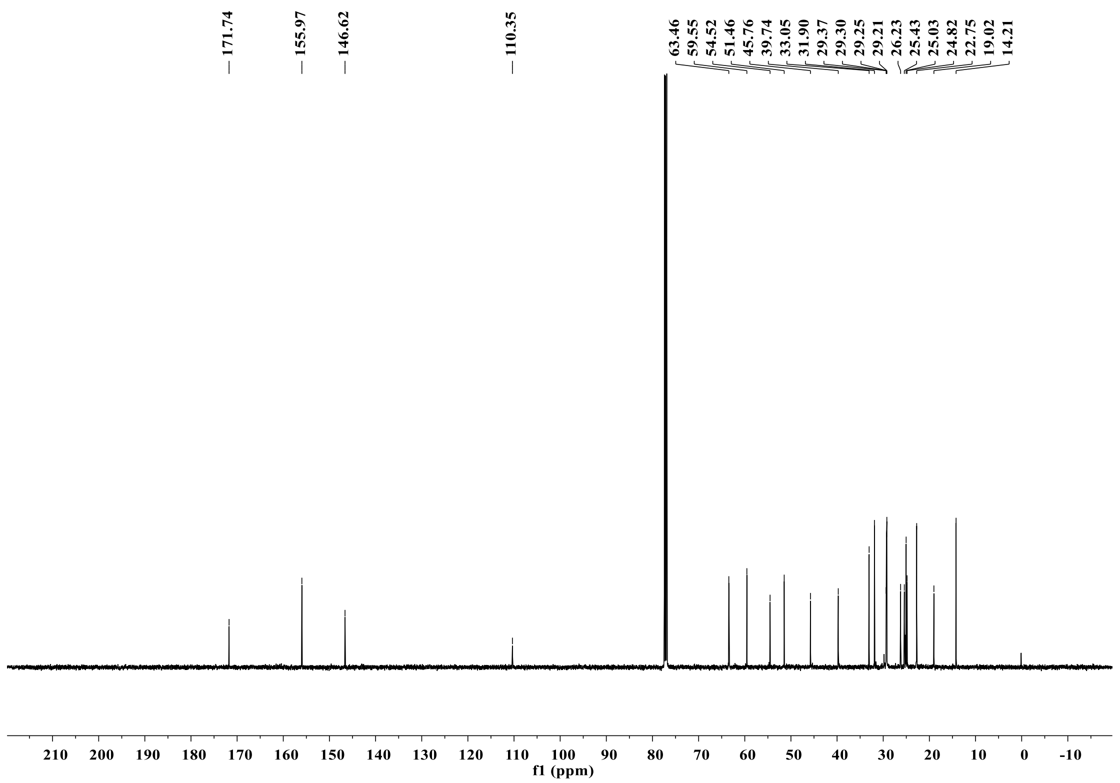

Figure S73. ^{13}C NMR spectrum of **SOP-3e**

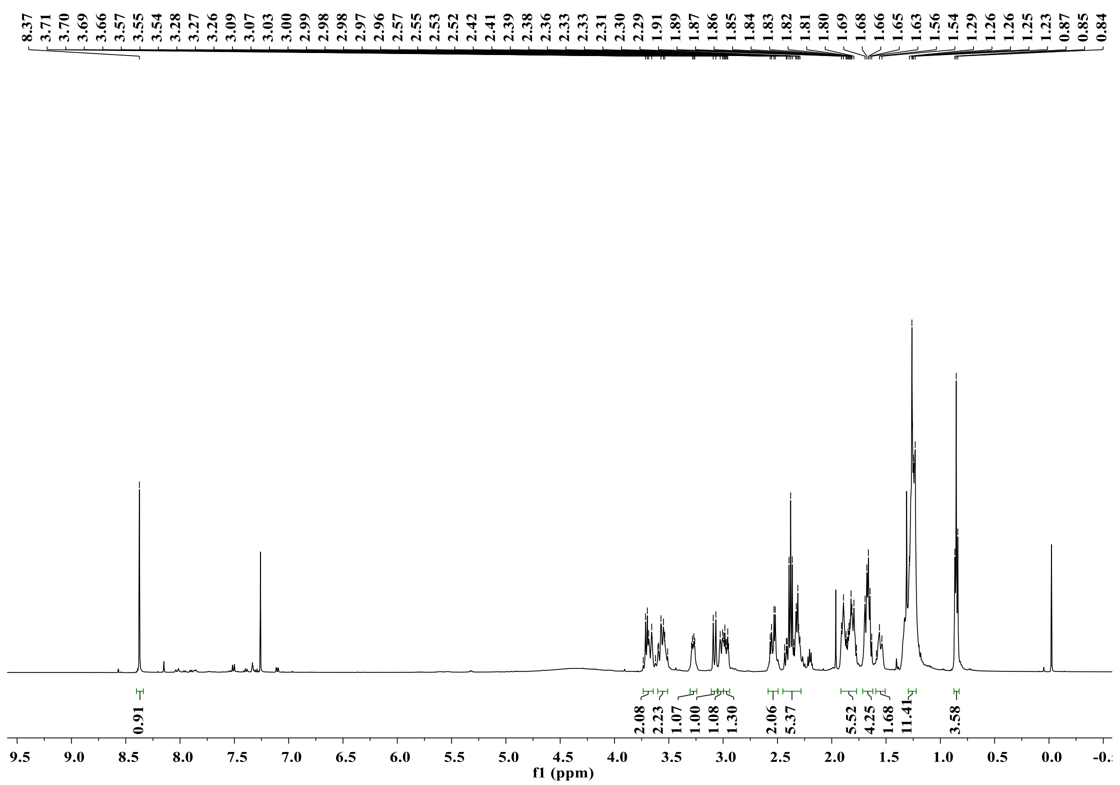

Figure S74. ^{1}H NMR spectrum of **SOP-3f**

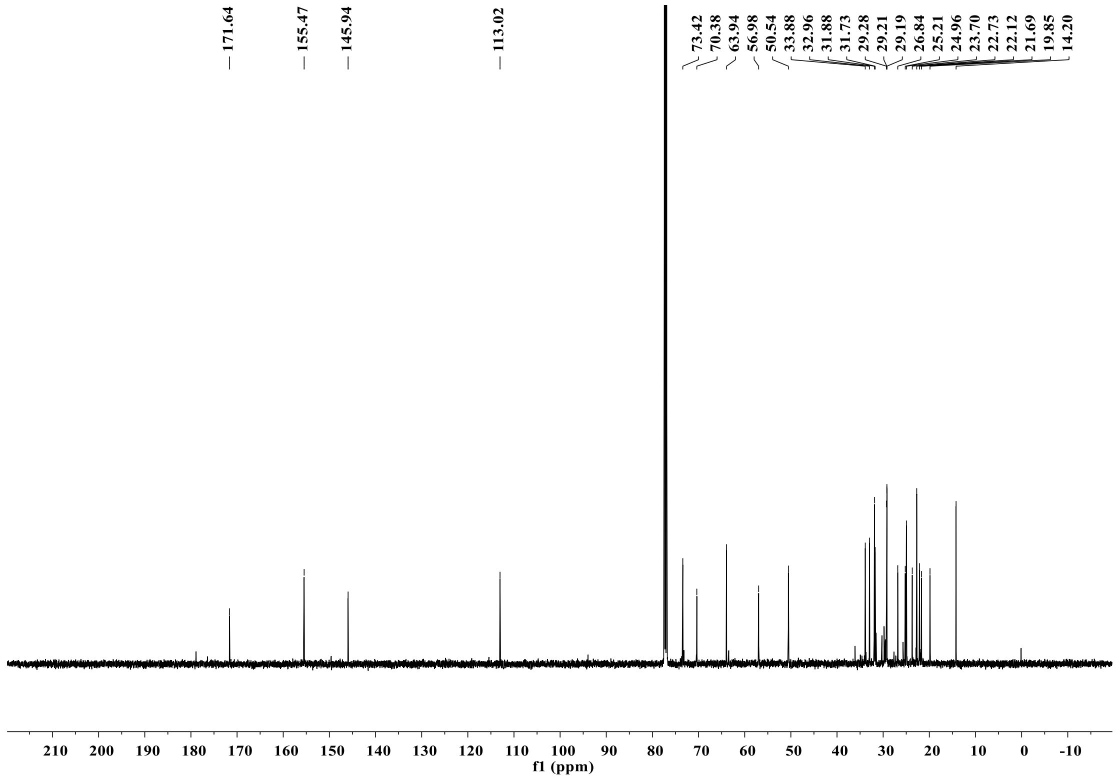

Figure S75. ^{13}C NMR spectrum of **SOP-3f**

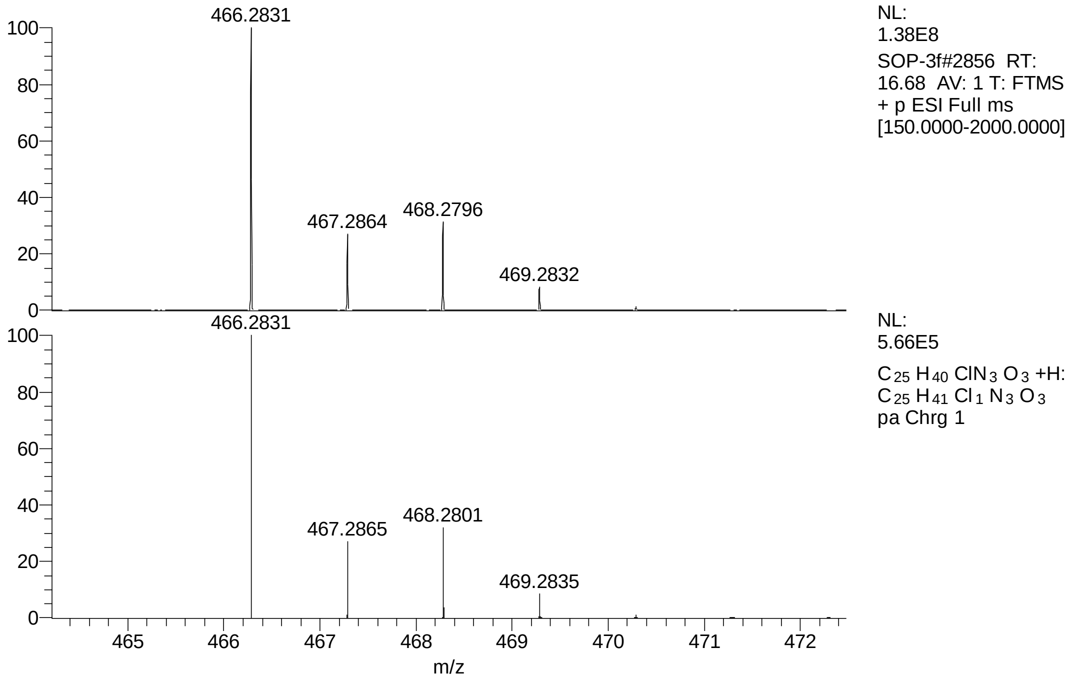

Figure S76. HR-ESI-MS spectrum of **SOP-3f**
